# Supplementary figures and images for: ATR, a DNA damage kinase, modulates DNA replication timing in Leishmania major
Source: PLoS Genet. 2025 Nov 24;21(11):e1011899. doi: 10.1371/journal.pgen.1011899 (PMC12677790; doi:10.1371/journal.pgen.1011899)

**A**

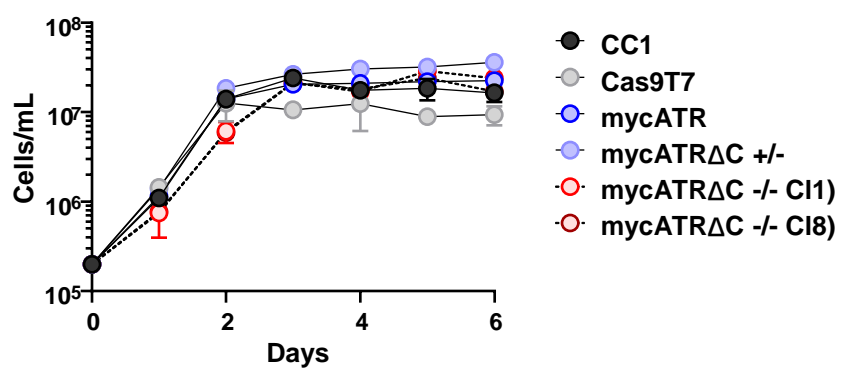

**B**

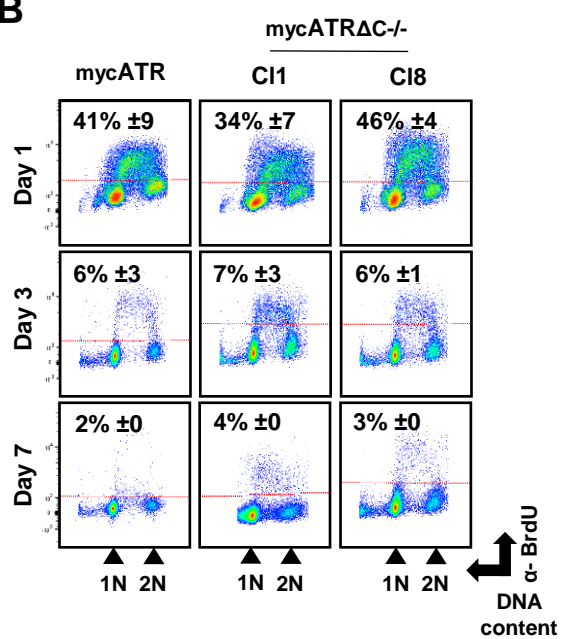

Supplementary Figure 1

Supplement: S1 Fig — (A) Growth curve of the indicated cells cultivated in HOMEM medium; cells were seeded at ~2x10^5^ cells.ml-1 at day 0; growth was evaluated for 6 days and cell density assessed every 24 hours; CC1 and Cas9T7 cells were used as control (Error bars ± SD; n = 2 experiments). (B) Representative pseudo-colour plots from a flow cytometry analysis to detect DNA synthesis in mycATR and mycATRΔC-/- (cl1 and cl8) cells. Cells were seeded at ~2x10^5^ cells.ml-1 at day 0; at the indicated time points an aliquot of each cell line was incubated with IdU for 30 min and IdU detected using α-BrdU under denaturing conditions; DNA was stained with propidium iodate; ~ 30,000 cells were analysed per sample; 1N and 2N indicate single and double DNA content; dashed red lines indicate the threshold used to discriminate negative from IdU-positive events; inset numbers indicate total percentage of IdU-positive events in the whole population (n = 2 experiment). (PDF) [file pgen.1011899.s001.pdf]

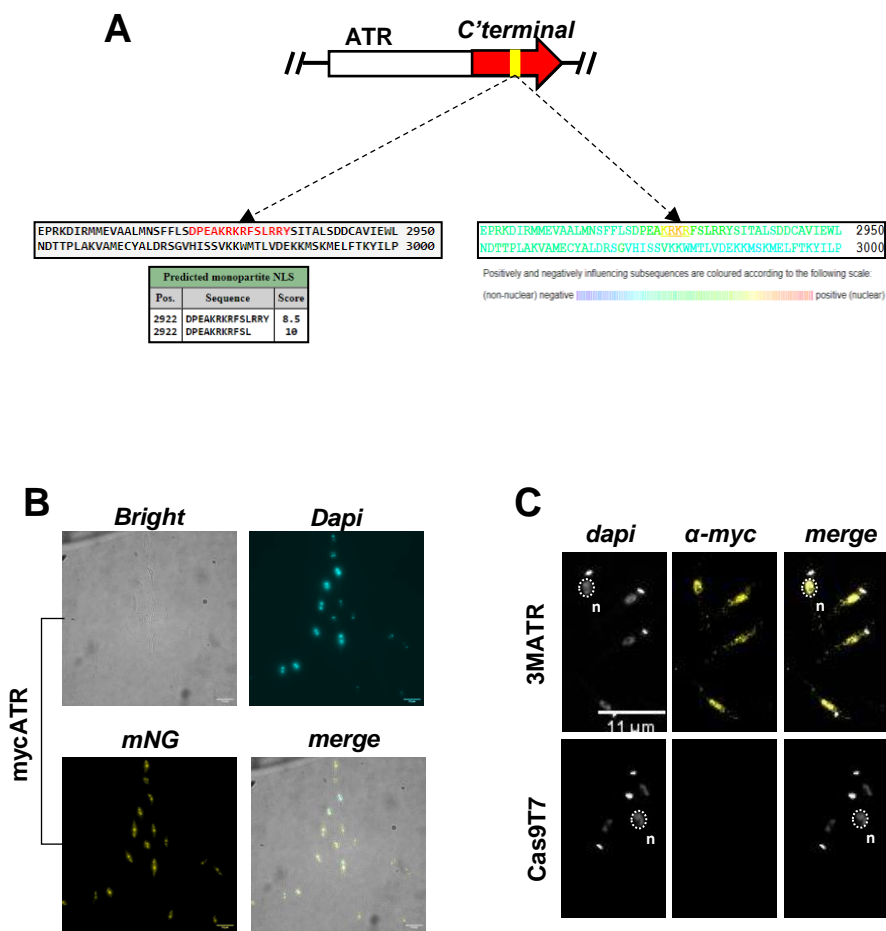

Supplementary Figure 2

Supplement: S2 Fig — (A) Schematic representation of the region where we predict a possible Nuclear Location Signal (NLS). Predicted ATR protein sequence was accessed through Tritryp.pdb (LmjF.32.1460), and possible NLS signals assessed using two available software (http://nls-mapper.iab.keio.ac.jp/cgi-bin/NLS_Mapper_y.cgi) and (https://nucpred.bioinfo.se/cgi-bin/single.cgi). (B) Representative images of the sub-cellular localization of mNeongreen signal, which is N-terminally added to ATR in mycATR cells; scale = 13 μm. Images were captured using a SP6 microscope (Leica). DNA, shown in cyan, was stained with PBS and Hoescht solution for 10 min at 27°C followed by image acquisition; the mNeongreen signal is in yellow. (C) Representative images acquired on a LSM880 Zeiss confocal microscope from Z stack images of 3MATR (cell line with only 3x myc tag at N’terminal of ATR gene). Cells were fixed with 3% formaldehyde and stained with α-myc in yellow; genomic DNA stained was with DAPI in gray (Bar = 11 um). (PDF) [file pgen.1011899.s002.pdf]

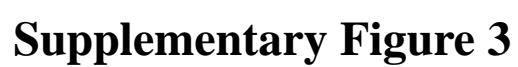

Supplement: S3 Fig — EdU intensity (A.U.) from positive EdU mycATR or mycATRΔC-/- cells in each condition showed in Fig 3E. An area was drawn around each EdU positive cell’s nucleus based on the DAPI stain and the EdU signal intensity was measured using ImageJ software, (Error bars ± SD; n = 2 experiments, p < 0.05; ** p < 0.005; *** p < 0.001; **** p < 0.0001. Unpaired t-test). (PDF) [file pgen.1011899.s003.pdf]

Supplementary Figure 4

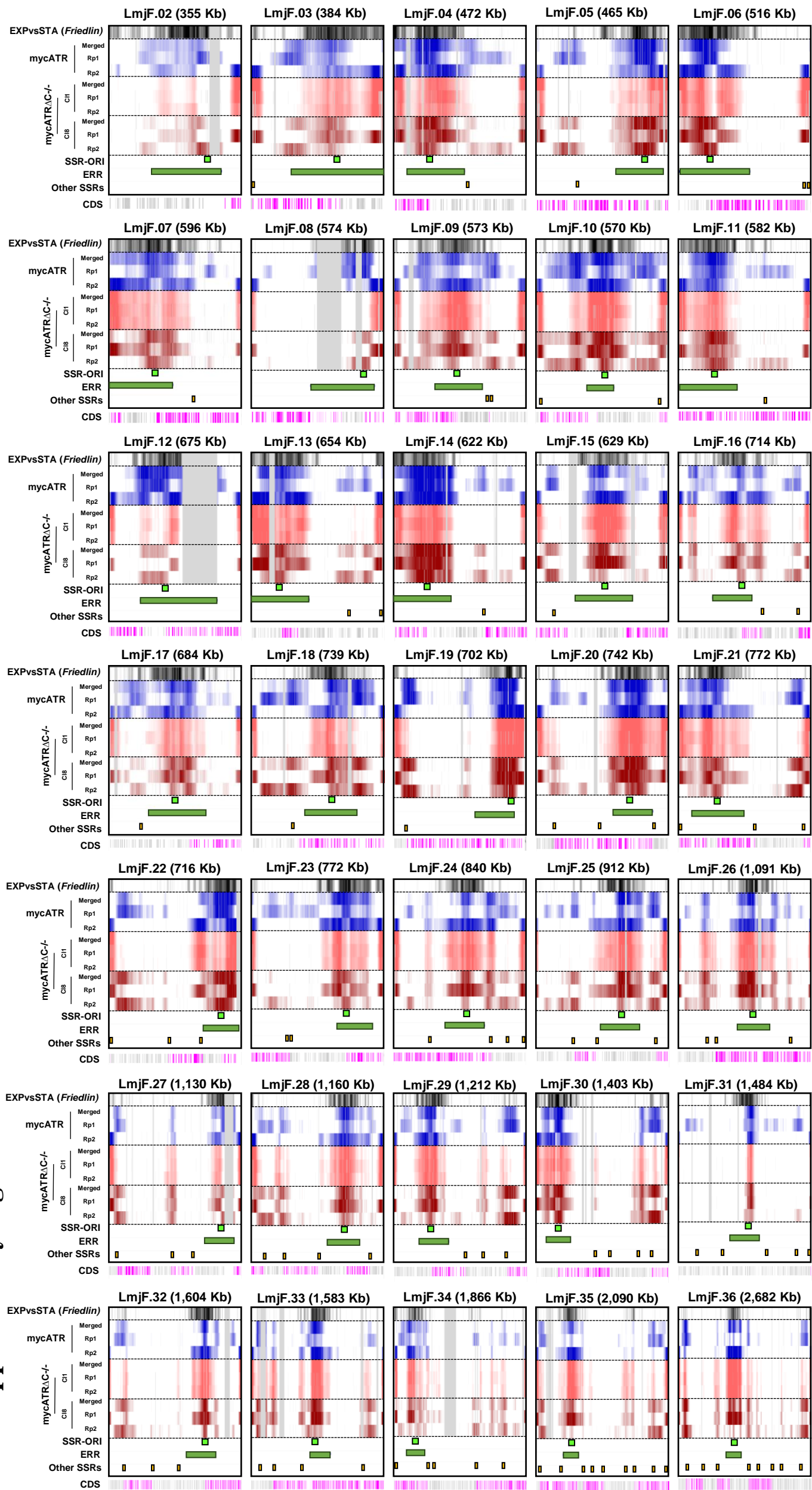

Supplement: S4 Fig — Representative snapshots of all chromosomes (excluding chromosome 01, due an amplification in the STA control), showing DNA replication timing (MFA-seq signal) using exponentially growing (untreated/NT) mycATR (blue) and mycATRΔC-/- (cl1 and cl8) cells (light coral and firebrick, respectively. Signals in two replicates (rp1 and rp2) and the merged signal for each cell are shown; positive (coloured) and negative (white) values indicate early and late replicating regions, respectively; the top heatmap (black) represents a previous published MFA-seq from wildtype L. major Friedlin [65]; SSR-Ori (light green), early replication regions (ERR) (dark green) and other SSRs (orange) positions in each chromosome are displayed; the bottom track indicates annotated CDSs (gray: transcribed from left to right; pink: transcribed from right to left), grey regions represent removed areas which have poor mapping quality (see Methods). (PDF) [file pgen.1011899.s004.pdf]

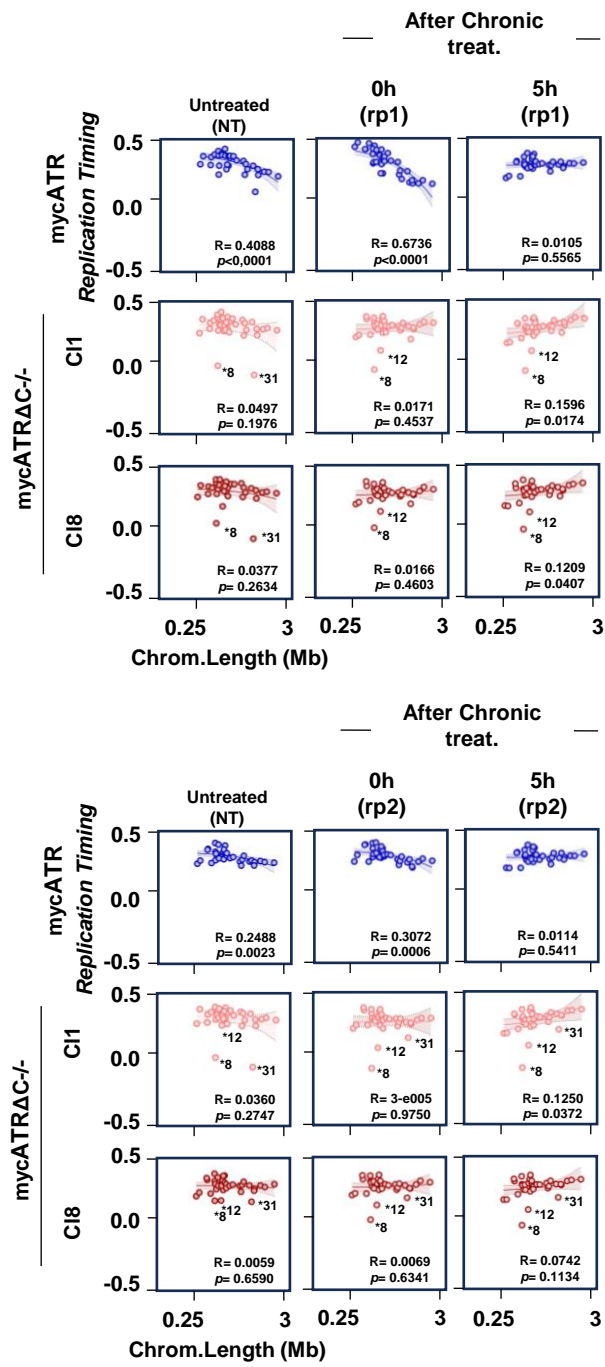

**Supplementary Figure 5**

Supplement: S5 Fig — Linear regression analysis for each replicate (rp1 and rp2), showing correlation between chromosome size (x axis) and chromosome-averaged MFA-seq signal (DNA replication timing, y axis) in untreated (NT) mycATR and mycATRΔC-/- (cl1 and cl8) cells post-release from chronic HU treatment (0 and 5 hours) in; R squared and P values are indicated within each panel. (PDF) [file pgen.1011899.s005.pdf]

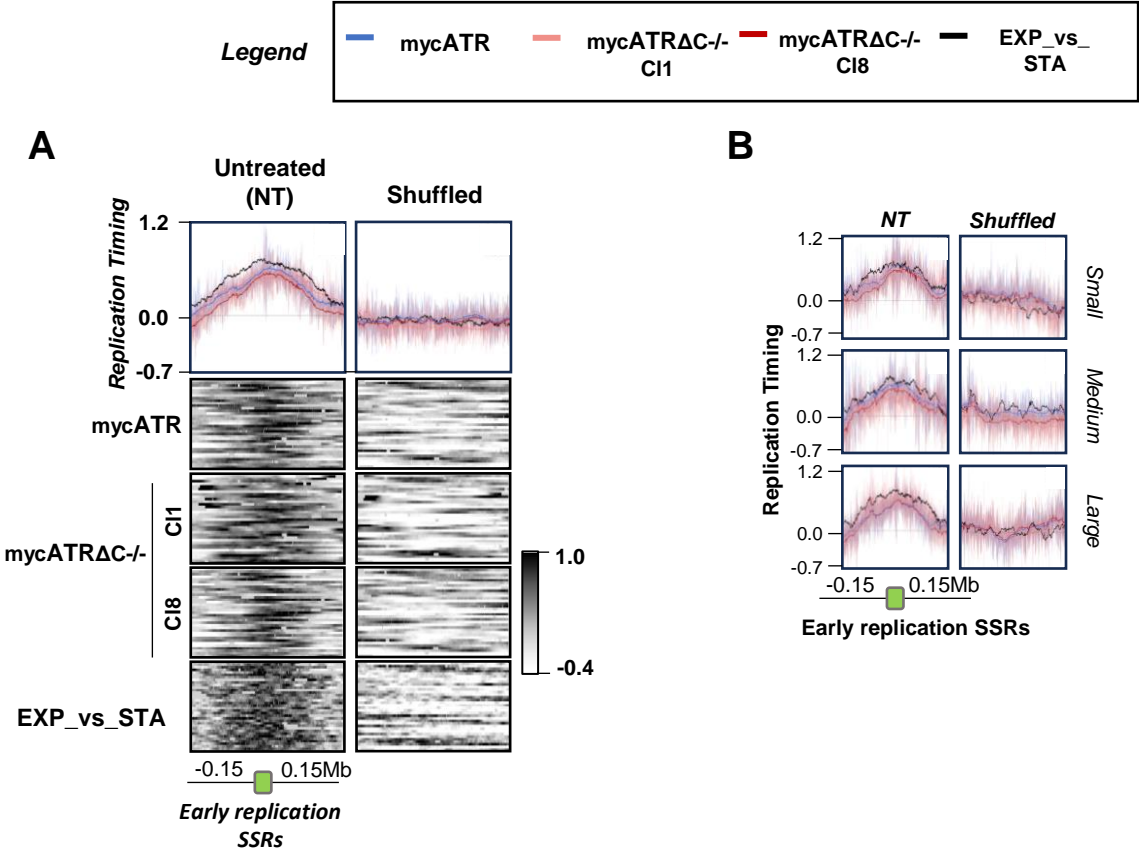

Supplementary Figure 6

Supplement: S6 Fig — (A) Metaplots of global MFA-seq signal in mycATR (blue) and mycATRΔC-/- (cl1 and cl8; light coral and firebrick, respectively) cells, 0 and 5 hours post release from chronic HU treatment; the black line represents previously published MFA-seq from wildtype L. major Friedlin [65]. Signal was plotted over the Early replication SSRs from each chromosome (n = 36, Centre) ± 0.15 Mb of upstream and downstream sequence; the line indicates the mean, and the light-coloured areas around the line indicates the standard deviation between the two experimental replicas (SD); metaplots from shuffled genome regions were used as control. Below: the colourmaps represent the MFA-seq signal in each early replicating SSR in each sample; metaplots and heatmaps from shuffled genome regions were used as control. (B) Metaplots of global MFA-seq signal, as in (A), after subclassifying the chromosomes according to length: Smaller (chromosomes 01 – 11 and 14), Medium (12, 13, 151617181920212223 – 24) and Larger (2526272829303132333435 – 36); metaplots from shuffled genome regions were used as control. (PDF) [file pgen.1011899.s006.pdf]

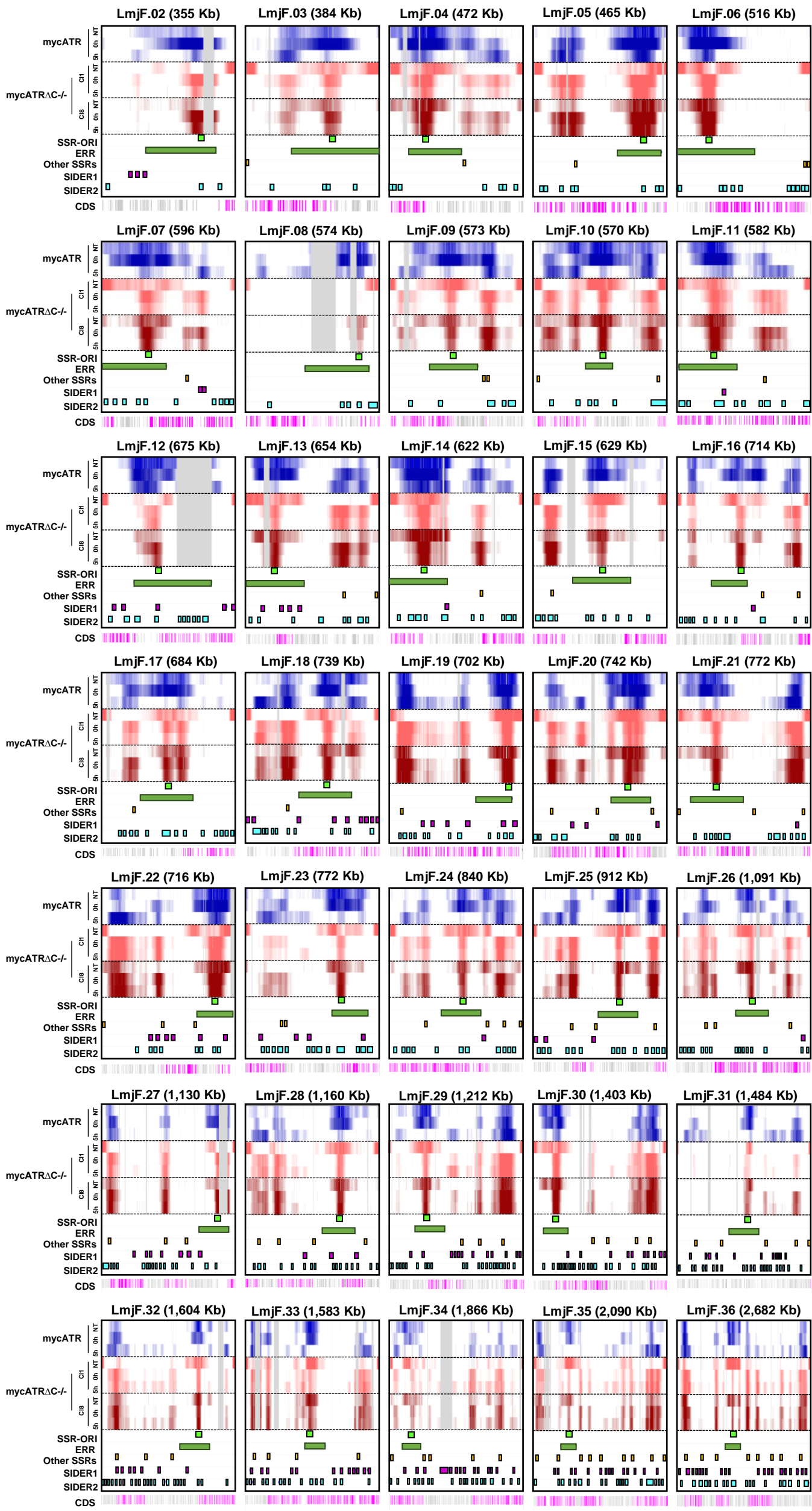

Supplement: S7 Fig — Representative snapshots of all chromosomes (excluding chromosome 01 due an amplification on the STA control), showing DNA replication timing (MFA-seq) in mycATR (blue) and mycATRΔC-/- (cl1 and cl8light coral and firebrick, respectively) cells that were either exponentially growing (untreated/NT) or post-release from chronic HU treatment (0 and 5 hours); the signal represents the merge of the two replicates after normalisation (S8 Fig); positive (coloured) and negative (white) values indicate early and late replicating regions, respectively; SSR-Ori (light green), early replication regions (ERR) (dark green), other SSRs (orange), SIDER1 (purple) and SIDER2 (cyan) positions are displayed; the bottom track indicates annotated CDSs (gray: transcribed from left to right; pink: transcribed from right to left), grey regions represent removed areas which have poor mapping quality. (PDF) [file pgen.1011899.s007.pdf]

Supplementary Figure 8

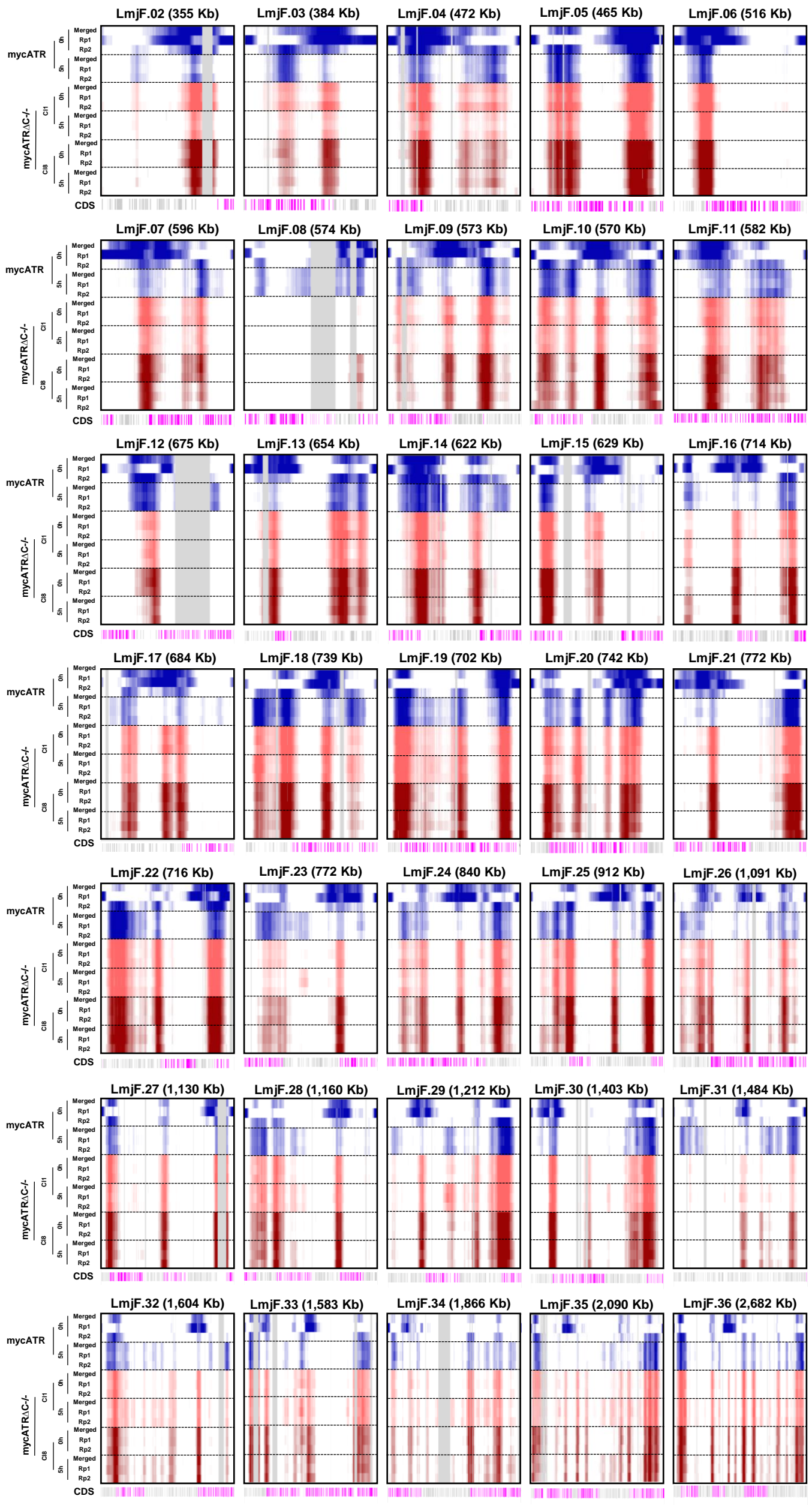

Supplement: S8 Fig — Representative snapshots from the experimental replicas used in S7 Fig (DNA replication timing (MFA-seq) in mycATR (blue) and mycATRΔC-/- (cl1 and cl8, light coral and firebrick, respectively) cells that were either exponentially growing (untreated/NT) or post-release from chronic HU treatment (0 and 5 hours)). (PDF) [file pgen.1011899.s008.pdf]

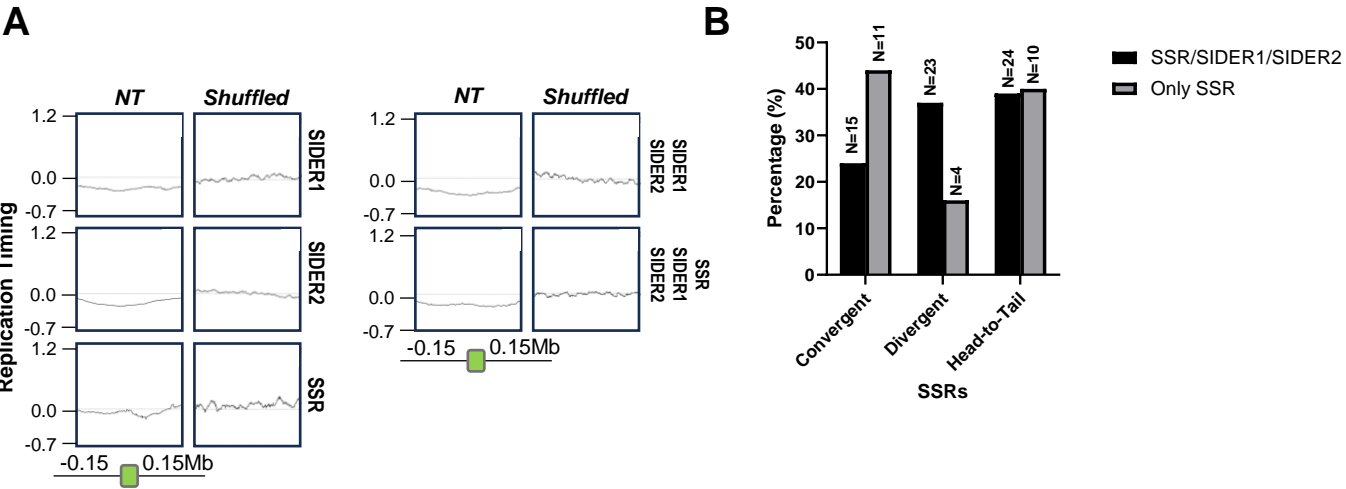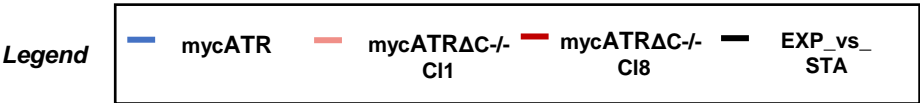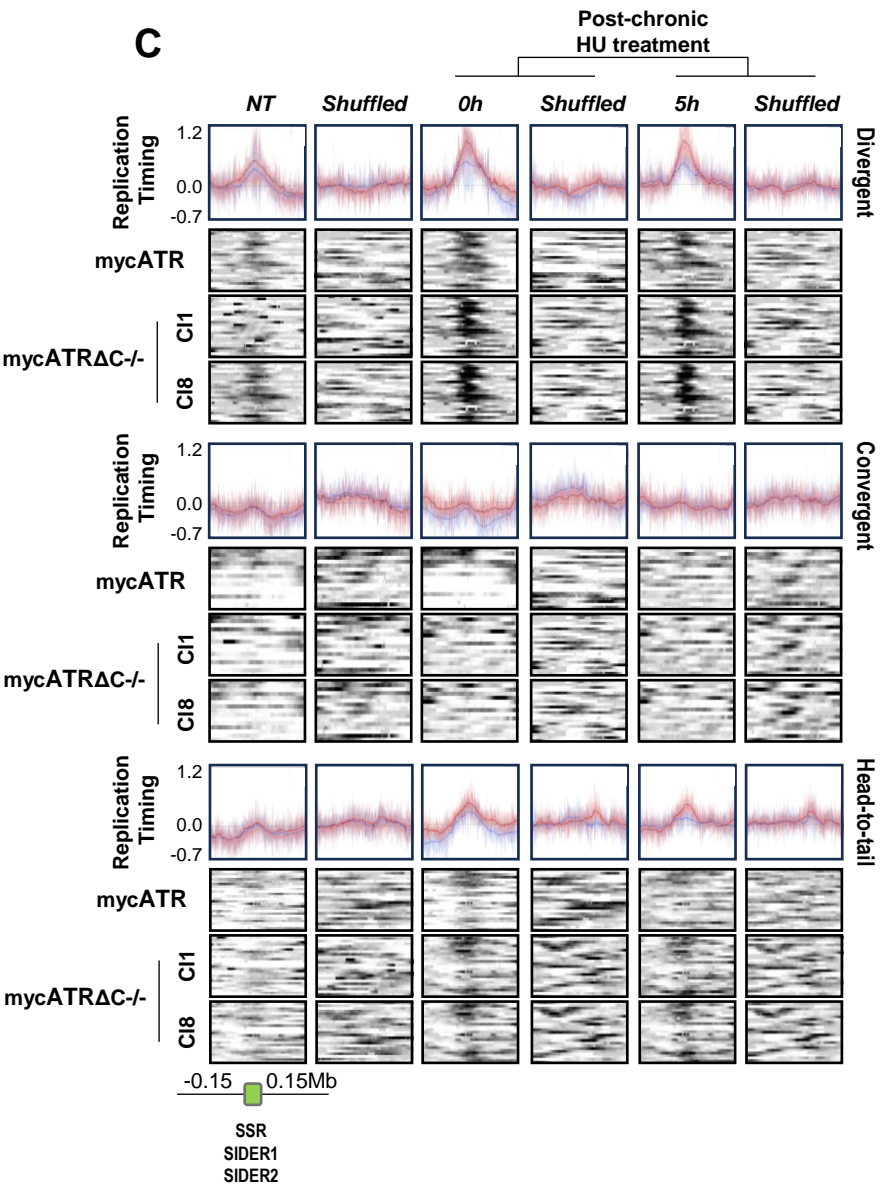

Supplementary Figure 9

Supplement: S9 Fig — (A) MFA-seq signal from a previous report [65] in wildtype cells (L. major Friedlin), plotted around the regions described in (6D) ± 0.15 Mb of upstream and downstream sequence. (B) Graphic showing the percentage and number of SSR/SIDER1/SIDER2, or SSRs alone (Only SSR) in each type of Strand Switch Region (convergent, divergent and head-to-tail). (C) Metaplots of global MFA-seq signal in mycATR and mycATRΔC-/- (cl1 and cl8) cells after chronic HU treatment (0 and 5 hours) around each type of SSR (COnVergent, DIVergent, HT head-to-tail) that are in proximity with either SIDER1 or SIDER2 ± 0.15 Mb of upstream and downstream sequence. Metaplots from shuffled genome regions were used as control. (PDF) [file pgen.1011899.s009.pdf]
