# Supplementary material for: ATR, a DNA damage kinase, modulates DNA replication timing in Leishmania major
Source: PLoS Genet. 2025 Nov 24;21(11):e1011899. doi: 10.1371/journal.pgen.1011899 (PMC12677790; doi:10.1371/journal.pgen.1011899)
Supplement: S1 Table — (PDF) [file pgen.1011899.s010.pdf]

ERR (early replication region)				Regions of Overlap of SSR/SIDER1/SIDER2				Regions of Overlap of SIDER1/SIDER2			
Chromosome	start	end	Length (Mb)	Chromosome	start	end	Length (Mb)	Chromosome	start	end	Length (Mb)
LmjF.01	130000	266499	0.136499	LmjF.05	129039	150332	0.021293	LmjF.12	29523	71279	0.041756
LmjF.02	118000	302999	0.184999	LmjF.06	479605	500605	0.021	LmjF.12	99449	143768	0.044319
LmjF.03	133000	382499	0.249499	LmjF.09	412919	450678	0.037759	LmjF.18	39667	80983	0.041316
LmjF.04	75000	254499	0.179499	LmjF.10	509190	570718	0.061528	LmjF.18	689626	710373	0.020747
LmjF.05	297000	446999	0.149999	LmjF.11	377605	401106	0.023501	LmjF.19	169977	190039	0.020062
LmjF.06	3000	282499	0.279499	LmjF.13	479054	490469	0.011415	LmjF.19	199633	230664	0.031031
LmjF.07	2000	290999	0.288999	LmjF.14	419696	430215	0.010519	LmjF.19	299808	320492	0.020684
LmjF.08	256000	535499	0.279499	LmjF.15	69719	90683	0.020964	LmjF.19	358928	421499	0.062571
LmjF.09	177000	379499	0.202499	LmjF.16	449407	470654	0.021247	LmjF.21	689598	710837	0.021239
LmjF.10	223000	338499	0.115499	LmjF.16	629639	648540	0.018901	LmjF.22	259699	270425	0.010726
LmjF.11	6500	259499	0.252999	LmjF.17	165801	190691	0.02489	LmjF.23	79625	110550	0.030925
LmjF.12	160000	553499	0.393499	LmjF.18	229071	248093	0.019022	LmjF.23	359871	380400	0.020529
LmjF.13	2500	288999	0.286499	LmjF.19	64863	90536	0.025673	LmjF.25	78531	120830	0.042299
LmjF.14	2000	261499	0.259499	LmjF.20	97349	131367	0.034018	LmjF.25	267594	280140	0.012546
LmjF.15	196500	450499	0.253999	LmjF.21	729773	741660	0.011887	LmjF.26	1018754	1041463	0.022709
LmjF.16	183500	375499	0.191999	LmjF.22	289990	330784	0.040794	LmjF.27	379452	390586	0.011134
LmjF.17	207500	457499	0.249999	LmjF.23	189540	220843	0.031303	LmjF.27	399940	420056	0.020116
LmjF.18	297500	577499	0.279999	LmjF.23	224812	240319	0.015507	LmjF.27	489742	510625	0.020883
LmjF.19	459500	645999	0.186499	LmjF.24	589685	624506	0.034821	LmjF.28	509883	520312	0.010429
LmjF.20	436500	654999	0.218499	LmjF.24	717586	730470	0.012884	LmjF.28	959567	980306	0.020739
LmjF.21	92000	377499	0.285499	LmjF.25	1	40874	0.040873	LmjF.29	889858	900309	0.010451
LmjF.22	515500	690499	0.174999	LmjF.25	409889	424211	0.014322	LmjF.29	909742	940383	0.030641
LmjF.23	507000	704499	0.197499	LmjF.25	819425	830058	0.010633	LmjF.29	1129805	1170151	0.040346
LmjF.24	329500	579499	0.249999	LmjF.26	229579	250424	0.020845	LmjF.30	359412	380465	0.021053
LmjF.25	455500	716999	0.261499	LmjF.27	69757	90494	0.020737	LmjF.30	480236	509994	0.029758
LmjF.26	482500	741499	0.258999	LmjF.27	716181	771049	0.054868	LmjF.30	1149606	1180986	0.03138
LmjF.27	828000	1067999	0.239999	LmjF.28	99605	130880	0.031275	LmjF.30	1269749	1290748	0.020999
LmjF.28	670000	940499	0.270499	LmjF.28	279718	310206	0.030488	LmjF.31	129541	170762	0.041221
LmjF.29	242500	505999	0.263499	LmjF.28	589957	600175	0.010218	LmjF.31	299844	320385	0.020541
LmjF.30	118500	349499	0.230999	LmjF.29	649398	680742	0.031344	LmjF.31	329474	401228	0.071754
LmjF.31	587500	893499	0.305999	LmjF.29	817871	830700	0.012829	LmjF.31	419679	440380	0.020701
LmjF.32	960500	1271499	0.310999	LmjF.29	1031703	1071003	0.0393	LmjF.31	919778	940458	0.02068
LmjF.33	705500	946999	0.241499	LmjF.30	629174	670755	0.041581	LmjF.31	1089637	1110259	0.020622
LmjF.34	175500	413999	0.238499	LmjF.30	784912	830936	0.046024	LmjF.31	1119663	1139963	0.0203
LmjF.35	426000	671499	0.245499	LmjF.30	1029787	1087453	0.057666	LmjF.31	1140011	1160311	0.0203
LmjF.36	975000	1254999	0.279999	LmjF.30	1199990	1227340	0.02735	LmjF.31	1169704	1210985	0.041281
N/A	N/A	N/A	N/A	LmjF.31	179883	206687	0.026804	LmjF.32	239665	260367	0.020702
N/A	N/A	N/A	N/A	LmjF.31	479849	500269	0.02042	LmjF.32	339669	360371	0.020702
N/A	N/A	N/A	N/A	LmjF.31	959865	977276	0.017411	LmjF.32	378679	422247	0.043568
N/A	N/A	N/A	N/A	LmjF.32	159208	201065	0.041857	LmjF.32	479078	521019	0.041941
N/A	N/A	N/A	N/A	LmjF.32	538047	550149	0.012102	LmjF.33	39797	60617	0.02082
N/A	N/A	N/A	N/A	LmjF.33	99915	110212	0.010297	LmjF.33	69863	90295	0.020432
N/A	N/A	N/A	N/A	LmjF.33	249993	270149	0.020156	LmjF.33	189820	210506	0.020686
N/A	N/A	N/A	N/A	LmjF.33	589907	610575	0.020668	LmjF.33	509738	550654	0.040916
N/A	N/A	N/A	N/A	LmjF.33	1329924	1340129	0.010205	LmjF.33	1029752	1070796	0.041044
N/A	N/A	N/A	N/A	LmjF.34	1	20111	0.02011	LmjF.33	1129995	1150211	0.020216
N/A	N/A	N/A	N/A	LmjF.34	459700	490697	0.030997	LmjF.33	1439754	1480998	0.041244
N/A	N/A	N/A	N/A	LmjF.34	562105	590283	0.028178	LmjF.34	830568	880069	0.049501
N/A	N/A	N/A	N/A	LmjF.34	1149842	1160335	0.010493	LmjF.34	969818	990481	0.020663
N/A	N/A	N/A	N/A	LmjF.35	39965	50201	0.010236	LmjF.34	1029629	1059957	0.030328
N/A	N/A	N/A	N/A	LmjF.35	379803	410556	0.030753	LmjF.34	1209947	1230206	0.020259
N/A	N/A	N/A	N/A	LmjF.35	736412	760224	0.023812	LmjF.34	1400011	1419918	0.019907
N/A	N/A	N/A	N/A	LmjF.35	849809	878133	0.028324	LmjF.34	1689633	1741070	0.051437
N/A	N/A	N/A	N/A	LmjF.35	1069741	1090563	0.020822	LmjF.35	1129820	1140295	0.010475
N/A	N/A	N/A	N/A	LmjF.35	1389865	1430478	0.040613	LmjF.35	1479933	1490080	0.010147
N/A	N/A	N/A	N/A	LmjF.35	1849815	1860080	0.010265	LmjF.35	1909865	1950614	0.040749
N/A	N/A	N/A	N/A	LmjF.36	154898	180521	0.025623	LmjF.36	299812	320494	0.020682
N/A	N/A	N/A	N/A	LmjF.36	489863	520391	0.030528	LmjF.36	339504	390587	0.051083
N/A	N/A	N/A	N/A	LmjF.36	778377	790237	0.01186	LmjF.36	429889	440344	0.010455
N/A	N/A	N/A	N/A	LmjF.36	1399960	1414421	0.014461	LmjF.36	559432	600068	0.040636
N/A	N/A	N/A	N/A	LmjF.36	1607146	1640287	0.033141	LmjF.36	929688	970405	0.040717
N/A	N/A	N/A	N/A	LmjF.36	1885797	1910222	0.024425	LmjF.36	1749684	1770277	0.020593
N/A	N/A	N/A	N/A	LmjF.36	2069876	2090721	0.020845	LmjF.36	2129904	2140435	0.010531
N/A	N/A	N/A	N/A	LmjF.36	2429926	2471035	0.041109	LmjF.36	2169714	2190429	0.020715
N/A	N/A	N/A	N/A	N/A	N/A	N/A	N/A	LmjF.36	2279872	2300661	0.020789
N/A	N/A	N/A	N/A	N/A	N/A	N/A	N/A	LmjF.36	2569709	2610479	0.04077

Supplementary Table 1
