# Supplementary material for: ATR, a DNA damage kinase, modulates DNA replication timing in Leishmania major
Source: PLoS Genet. 2025 Nov 24;21(11):e1011899. doi: 10.1371/journal.pgen.1011899 (PMC12677790; doi:10.1371/journal.pgen.1011899)
Supplement: S2 Table — (PDF) [file pgen.1011899.s011.pdf]

Table 1 – cell lines on this study

Name	*Path*	*Select Marker*	*Description*
**CC1**	**-**	**-**	Parental cell line
**Cas9T7**	***>CC1***	***Hyg***	Cell line expressing Cas9 endonuclease and T7 polimerase
***myc*ATR**	***>Cas9T7>CC1***	***Hyg, Pur***	Cell line expressing Cas9 endonuclease and T7 polimerase and mNG+3myc tagging at ATR's N'terminal
***3M*ATR**	***>Cas9T7>CC1***	***Hyg, Pur***	Cell line expressing Cas9 endonuclease and T7 polimerase and 3myc tagging at ATR's N'terminal
myc*ATR*ΔC+/-	***>mycATR>Cas9T7>CC1***	***Hyg, Pur, Neo***	Cell line expressing Cas9 endonuclease and T7 polimerase and mNG+3myc tagging at ATR's N'terminal and one allele deleted of ATR's C'terminal
myc*ATR*ΔC+/-	***>mycATR>Cas9T7>CC1***	***Hyg, Pur, Neo***	Cell line expressing Cas9 endonuclease and T7 polimerase and mNG+3myc tagging at ATR's N'terminal and both allele deleted of ATR's C'terminal

Supplementary Table 2
