# Supplementary material for: ATR, a DNA damage kinase, modulates DNA replication timing in Leishmania major
Source: PLoS Genet. 2025 Nov 24;21(11):e1011899. doi: 10.1371/journal.pgen.1011899 (PMC12677790; doi:10.1371/journal.pgen.1011899)
Supplement: S3 Table — (PDF) [file pgen.1011899.s012.pdf]

Name	Sequence	Description
a	TGTCACCTCTGTATTGGGCG	Fw upstream ATR gene
b	GCTGGTATTGCAGGAGGACA	Rv at ATR N'terminal gene
c	GCTGGACCGTTACATCTGGT	Fw at ATR gene
d	TACGGATGATGGCGCTACAC	Rv downstream ATR gene
e	GAAATTAATACGACTCACTATAGGATTGCTTCCCAGAGCAATGGGTTTTAGAGCTAGAAATAGC	5' sgRNA ATR tagging
f	GAAATTAATACGACTCACTATAGGCAAGAGCAGACGGAGAGCCTGTTTTAGAGCTAGAAATAGC	5'sgRNA ATR C'terminal deletion
g	GAAATTAATACGACTCACTATAGGGGACAAGCGCCTTGTCGTGCGTTTTAGAGCTAGAAATAGC	3'sgRNA ATR C'terminal deletion
h	TTTTTGGCTCGATCGCGGAGCTCCTAGCCGGTATAATGCAGACCTGCTGC	Fw Donor mycATR and 3MATR tagging
i	GAGGCCCTCGTCGTCAGTGACAGCTTCCATACTACCCGATCCTGATCCAG	Rv Donor mycATR tagging
j	GAGGCCCTCGTCGTCAGTGACAGCTTCCATAGAACCGGAACCGGAACCAC	Rv Donor 3MATR tagging
k	CAGCTGCGGTGGACGCTGCTGCGCAATCGCGTATAATGCAGACCTGCTGC	Fw Donor mycATRΔC deletion
m	TAATACCCACAGGAGACACCAGTCCCGCACCCAATTTGAGAGACCTGTGC	Rv Donor mycATRΔC deletion

Supplementary Table 3
