# Supplementary material for: ATR, a DNA damage kinase, modulates DNA replication timing in Leishmania major
Source: PLoS Genet. 2025 Nov 24;21(11):e1011899. doi: 10.1371/journal.pgen.1011899 (PMC12677790; doi:10.1371/journal.pgen.1011899)
Supplement: S4 Table — (PDF) [file pgen.1011899.s013.pdf]

ID	Sample	ID	Sample
		GL30	mycatr_nt_p10_rp1
		GL31	cl8_acute_p10_rp1
GL4	Cas9T7 (STA)	GL32	cl1_acute_p10_rp1
GL7	cl1_0h_acute_rp2	GL33	mycatr_chronic_5h_rp1
GL8	myatr_0h_acute_rp1	GL34	cl8_5h_chronic_rp1
GL9	cl1_0h_acute_rp1	GL35	cl8_5h_chronic_rp2
GL10	mycatr_0h_acute_rp2	GL36	cl1_5h_chronic_rp2
GL11	cl8_nt_rp1	GL37	cl1_chronic_5h_rp1
GL12	cl1_nt_rp1	GL38	mycatr_chronic_5h_rp2
GL13	cl1_nt_rp2	GL39	cl8_0h_chronic_rp2
GL14	mycatr_acute_p10_rp2	GL40	cl1_0h_chronic_rp1
GL15	mycatr_NT_rp2	GL41	cl8_rp1_0h_chronic
GL16	cl1_nt_p10_rp1	GL42	mycatr_chronic_0h_rp1
GL17	mycatr_acute_p10_rp1	GL43	mycatr_0h_chronic_rp2
GL18	cl1_chronic_p10_rp2	GL44	cl1_chronic_0h_rp2
GL19	cl8_nt_rp2	GL45	cl1_acute_p10_rp2
GL20	cl8_p10_chronic_rp1	GL46	cl8_acute_p10_rp2
GL21	cl8_nt_p10_rp2	GL47	cl8_0h_rp2_acute
GL22	mycatr_chronic_p10_rp1	GL48	cl8_acute_0h_rp1
GL23	mycatr_chronic_p10_rp2	GL49	cl1_acute_4h_rp1
GL24	mycatr_nt_rp1	GL50	cl8_acute_4h_rp1
GL25	cl1_nt_p10_rp2	GL51	mycatr_acute_4h_rp2
GL26	cl1_chronic_p10_rp1	GL52	cl1_acute_4h_rp2
GL27	mycatr_nt_p10_rp2	GL53	cl8_acute_4h_rp2
GL28	cl8_chronic_p10_rp2	GL54	mycatr_acute_4h_rp1
GL29	cl8_nt_p10_rp1		

Supplementary Table 4
