# Supplementary material for: ATR, a DNA damage kinase, modulates DNA replication timing in Leishmania major
Source: PLoS Genet. 2025 Nov 24;21(11):e1011899. doi: 10.1371/journal.pgen.1011899 (PMC12677790; doi:10.1371/journal.pgen.1011899)
Supplement: S1 File — (PDF) [file pgen.1011899.s014.pdf]

# Supporting information:
# Supplemental Dataset – Da Silva et al.

Western blots and raw data from this study.
(labels are shown in corresponding figures in the manuscript

## Figure 1C
**R2**

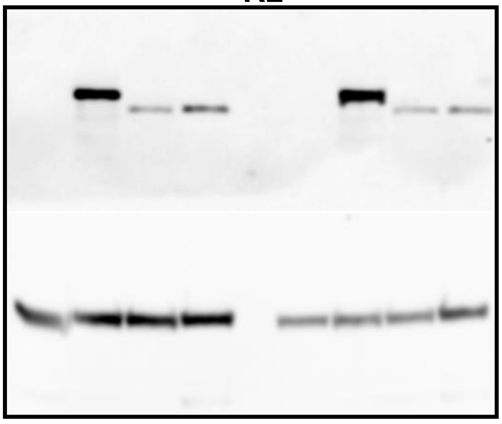

| | $^{myc}ATR$ | | $^{myc}ATR^{\Delta C-/-}$ (Cl1) | | $^{myc}ATR^{\Delta C-/-}$ (Cl8) | |
|---|---|---|---|---|---|---|
| | R1 | R2 | R1 | R2 | R1 | R2 |
| Relative Values | 1 | 1 | 0.11847 | 0.085449 | 0.290347 | 0.093314 |
| SD | | 0 | | 0.01651 | | 0.098517 |

## Figure 1D – Growth Curve

| Days | $^{myc}ATR$ | | $^{myc}ATR^{\Delta C +/-}$ | | $^{myc}ATR^{\Delta C -/-}$ (Cl1) | | $^{myc}ATR^{\Delta C -/-}$ (Cl8) | |
|---|---|---|---|---|---|---|---|---|
| | R1 | R2 | R1 | R2 | R1 | R2 | R1 | R2 |
| 0 | 200000 | 200000 | 200000 | 200000 | 200000 | 200000 | 200000 | 200000 |
| 1 | 1400000 | 2000000 | 1300000 | 1500000 | 1000000 | 500000 | 790000 | 760000 |
| 2 | 17000000 | 18000000 | 19000000 | 18000000 | 5000000 | 7300000 | 5800000 | 6000000 |
| 3 | 25000000 | 23000000 | 27000000 | 26000000 | 22000000 | 21000000 | 21000000 | 21000000 |
| 4 | 22000000 | 23000000 | 30000000 | 31000000 | 16000000 | 18000000 | 17000000 | 19000000 |
| 5 | 23000000 | 21000000 | 29000000 | 35000000 | 27000000 | 31000000 | 22000000 | 26000000 |

## Figure 1D – Doubling Time

| $^{myc}ATR$ | | | $^{myc}ATR^{\Delta C +/-}$ | | | $^{myc}ATR^{\Delta C -/-}$ (Cl1) | | | $^{myc}ATR^{\Delta C -/-}$ (Cl8) | | |
|---|---|---|---|---|---|---|---|---|---|---|---|
| | R1 | R2 | | R1 | R2 | | R1 | R2 | | R1 | R2 |
| Hours | 7 | 7 | | 7 | 9 | | 9 | 14 | | 10 | 11 |

## Figure 1E – Resistance Curve

| Concentration of HU (mM) | $^{myc}ATR$ | | $^{myc}ATR^{\Delta C +/-}$ | | $^{myc}ATR^{\Delta C -/-}$ (Cl1) | | $^{myc}ATR^{\Delta C -/-}$ (Cl8) | |
|---|---|---|---|---|---|---|---|---|
| | R1 | R2 | R1 | R2 | R1 | R2 | R1 | R2 |
| 0 | 100 | 100 | 100 | 100 | 100 | 100 | 100 | 100 |
| 0.1 | 88 | 91 | 83 | 100 | 55 | 58 | 35 | 63 |
| 0.2 | 84 | 74 | 78 | 90 | 23 | 38 | 13 | 21 |
| 0.3 | 28 | 43 | 30 | 44 | 9 | 8 | 8 | 11 |
| 0.4 | 13 | 14 | 7 | 12 | 5 | 4 | 4 | 6 |
| 0.5 | 12 | 14 | 3 | 4 | 2 | 2 | 1 | 4 |

## Figure 2B – Nuclear localisation

| | Cas9T7 | | $^{myc}$ATR | | $^{myc}$ATR$^{\Delta C\ -/-}$ (Cl1) | | $^{myc}$ATR$^{\Delta C\ -/-}$ (Cl8) | |
|---|---|---|---|---|---|---|---|---|
| | R1 | R2 | R1 | R2 | R1 | R2 | R1 | R2 |
| mean | 1164.666 | 3004.418 | 15012.46 | 9418.617 | 5373.077 | 4982.913 | 5093.335 | 5239.918 |
| SD | 449.635 | 495.7376 | 4838.833 | 3426.484 | 2368.918 | 1289.392 | 2752.145 | 1934.208 |

## Figure 3B –ssDNA accumulation

| | $^{myc}$ATR (NT) | | $^{myc}$ATR (HU) | | $^{myc}$ATR$^{\Delta C}$Cl1 (NT) | | $^{myc}$ATR$^{\Delta C}$Cl1 (HU) | | $^{myc}$ATR$^{\Delta C}$Cl8 (NT) | | $^{myc}$ATR$^{\Delta C}$Cl8 (HU) | | Cas9T7 | |
|---|---|---|---|---|---|---|---|---|---|---|---|---|---|---|
| | R1 | R2 | R1 | R2 | R1 | R2 | R1 | R2 | R1 | R2 | R1 | R2 | R1 | R2 |
| mean | 4877.278 | 1924.013 | 3709.579 | 2164.043 | 4638.818 | 2638.698 | 10053.18 | 4381.946 | 13215.88 | 3374.022 | 12767.54 | 5059.571 | 2166.534 | 1594.734 |
| SD | 1461.758 | 421.4342 | 1038.062 | 735.6938 | 1383.525 | 780.7349 | 6964.141 | 2874.491 | 3988.925 | 964.4868 | 8730.972 | 3605.211 | 508.1296 | 278.2265 |

## Figure 3C –Cell cycle profile

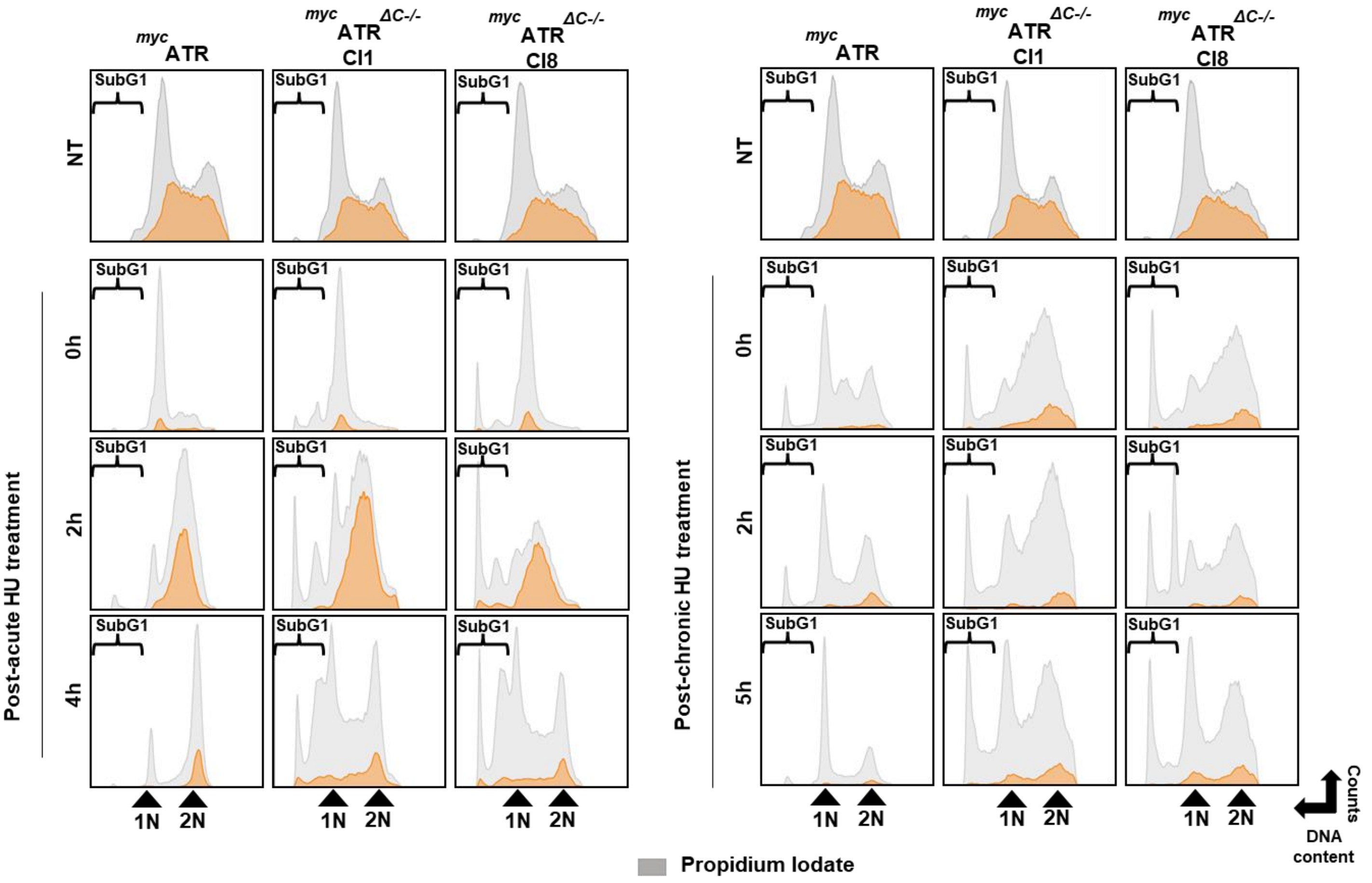


## Figure 3E – EdU positive cells

| | $^{myc}$ATR | | $^{myc}$ATR$^{\Delta C\ -/-}$ (Cl1) | | $^{myc}$ATR$^{\Delta C\ -/-}$ (Cl8) | | $^{myc}$ATR | | $^{myc}$ATR$^{\Delta C\ -/-}$ (Cl1) | | $^{myc}$ATR$^{\Delta C\ -/-}$ (Cl8) | | $^{myc}$ATR | | $^{myc}$ATR$^{\Delta C\ -/-}$ (Cl1) | | $^{myc}$ATR$^{\Delta C\ -/-}$ (Cl8) | |
|---|---|---|---|---|---|---|---|---|---|---|---|---|---|---|---|---|---|---|
| | R1 | R2 | R1 | R2 | R1 | R2 | R1 | R2 | R1 | R2 | R1 | R2 | R1 | R2 | R1 | R2 | R1 | R2 |
| percentage | 34 | 38 | 34 | 28 | 39 | 41 | 34 | 28 | 4 | 2 | 4 | 5 | 30 | 30 | 9 | 22 | 11 | 14 |
| mean | | 36 | | 31 | | 30 | | 14 | | 3 | | 4.5 | | 40 | | 15.5 | | 12.5 |
| SD | | 2 | | 3 | | 0 | | 3 | | 1 | | 0.5 | | 1 | | 6.5 | | 1.5 |

Figure 4A
R2

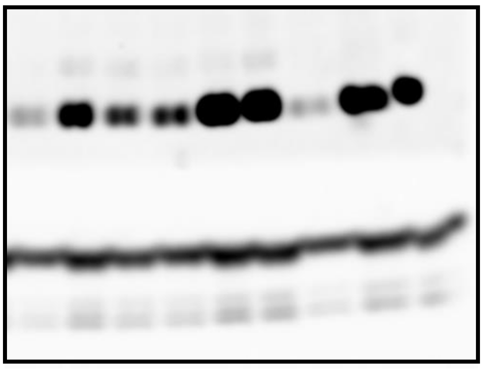

Figure 4B
R2

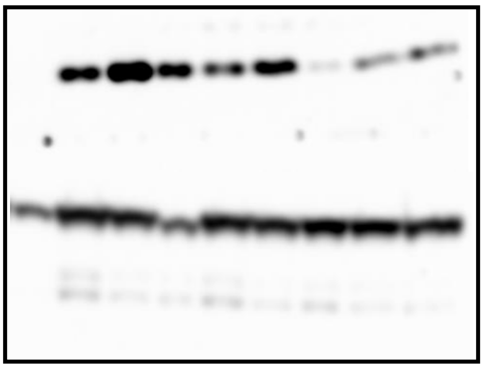

Figure 4A
R3

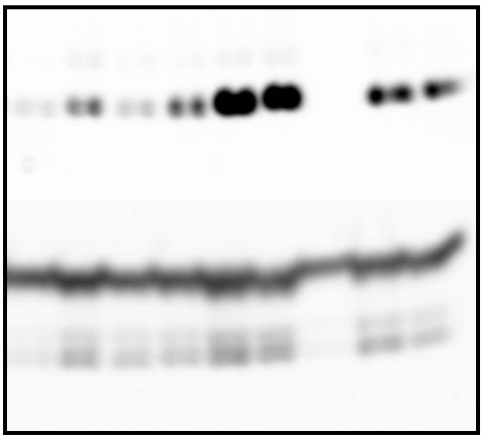

Figure 4B
R3

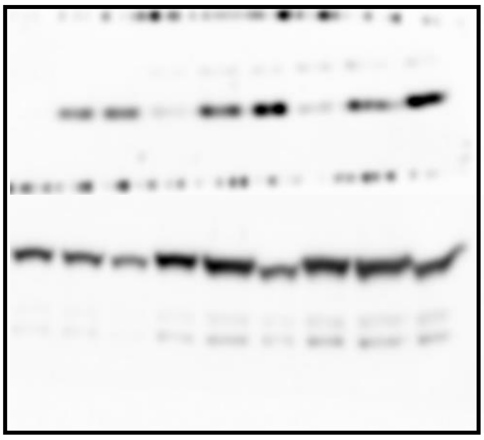

## Figure 4C – WB post-HU chronic

| | $^{myc}$ATR | | | | | $^{myc}$ATR$^{\Delta C\ -/-}$ (Cl1) | | | | | $^{myc}$ATR$^{\Delta C\ -/-}$ (Cl8) | | | | |
|---|---|---|---|---|---|---|---|---|---|---|---|---|---|---|---|
| | R1 | R2 | R3 | Mean | SD | R1 | R2 | R3 | Mean | SD | R1 | R2 | R3 | Mean | SD |
| NT | 1 | 1 | 1 | 1 | 0 | 32 | 1 | 5 | 5 | 14 | 1 | 1 | 1 | 1 | 0 |
| 0h | 2 | 8 | 35 | 8 | 14 | 85 | 51 | 71 | 68 | 14 | 32 | 58 | 43 | 43 | 11 |
| 5h | 4 | 4 | 23 | 7 | 9 | 77 | 65 | 62 | 68 | 6 | 38 | 75 | 57 | 55 | 15 |

## Figure 4D - WB post-HU acute

| | $^{myc}$ATR | | | | | $^{myc}$ATR$^{\Delta C\ -/-}$ (Cl1) | | | | | $^{myc}$ATR$^{\Delta C\ -/-}$ (Cl8) | | | | |
|---|---|---|---|---|---|---|---|---|---|---|---|---|---|---|---|
| | R1 | R2 | R3 | Mean | SD | R1 | R2 | R3 | Mean | SD | R1 | R2 | R3 | Mean | SD |
| NT | 1 | 1 | 5 | 2 | 2 | 6 | 13 | 3 | 6 | 4 | 7 | 3 | 6 | 5 | 2 |
| 0h | 28 | 56 | 58 | 45 | 14 | 18 | 49 | 36 | 32 | 13 | 19 | 20 | 20 | 20 | 0 |
| 5h | 45 | 95 | 94 | 74 | 23 | 34 | 66 | 87 | 58 | 22 | 26 | 44 | 55 | 40 | 12 |

# Figure 4F – percentage of aberrant cells

| | | 1N1K | | | | 1N2K | | | | 2N2K | | | |
|---|---|---|---|---|---|---|---|---|---|---|---|---|---|
| | | R1 | R2 | Mean | SD | R1 | R2 | Mean | SD | R1 | R2 | Mean | SD |
| NT | $^{myc}$ATR | 93.25 | 87.3 | 90.275 | 2.975 | 1.31 | 4.1 | 2.705 | 1.395 | 4.36 | 5.6 | 4.98 | 0.62 |
| NT | $^{myc}$ATR$^{\Delta C}$Cl1 | 91.47 | 86.9 | 89.185 | 2.285 | 0.92 | 2.3 | 1.61 | 0.69 | 3.69 | 4.6 | 4.145 | 0.455 |
| NT | $^{myc}$ATR$^{\Delta C}$Cl8 | 78.48 | 82.7 | 80.59 | 2.11 | 3.14 | 2.7 | 2.92 | 0.22 | 5.38 | 7.3 | 6.34 | 0.96 |
| Chronic | $^{myc}$ATR | 86.12 | 88.73 | 87.425 | 1.305 | 1.74 | 1.69 | 1.715 | 0.025 | 5.21 | 1.97 | 3.59 | 1.62 |
| Chronic | $^{myc}$ATR$^{\Delta C}$Cl1 | 74.72 | 68.26 | 71.49 | 3.23 | 3.64 | 2.4 | 3.02 | 0.62 | 3.19 | 1.8 | 2.495 | 0.695 |
| Chronic | $^{myc}$ATR$^{\Delta C}$Cl8 | 71.6 | 69.01 | 70.305 | 1.295 | 7.65 | 2.82 | 5.235 | 2.415 | 2.22 | 1.41 | 1.815 | 0.405 |
| Acute | $^{myc}$ATR | 97.32 | 92.26 | 94.79 | 2.53 | 0.49 | 0 | 0.245 | 0.245 | 0.49 | 0 | 0.245 | 0.245 |
| Acute | $^{myc}$ATR$^{\Delta C}$Cl1 | 75.85 | 70 | 72.925 | 2.925 | 3.94 | 2.94 | 3.44 | 0.5 | 6.56 | 4.71 | 5.635 | 0.925 |
| Acute | $^{myc}$ATR$^{\Delta C}$Cl8 | 67.89 | 64.06 | 65.975 | 1.915 | 3.94 | 3.91 | 3.925 | 0.015 | 8.73 | 6.25 | 7.49 | 1.24 |

| 2N1K | | | | 0N1K | | | | 1N0K | | | |
|---|---|---|---|---|---|---|---|---|---|---|---|
| R1 | R2 | Mean | SD | R1 | R2 | Mean | SD | R1 | R2 | Mean | SD |
| 0.44 | 2.6 | 1.52 | 1.08 | 0.65 | 0.4 | 0.525 | 0.125 | 0 | 0 | 0 | 0 |
| 2.07 | 2.9 | 2.485 | 0.415 | 0.46 | 0 | 0.23 | 0.23 | 0.69 | 0.4 | 0.545 | 0.145 |
| 2.47 | 4.7 | 3.585 | 1.115 | 9.19 | 2 | 5.595 | 3.595 | 0.22 | 0 | 0.11 | 0.11 |
| 3.47 | 5.07 | 4.27 | 0.8 | 1.95 | 0.28 | 1.115 | 0.835 | 0.65 | 1.41 | 1.03 | 0.38 |
| 5.69 | 3.59 | 4.64 | 1.05 | 9.79 | 10.78 | 10.285 | 0.495 | 1.14 | 2.4 | 1.77 | 0.63 |
| 4.69 | 6.34 | 5.515 | 0.825 | 11.36 | 10.56 | 10.96 | 0.4 | 1.98 | 6.34 | 4.16 | 2.18 |
| 0.24 | 1.19 | 0.715 | 0.475 | 1.22 | 0 | 0.61 | 0.61 | 0 | 6.55 | 3.275 | 3.275 |
| 2.62 | 2.35 | 2.485 | 0.135 | 9.97 | 7.06 | 8.515 | 1.455 | 0.26 | 3.53 | 1.895 | 1.635 |
| 1.97 | 4.69 | 3.33 | 1.36 | 12.11 | 10.94 | 11.525 | 0.585 | 1.13 | 0 | 0.565 | 0.565 |

| Others | | | | 1M | | | |
|---|---|---|---|---|---|---|---|
| R1 | R2 | Mean | SD | R1 | R2 | Mean | SD |
| 0 | 0 | 0 | 0 | 0 | 0 | 0 | 0 |
| 0.23 | 0 | 0.115 | 0.115 | 0.46 | 0.9 | 0.68 | 0.22 |
| 0 | 0 | 0 | 0 | 1.12 | 0.2 | 0.66 | 0.46 |
| 0 | 0.28 | 0.14 | 0.14 | 0.87 | 0.56 | 0.715 | 0.155 |
| 0.46 | 0 | 0.23 | 0.23 | 1.37 | 10.78 | 6.075 | 4.705 |
| 0 | 0 | 0 | 0 | 0.49 | 3.52 | 2.005 | 1.515 |
| 0 | 0 | 0 | 0 | 0.24 | 0 | 0.12 | 0.12 |
| 0 | 0 | 0 | 0 | 0.79 | 9.41 | 5.1 | 4.31 |
| 0 | 0.78 | 0.39 | 0.39 | 4.23 | 9.38 | 6.805 | 2.575 |

## Figure 5C replication programme NT

| Chromosome Length | $^{myc}$ATR | $^{myc}$ATR$^{\Delta C\ -/-}$ (Cl1) | $^{myc}$ATR$^{\Delta C\ -/-}$ (Cl8) |
|---|---|---|---|
| 0.355712 | 0.045753 | -0.06849 | -0.07001 |
| 0.384502 | 0.197911 | 0.193601 | 0.147083 |
| 0.472852 | 0.179271 | 0.123747 | 0.126746 |
| 0.465823 | 0.030134 | 0.015057 | -0.01184 |
| 0.516869 | 0.227368 | 0.172388 | 0.101727 |
| 0.596352 | 0.238565 | 0.098053 | 0.069969 |
| 0.57496 | -0.1506 | -0.6771 | -0.56043 |
| 0.573434 | 0.21402 | 0.228491 | 0.195514 |
| 0.570865 | 0.295283 | 0.278755 | 0.259183 |
| 0.582573 | 0.01816 | -0.01703 | -0.01188 |
| 0.675346 | 0.334501 | -0.02782 | -0.24429 |
| 0.654595 | 0.224373 | 0.213602 | 0.19741 |
| 0.622644 | 0.246672 | 0.085326 | 0.152496 |
| 0.629517 | 0.220515 | 0.327517 | 0.258801 |
| 0.714651 | 0.202859 | 0.070142 | 0.072529 |
| 0.684829 | 0.206149 | 0.079167 | 0.100813 |
| 0.739748 | 0.017792 | 0.013362 | 0.023184 |
| 0.702208 | -0.04998 | -0.00541 | 0.038759 |
| 0.742537 | 0.200892 | 0.239143 | 0.215377 |
| 0.772972 | 0.162512 | 0.143333 | 0.106016 |
| 0.716602 | 0.039216 | 0.132223 | 0.133429 |
| 0.772565 | 0.203839 | -0.04846 | -0.0537 |
| 0.84095 | 0.197878 | 0.205273 | 0.174545 |
| 0.912845 | 0.117786 | 0.099548 | 0.172405 |
| 1.09154 | 0.106266 | 0.183211 | 0.185881 |
| 1.130424 | -0.15238 | -0.11086 | -0.06887 |
| 1.160104 | 0.049797 | 0.119168 | 0.104452 |
| 1.212663 | -0.04899 | -0.00727 | 0.015004 |
| 1.403434 | 0.016414 | 0.141002 | 0.092978 |
| 1.484328 | -0.4757 | -0.8505 | -0.85208 |
| 1.604637 | -0.15876 | -0.1045 | -0.0963 |
| 1.583653 | -0.04865 | 0.011253 | 0.055847 |
| 1.866748 | -0.09784 | 0.007131 | 0.015158 |
| 2.090474 | -0.10354 | 0.026762 | 0.027464 |
| 2.682151 | -0.18449 | -0.01371 | 0.0000134 |

## Figure 5D - Average MFAseq signal

| $^{myc}$ATR | | | | | | $^{myc}$ATR$^{\Delta C}$ $^{-/-}$ (Cl1) | | | | | |
|---|---|---|---|---|---|---|---|---|---|---|---|
| Smaller | | Medium | | Larger | | Smaller | | Medium | | Larger | |
| R1 | R2 | R1 | R2 | R1 | R2 | R1 | R2 | R1 | R2 | R1 | R2 |
| | | 0.074892 | 0.334501 | 0.017307 | 0.117786 | | | -0.02782 | -0.0877 | 0.099548 | 0.092593 |
| -0.10431 | 0.045753 | 0.02668 | 0.224373 | -0.06749 | 0.106266 | -0.06849 | -0.05772 | 0.213602 | 0.188676 | 0.183211 | 0.158471 |
| -0.0467 | 0.197911 | -0.00585 | 0.220515 | -0.13983 | -0.15238 | 0.193601 | 0.209724 | 0.327517 | 0.215969 | -0.11086 | -0.09965 |
| 0.169543 | 0.179271 | -0.02106 | 0.202859 | -0.02717 | 0.049797 | 0.123747 | 0.110214 | 0.070142 | 0.06372 | 0.119168 | 0.129497 |
| 0.155519 | 0.030134 | 0.076941 | 0.206149 | 0.042351 | -0.04899 | 0.015057 | -0.03566 | 0.079167 | 0.08317 | -0.00727 | 0.006311 |
| -0.09373 | 0.227368 | 0.23302 | 0.017792 | -0.06428 | 0.016414 | 0.172388 | 0.154691 | 0.013362 | 0.048189 | 0.141002 | 0.122681 |
| 0.050818 | 0.238565 | 0.104584 | -0.04998 | -0.04503 | -0.4757 | 0.098053 | 0.095916 | -0.00541 | 0.034872 | -0.8505 | -0.85498 |
| -0.04001 | -0.1506 | 0.094504 | 0.200892 | -0.07581 | -0.15876 | -0.6771 | -0.6807 | 0.239143 | 0.23189 | -0.1045 | -0.09764 |
| 0.117594 | 0.21402 | 0.045439 | 0.162512 | -0.13902 | -0.04865 | 0.228491 | 0.229382 | 0.143333 | 0.140984 | 0.011253 | 0.014679 |
| 0.279495 | 0.295283 | 0.158862 | 0.039216 | -0.05603 | -0.09784 | 0.278755 | 0.247887 | 0.132223 | 0.168119 | 0.007131 | 0.025719 |
| 0.082451 | 0.01816 | 0.091087 | 0.203839 | -0.07617 | -0.10354 | -0.01703 | 0.021387 | -0.04846 | -0.06141 | 0.026762 | 0.050493 |
| 0.251876 | 0.246672 | -0.00068 | 0.197878 | -0.08569 | -0.18449 | 0.085326 | 0.051824 | 0.205273 | 0.180349 | -0.01371 | 0.00726 |

| $^{myc}$ATR$^{\Delta C}$ $^{-/-}$ (Cl8) | | | | | |
|---|---|---|---|---|---|
| Smaller | | Medium | | Larger | |
| R1 | R2 | R1 | R2 | R1 | R2 |
| | | -0.26537 | -0.24429 | 0.03264 | 0.172405 |
| -0.18404 | -0.07001 | 0.031249 | 0.19741 | 0.010354 | 0.185881 |
| -0.11868 | 0.147083 | 0.011739 | 0.258801 | -0.10296 | -0.06887 |
| 0.120933 | 0.126746 | -0.06819 | 0.072529 | 0.026079 | 0.104452 |
| 0.15597 | -0.01184 | 0.008919 | 0.100813 | 0.087457 | 0.015004 |
| -0.13668 | 0.101727 | 0.200164 | 0.023184 | 0.017307 | 0.092978 |
| -0.05216 | 0.069969 | 0.109051 | 0.038759 | -0.28788 | -0.85208 |
| -0.27023 | -0.56043 | 0.069008 | 0.215377 | -0.04269 | -0.0963 |
| 0.098922 | 0.195514 | 0.039308 | 0.106016 | -0.08136 | 0.055847 |
| 0.235908 | 0.259183 | 0.196844 | 0.133429 | 0.015588 | 0.015158 |
| 0.044893 | -0.01188 | -0.03311 | -0.0537 | -0.01271 | 0.027464 |
| 0.168552 | 0.152496 | 0.010083 | 0.174545 | 0.04044 | 1.34E-05 |

## Figure 5E - Number of Chromosomes

| | | Early | | Late | |
|---|---|---|---|---|---|
| $^{myc}$ATR | Smaller | 7 | 10 | 4 | 1 |
| | Medium | 9 | 11 | 3 | 1 |
| | Larger | 2 | 4 | 10 | 8 |
| $^{myc}$ATR$^{\Delta C}$ $^{-/-}$ (Cl1) | Smaller | 8 | 8 | 3 | 3 |
| | Medium | 8 | 9 | 4 | 3 |
| | Larger | 7 | 9 | 5 | 3 |
| $^{myc}$ATR$^{\Delta C}$ $^{-/-}$ (Cl8) | Smaller | 6 | 7 | 5 | 4 |
| | Medium | 9 | 10 | 3 | 2 |
| | Larger | 7 | 9 | 5 | 3 |

## Figure 5F - Replication Timing Chronic

| | 0h | 0h | 0h | 5h | 5h | 5h |
|---|---|---|---|---|---|---|
| Chromosome Length | $^{myc}$ATR | $^{myc}ATR^{\Delta C -/-}$ (Cl1) | $^{myc}ATR^{\Delta C -/-}$ (Cl8) | $^{myc}$ATR | $^{myc}ATR^{\Delta C -/-}$ (Cl1) | $^{myc}ATR^{\Delta C -/-}$ (Cl8) |
| 0.355712 | 0.411385 | -0.18792 | -0.2091 | -0.25888 | -0.31226 | -0.28901 |
| 0.384502 | 0.496223 | -0.15195 | -0.17614 | -0.22352 | -0.30001 | -0.29647 |
| 0.472852 | 0.366174 | 0.146746 | 0.127106 | 0.05652 | 0.045317 | 0.042607 |
| 0.465823 | 0.366992 | 0.192135 | 0.215928 | 0.037966 | 0.050423 | 0.119069 |
| 0.516869 | 0.47922 | -0.14435 | -0.14328 | -0.22998 | -0.22422 | -0.24327 |
| 0.596352 | 0.433283 | -0.01727 | -0.02875 | -0.05056 | -0.10409 | -0.10148 |
| 0.57496 | 0.092144 | -0.79744 | -0.65756 | -0.02018 | -0.83319 | -0.73095 |
| 0.573434 | 0.374161 | 0.087199 | 0.08213 | 0.032322 | -0.02663 | 0.019158 |
| 0.570865 | 0.273812 | 0.168911 | 0.168345 | 0.138729 | 0.141954 | 0.189671 |
| 0.582573 | 0.112938 | 0.031092 | 0.036216 | -0.0088 | -0.00389 | -0.00958 |
| 0.675346 | 0.115775 | -0.43931 | -0.34656 | 0.003385 | -0.45936 | -0.40778 |
| 0.654595 | 0.302073 | 0.022711 | 0.015427 | -0.03773 | -0.05539 | -0.04283 |
| 0.622644 | 0.122011 | 0.055227 | 0.071988 | 0.206615 | -0.02702 | 0.035806 |
| 0.629517 | 0.352317 | -0.04489 | -0.05401 | -0.07689 | -0.11199 | -0.08492 |
| 0.714651 | 0.358763 | -0.10755 | -0.10903 | -0.08403 | -0.17824 | -0.12424 |
| 0.684829 | 0.248226 | 0.028674 | 0.030515 | -0.05015 | -0.01899 | -0.03224 |
| 0.739748 | -0.09944 | 0.101464 | 0.070154 | 0.188314 | 0.106589 | 0.026654 |
| 0.702208 | -0.09999 | 0.148096 | 0.132037 | 0.105 | 0.134584 | 0.112298 |
| 0.742537 | 0.21271 | 0.106725 | 0.08763 | -0.00718 | 0.058817 | 0.062891 |
| 0.772972 | 0.225261 | 0.022359 | 0.004434 | -0.01445 | -0.03992 | -0.04958 |
| 0.716602 | 0.079128 | 0.199789 | 0.196598 | 0.145746 | 0.158324 | 0.171361 |
| 0.772565 | 0.135245 | -0.09907 | -0.04505 | 0.049326 | -0.11141 | -0.03985 |
| 0.84095 | 0.290631 | 0.011755 | 0.03508 | -0.00322 | -0.01775 | -0.00125 |
| 0.912845 | 0.145821 | 0.034615 | 0.003344 | 0.01251 | 0.00162 | 0.012752 |
| 1.09154 | 0.119389 | 0.016972 | 0.009188 | -0.01474 | 0.013945 | 0.031912 |
| 1.130424 | -0.12181 | -0.06314 | -0.06253 | -0.13642 | -0.08919 | -0.08681 |
| 1.160104 | -0.04783 | -0.01755 | -0.03344 | -0.02061 | -0.00317 | -0.01226 |
| 1.212663 | -0.21013 | 0.073669 | 0.053347 | 0.043119 | 0.109085 | 0.081427 |
| 1.403434 | -0.06004 | 0.009733 | 0.004868 | -0.01025 | 0.053705 | 0.054492 |
| 1.484328 | -0.2741 | -0.22714 | -0.20459 | 0.045621 | -0.12214 | -0.11086 |
| 1.604637 | -0.29094 | -0.04132 | -0.03894 | -0.04367 | 0.020415 | -0.00387 |
| 1.583653 | -0.23264 | -0.01174 | -0.01974 | -0.07251 | 0.068925 | 0.019801 |
| 1.866748 | -0.29222 | 0.050479 | 0.03382 | 0.0157 | 0.113276 | 0.090116 |
| 2.090474 | -0.23255 | 0.094949 | 0.108737 | 0.010505 | 0.164672 | 0.114285 |
| 2.682151 | -0.2987 | 0.077104 | 0.067072 | 0.027247 | 0.143726 | 0.135355 |

# Figure 7B - SNPs

| 1- p10/NT | | 2- p10/NT | | 1- p10/NT | | 2- p10/NT | | 1- p10/NT | | 2- p10/NT | |
|---|---|---|---|---|---|---|---|---|---|---|---|
| R1 | R2 | R1 | R2 | R1 | R2 | R1 | R2 | R1 | R2 | R1 | R2 |
| 1.483271 | 0.420074 | 0.483271 | 0.921933 | 0.527881 | 0.684015 | 0.565056 | 0.431227 | 0.472119 | 0.69145 | 0.520446 | 0.412639 |
| 0.966292 | 0.297753 | 0.441011 | 0.77809 | 0.30618 | 0.460674 | 0.396067 | 0.292135 | 0.269663 | 0.530899 | 0.328652 | 0.311798 |
| 1.348052 | 0.32987 | 0.571429 | 0.94026 | 0.480519 | 0.522078 | 0.52987 | 0.363636 | 0.353247 | 0.633766 | 0.444156 | 0.34026 |
| 1.289641 | 0.359408 | 0.515856 | 0.942918 | 0.399577 | 0.4926 | 0.501057 | 0.369979 | 0.378436 | 0.632135 | 0.427061 | 0.323467 |
| 1.208155 | 0.33691 | 0.534335 | 0.815451 | 0.448498 | 0.521459 | 0.570815 | 0.330472 | 0.285408 | 0.67382 | 0.345494 | 0.343348 |
| 0.858801 | 0.222437 | 0.353965 | 0.740812 | 0.220503 | 0.357834 | 0.352031 | 0.266925 | 0.226306 | 0.54352 | 0.216634 | 0.255319 |
| 0.954774 | 0.274707 | 0.447236 | 0.795645 | 0.333333 | 0.512563 | 0.463987 | 0.314908 | 0.293132 | 0.544389 | 0.303183 | 0.308208 |
| 0.532174 | 0.245217 | 0.234783 | 0.368696 | 0.276522 | 0.342609 | 0.33913 | 0.276522 | 0.250435 | 0.318261 | 0.226087 | 0.210435 |
| 1.134146 | 0.247387 | 0.489547 | 0.87108 | 0.391986 | 0.425087 | 0.391986 | 0.308362 | 0.261324 | 0.56446 | 0.327526 | 0.30662 |
| 1.162872 | 0.255692 | 0.462347 | 0.74606 | 0.359019 | 0.472855 | 0.448336 | 0.297723 | 0.325744 | 0.525394 | 0.32049 | 0.292469 |
| 0.881647 | 0.19211 | 0.29331 | 0.694683 | 0.233276 | 0.389365 | 0.319039 | 0.236707 | 0.238422 | 0.413379 | 0.267581 | 0.238422 |
| 0.745562 | 0.45858 | 0.323964 | 0.452663 | 0.303254 | 0.548817 | 0.523669 | 0.430473 | 0.399408 | 0.489645 | 0.427515 | 0.328402 |
| 1.090076 | 0.264122 | 0.418321 | 0.81374 | 0.280916 | 0.412214 | 0.39084 | 0.253435 | 0.238168 | 0.517557 | 0.280916 | 0.264122 |
| 0.775281 | 0.300161 | 0.330658 | 0.452648 | 0.29374 | 0.377207 | 0.335474 | 0.295345 | 0.325843 | 0.378812 | 0.375602 | 0.268058 |
| 0.920635 | 0.233333 | 0.37619 | 0.81746 | 0.280952 | 0.415873 | 0.371429 | 0.287302 | 0.25873 | 0.460317 | 0.333333 | 0.244444 |
| 0.848951 | 0.239161 | 0.386014 | 0.696503 | 0.286713 | 0.39021 | 0.33986 | 0.300699 | 0.258741 | 0.490909 | 0.293706 | 0.261538 |
| 0.908029 | 0.181022 | 0.348905 | 0.658394 | 0.286131 | 0.385401 | 0.340146 | 0.236496 | 0.240876 | 0.445255 | 0.271533 | 0.226277 |
| 0.975676 | 0.235135 | 0.372973 | 0.741892 | 0.283784 | 0.397297 | 0.335135 | 0.255405 | 0.224324 | 0.512162 | 0.290541 | 0.251351 |
| 1.099573 | 0.227596 | 0.371266 | 0.722617 | 0.312945 | 0.429587 | 0.392603 | 0.305832 | 0.288762 | 0.465149 | 0.29303 | 0.261735 |
| 0.916555 | 0.304172 | 0.386272 | 0.741588 | 0.253028 | 0.387618 | 0.348587 | 0.277254 | 0.235532 | 0.442799 | 0.300135 | 0.275908 |
| 1.019405 | 0.287193 | 0.441138 | 0.833118 | 0.300129 | 0.465718 | 0.407503 | 0.284605 | 0.327296 | 0.558862 | 0.322122 | 0.258732 |
| 1.090656 | 0.276151 | 0.444909 | 0.882845 | 0.297071 | 0.443515 | 0.426778 | 0.295676 | 0.309623 | 0.511855 | 0.320781 | 0.295676 |
| 0.535576 | 0.160414 | 0.216041 | 0.353169 | 0.163001 | 0.288486 | 0.227684 | 0.168176 | 0.164295 | 0.265201 | 0.1837 | 0.168176 |
| 1.041617 | 0.247325 | 0.423306 | 0.776457 | 0.310345 | 0.470868 | 0.395957 | 0.290131 | 0.282996 | 0.581451 | 0.297265 | 0.296076 |
| 0.932092 | 0.202629 | 0.382256 | 0.821468 | 0.259584 | 0.392114 | 0.342826 | 0.258488 | 0.234392 | 0.474261 | 0.273823 | 0.217963 |
| 0.818681 | 0.217033 | 0.349817 | 0.742674 | 0.261905 | 0.351648 | 0.332418 | 0.25641 | 0.263736 | 0.434066 | 0.277473 | 0.222527 |
| 0.825818 | 0.167109 | 0.306808 | 0.68435 | 0.208665 | 0.307692 | 0.281167 | 0.183908 | 0.188329 | 0.3855 | 0.236958 | 0.198055 |
| 0.871662 | 0.216193 | 0.378122 | 0.79242 | 0.26615 | 0.38329 | 0.360034 | 0.26615 | 0.250646 | 0.486649 | 0.268734 | 0.237726 |
| 0.883759 | 0.173949 | 0.349547 | 0.72465 | 0.216818 | 0.347073 | 0.292663 | 0.222589 | 0.20033 | 0.395713 | 0.229184 | 0.206101 |
| 0.745726 | 0.135328 | 0.291311 | 0.691595 | 0.16453 | 0.269231 | 0.247151 | 0.161681 | 0.152422 | 0.376781 | 0.183048 | 0.166667 |
| 0.425589 | 0.13064 | 0.158923 | 0.175758 | 0.154882 | 0.219529 | 0.209428 | 0.162963 | 0.162963 | 0.200673 | 0.187205 | 0.139394 |
| 0.847352 | 0.150779 | 0.321495 | 0.713396 | 0.181931 | 0.290343 | 0.275389 | 0.166355 | 0.197508 | 0.36947 | 0.190031 | 0.168224 |
| 0.830808 | 0.181818 | 0.286616 | 0.668561 | 0.1875 | 0.314394 | 0.292929 | 0.202652 | 0.201389 | 0.362374 | 0.227273 | 0.206439 |
| 0.809855 | 0.19925 | 0.305303 | 0.684521 | 0.193894 | 0.305838 | 0.273166 | 0.196572 | 0.212641 | 0.371184 | 0.242635 | 0.208356 |
| 0.868962 | 0.365854 | 0.386418 | 0.726925 | 0.414634 | 0.321377 | 0.375418 | 0.338594 | 0.348159 | 0.445242 | 0.355811 | 0.351506 |
| 0.846813 | 0.142005 | 0.340291 | 0.693627 | 0.158032 | 0.273947 | 0.23593 | 0.162877 | 0.174804 | 0.355572 | 0.165486 | 0.159523 |

## Figure 7C – InDels (Insertions + Deletions)

| 1- p10/NT | | 2- p10/NT | | 1- p10/NT | | 2- p10/NT | | 1- p10/NT | | 2- p10/NT | |
|---|---|---|---|---|---|---|---|---|---|---|---|
| R1 | R2 | R1 | R2 | R1 | R2 | R1 | R2 | R1 | R2 | R1 | R2 |
| 0.111524 | 0.171004 | 0.092937 | 0.070632 | 0.189591 | 0.115242 | 0.137546 | 0.126394 | 0.156134 | 0.115242 | 0.122677 | 0.182156 |
| 0.067416 | 0.092697 | 0.050562 | 0.073034 | 0.117978 | 0.061798 | 0.087079 | 0.095506 | 0.073034 | 0.073034 | 0.106742 | 0.098315 |
| 0.064935 | 0.150649 | 0.077922 | 0.072727 | 0.148052 | 0.122078 | 0.124675 | 0.142857 | 0.148052 | 0.093506 | 0.109091 | 0.12987 |
| 0.084567 | 0.15222 | 0.103594 | 0.084567 | 0.160677 | 0.133192 | 0.116279 | 0.141649 | 0.12685 | 0.12685 | 0.128964 | 0.122622 |
| 0.072961 | 0.11588 | 0.072961 | 0.077253 | 0.118026 | 0.081545 | 0.072961 | 0.124464 | 0.094421 | 0.092275 | 0.100858 | 0.100858 |
| 0.067698 | 0.083172 | 0.040619 | 0.059961 | 0.104449 | 0.077369 | 0.077369 | 0.075435 | 0.088975 | 0.058027 | 0.077369 | 0.088975 |
| 0.087102 | 0.102178 | 0.077052 | 0.056951 | 0.102178 | 0.082077 | 0.072027 | 0.083752 | 0.095477 | 0.088777 | 0.088777 | 0.090452 |
| 0.074783 | 0.106087 | 0.066087 | 0.064348 | 0.097391 | 0.076522 | 0.078261 | 0.090435 | 0.09913 | 0.052174 | 0.073043 | 0.088696 |
| 0.059233 | 0.116725 | 0.08885 | 0.076655 | 0.11324 | 0.097561 | 0.094077 | 0.085366 | 0.094077 | 0.06446 | 0.118467 | 0.102787 |
| 0.09282 | 0.131349 | 0.085814 | 0.09282 | 0.112084 | 0.101576 | 0.117338 | 0.112084 | 0.115587 | 0.103327 | 0.10683 | 0.103327 |
| 0.070326 | 0.116638 | 0.084048 | 0.082333 | 0.137221 | 0.089194 | 0.099485 | 0.106346 | 0.085763 | 0.099485 | 0.128645 | 0.108062 |
| 0.093195 | 0.106509 | 0.071006 | 0.08432 | 0.090237 | 0.066568 | 0.079882 | 0.099112 | 0.069527 | 0.08432 | 0.079882 | 0.102071 |
| 0.074809 | 0.116031 | 0.079389 | 0.080916 | 0.109924 | 0.100763 | 0.10687 | 0.116031 | 0.10229 | 0.083969 | 0.087023 | 0.109924 |
| 0.091493 | 0.105939 | 0.081862 | 0.083467 | 0.123596 | 0.093098 | 0.099518 | 0.123596 | 0.109149 | 0.078652 | 0.104334 | 0.101124 |
| 0.069841 | 0.107937 | 0.096825 | 0.073016 | 0.095238 | 0.098413 | 0.088889 | 0.115873 | 0.090476 | 0.061905 | 0.077778 | 0.101587 |
| 0.06993 | 0.096503 | 0.044755 | 0.053147 | 0.100699 | 0.06993 | 0.083916 | 0.103497 | 0.097902 | 0.062937 | 0.067133 | 0.099301 |
| 0.065693 | 0.091971 | 0.059854 | 0.064234 | 0.078832 | 0.059854 | 0.075912 | 0.074453 | 0.067153 | 0.068613 | 0.070073 | 0.067153 |
| 0.098649 | 0.140541 | 0.086486 | 0.094595 | 0.144595 | 0.114865 | 0.116216 | 0.131081 | 0.136486 | 0.104054 | 0.148649 | 0.135135 |
| 0.088193 | 0.13229 | 0.113798 | 0.099573 | 0.16074 | 0.112376 | 0.129445 | 0.145092 | 0.129445 | 0.113798 | 0.130868 | 0.157895 |
| 0.069987 | 0.110363 | 0.091521 | 0.064603 | 0.117093 | 0.090175 | 0.094213 | 0.110363 | 0.100942 | 0.096904 | 0.130552 | 0.115747 |
| 0.069858 | 0.09185 | 0.071151 | 0.050453 | 0.100906 | 0.073739 | 0.080207 | 0.09185 | 0.086675 | 0.084088 | 0.07762 | 0.087969 |
| 0.09484 | 0.112971 | 0.096234 | 0.090656 | 0.114365 | 0.108787 | 0.107392 | 0.126918 | 0.09205 | 0.110181 | 0.119944 | 0.11855 |
| 0.086675 | 0.102199 | 0.076326 | 0.081501 | 0.104787 | 0.089263 | 0.082794 | 0.104787 | 0.108668 | 0.073739 | 0.094437 | 0.108668 |
| 0.072533 | 0.109394 | 0.071344 | 0.057075 | 0.102259 | 0.0761 | 0.080856 | 0.109394 | 0.102259 | 0.06302 | 0.08918 | 0.108205 |
| 0.073384 | 0.081051 | 0.077766 | 0.052574 | 0.087623 | 0.075575 | 0.078861 | 0.09529 | 0.083242 | 0.069003 | 0.083242 | 0.094195 |
| 0.058608 | 0.075092 | 0.052198 | 0.059524 | 0.081502 | 0.076923 | 0.071429 | 0.075092 | 0.076007 | 0.062271 | 0.077839 | 0.090659 |
| 0.086649 | 0.115827 | 0.082228 | 0.068966 | 0.108753 | 0.08046 | 0.098143 | 0.106101 | 0.114058 | 0.079576 | 0.108753 | 0.129973 |
| 0.087855 | 0.120586 | 0.093885 | 0.080965 | 0.136951 | 0.096469 | 0.101637 | 0.124031 | 0.108527 | 0.086133 | 0.106804 | 0.118002 |
| 0.074196 | 0.114592 | 0.077494 | 0.088211 | 0.109646 | 0.083265 | 0.093982 | 0.116241 | 0.093157 | 0.075021 | 0.121187 | 0.112119 |
| 0.077635 | 0.103989 | 0.081197 | 0.088319 | 0.100427 | 0.075499 | 0.096866 | 0.099003 | 0.081909 | 0.070513 | 0.104701 | 0.079772 |
| 0.057239 | 0.101684 | 0.065993 | 0.080135 | 0.082155 | 0.086195 | 0.086195 | 0.088215 | 0.078788 | 0.06532 | 0.080808 | 0.093603 |
| 0.075389 | 0.113396 | 0.094704 | 0.082866 | 0.102804 | 0.089097 | 0.090966 | 0.11215 | 0.103427 | 0.086604 | 0.102181 | 0.107165 |
| 0.081439 | 0.111111 | 0.083965 | 0.068813 | 0.114899 | 0.10101 | 0.099116 | 0.111111 | 0.097854 | 0.073864 | 0.094697 | 0.117424 |
| 0.08677 | 0.091055 | 0.087841 | 0.077129 | 0.094804 | 0.087841 | 0.081414 | 0.087306 | 0.098018 | 0.080878 | 0.083021 | 0.105517 |
| 0.101865 | 0.131516 | 0.107604 | 0.108082 | 0.132473 | 0.111908 | 0.111908 | 0.126734 | 0.120038 | 0.098039 | 0.116691 | 0.13869 |
| 0.097279 | 0.130078 | 0.101006 | 0.08498 | 0.120388 | 0.100261 | 0.111815 | 0.118897 | 0.120015 | 0.09877 | 0.118151 | 0.131569 |
| 0.144981 | 0.182156 | 0.152416 | 0.137546 | 0.137546 | 0.185874 | 0.189591 | 0.167286 | 0.178439 | 0.159851 | 0.156134 | 0.200743 |
| 0.044944 | 0.115169 | 0.067416 | 0.123596 | 0.087079 | 0.08427 | 0.123596 | 0.095506 | 0.11236 | 0.050562 | 0.075843 | 0.101124 |
| 0.098701 | 0.148052 | 0.077922 | 0.106494 | 0.109091 | 0.096104 | 0.127273 | 0.158442 | 0.14026 | 0.096104 | 0.119481 | 0.14026 |
| 0.175476 | 0.169133 | 0.120507 | 0.128964 | 0.109937 | 0.097252 | 0.158562 | 0.169133 | 0.158562 | 0.095137 | 0.122622 | 0.164905 |
| 0.103004 | 0.124464 | 0.107296 | 0.126609 | 0.085837 | 0.107296 | 0.124464 | 0.120172 | 0.137339 | 0.096567 | 0.111588 | 0.135193 |
| 0.069632 | 0.10058 | 0.071567 | 0.104449 | 0.071567 | 0.088975 | 0.106383 | 0.106383 | 0.104449 | 0.081238 | 0.088975 | 0.123791 |
| 0.075377 | 0.122278 | 0.090452 | 0.108878 | 0.100503 | 0.088777 | 0.108878 | 0.102178 | 0.135678 | 0.092127 | 0.093802 | 0.130653 |
| 0.085217 | 0.130435 | 0.081739 | 0.118261 | 0.083478 | 0.106087 | 0.137391 | 0.106087 | 0.09913 | 0.076522 | 0.052174 | 0.121739 |
| 0.111498 | 0.134146 | 0.087108 | 0.11324 | 0.081882 | 0.097561 | 0.12892 | 0.109756 | 0.114983 | 0.116725 | 0.097561 | 0.146341 |
| 0.105079 | 0.178634 | 0.124343 | 0.161121 | 0.105079 | 0.119089 | 0.175131 | 0.161121 | 0.161121 | 0.134851 | 0.134851 | 0.203152 |
| 0.118353 | 0.123499 | 0.099485 | 0.133791 | 0.089194 | 0.101201 | 0.102916 | 0.123499 | 0.121784 | 0.096055 | 0.096055 | 0.121784 |
| 0.079882 | 0.121302 | 0.078402 | 0.107988 | 0.090237 | 0.094675 | 0.113905 | 0.099112 | 0.102071 | 0.087278 | 0.081361 | 0.106509 |
| 0.090076 | 0.140458 | 0.105344 | 0.129771 | 0.074809 | 0.108397 | 0.140458 | 0.109924 | 0.117557 | 0.10687 | 0.100763 | 0.125191 |
| 0.102729 | 0.11878 | 0.097913 | 0.12199 | 0.104334 | 0.094703 | 0.11557 | 0.11557 | 0.139647 | 0.085072 | 0.088283 | 0.125201 |
| 0.112698 | 0.134921 | 0.101587 | 0.133333 | 0.085714 | 0.104762 | 0.133333 | 0.131746 | 0.149206 | 0.092063 | 0.104762 | 0.142857 |
| 0.083916 | 0.093706 | 0.064336 | 0.096503 | 0.054545 | 0.076923 | 0.103497 | 0.102098 | 0.08951 | 0.082517 | 0.096503 | 0.11049 |
| 0.083212 | 0.105109 | 0.091971 | 0.108029 | 0.062774 | 0.075912 | 0.113869 | 0.10365 | 0.118248 | 0.086131 | 0.087591 | 0.122628 |
| 0.095946 | 0.087838 | 0.078378 | 0.094595 | 0.124324 | 0.078378 | 0.128378 | 0.12027 | 0.121622 | 0.093243 | 0.113514 | 0.139189 |
| 0.083926 | 0.118065 | 0.075391 | 0.100996 | 0.108108 | 0.096728 | 0.106686 | 0.106686 | 0.122333 | 0.083926 | 0.102418 | 0.135135 |
| 0.090175 | 0.118439 | 0.091521 | 0.123822 | 0.091521 | 0.086137 | 0.117093 | 0.10498 | 0.115747 | 0.083445 | 0.078062 | 0.117093 |
| 0.111255 | 0.126779 | 0.094437 | 0.119017 | 0.09185 | 0.100906 | 0.13066 | 0.107374 | 0.111255 | 0.094437 | 0.102199 | 0.121604 |
| 0.100418 | 0.114365 | 0.100418 | 0.129707 | 0.097629 | 0.09484 | 0.114365 | 0.126918 | 0.119944 | 0.080893 | 0.104603 | 0.11576 |
| 0.046572 | 0.089263 | 0.058215 | 0.084088 | 0.094437 | 0.056921 | 0.084088 | 0.087969 | 0.080207 | 0.058215 | 0.069858 | 0.081501 |
| 0.08918 | 0.130797 | 0.079667 | 0.11415 | 0.096314 | 0.080856 | 0.123662 | 0.099881 | 0.116528 | 0.078478 | 0.097503 | 0.121284 |
| 0.107338 | 0.134721 | 0.09529 | 0.124863 | 0.078861 | 0.104053 | 0.120482 | 0.131435 | 0.124863 | 0.077766 | 0.099671 | 0.136911 |
| 0.087912 | 0.10348 | 0.084249 | 0.094322 | 0.067766 | 0.076923 | 0.107143 | 0.111722 | 0.101648 | 0.07326 | 0.081502 | 0.122711 |
| 0.089302 | 0.095491 | 0.070734 | 0.099027 | 0.10168 | 0.078691 | 0.104332 | 0.087533 | 0.10168 | 0.083112 | 0.08046 | 0.105217 |
| 0.08441 | 0.106804 | 0.082687 | 0.109388 | 0.102498 | 0.080965 | 0.103359 | 0.095607 | 0.103359 | 0.080965 | 0.089578 | 0.105943 |
| 0.114592 | 0.134378 | 0.119538 | 0.136851 | 0.083265 | 0.095631 | 0.126958 | 0.128607 | 0.1385 | 0.099753 | 0.091509 | 0.148392 |
| 0.088319 | 0.10114 | 0.084046 | 0.089031 | 0.097578 | 0.067664 | 0.09188 | 0.080484 | 0.089031 | 0.068376 | 0.064815 | 0.098291 |
| 0.072727 | 0.084848 | 0.063973 | 0.070707 | 0.074074 | 0.066667 | 0.086869 | 0.091582 | 0.088889 | 0.066667 | 0.056566 | 0.092256 |
| 0.082866 | 0.115888 | 0.080997 | 0.11838 | 0.069159 | 0.094704 | 0.107165 | 0.105919 | 0.107788 | 0.088474 | 0.086604 | 0.137072 |
| 0.085859 | 0.098485 | 0.099747 | 0.09154 | 0.084596 | 0.082071 | 0.104167 | 0.094697 | 0.108586 | 0.087753 | 0.080177 | 0.109848 |
| 0.086235 | 0.100161 | 0.075522 | 0.08677 | 0.086235 | 0.081414 | 0.113016 | 0.093198 | 0.104446 | 0.09052 | 0.087841 | 0.099089 |
| 0.106648 | 0.13056 | 0.098996 | 0.129603 | 0.115734 | 0.099952 | 0.131994 | 0.116212 | 0.13056 | 0.08704 | 0.094213 | 0.129603 |
| 0.101379 | 0.108833 | 0.085725 | 0.101379 | 0.112561 | 0.082743 | 0.106597 | 0.104734 | 0.107343 | 0.08498 | 0.080134 | 0.105479 |

# Figure 7D - CNV

| Chrom. | 1 | | 2 | | 1 | | 2 | | 1 | | 2 | |
|---|---|---|---|---|---|---|---|---|---|---|---|---|
| | R1 | R2 | R1 | R2 | R1 | R2 | R1 | R2 | R1 | R2 | R1 | R2 |
| LmjF.01 | 0.173353 | 0.082362 | 0.06223 | 0.091335 | 0.061864 | 0.092604 | 0.10192 | 0.03498 | 0.24458 | 0.16291 | 0.42114 | 0.021943 |
| LmjF.02 | 0.067578 | 0.014881 | 0.017147 | 0.004527 | 0.060428 | 0.019136 | 0.03175 | -0.0198 | 0.05628 | 0.00538 | 0.04037 | 0.008755 |
| LmjF.03 | 0.033437 | 0.021158 | 0.019975 | 0.002901 | 0.04333 | -0.01601 | 0.01457 | -0.0025 | 0.03291 | 0.01071 | 0.03347 | 0.046194 |
| LmjF.04 | -0.02889 | 0.052991 | 0.018237 | 0.05562 | 0.053034 | 0.063366 | 0.07185 | 0.04273 | 0.09408 | 0.02588 | 0.07592 | 0.034929 |
| LmjF.05 | 0.011202 | 0.043246 | 0.023614 | 0.048378 | 0.03964 | 0.064471 | 0.05275 | 0.02889 | 0.0729 | 0.01446 | 0.09016 | 0.023509 |
| LmjF.06 | 0.07479 | -0.01408 | 0.017147 | -0.02176 | 0.037133 | -0.03547 | -0.00815 | -0.0416 | 0.01144 | 0.00649 | 0.00173 | 0.029084 |
| LmjF.07 | 0.02536 | -0.00053 | 0.016858 | 0.012925 | 0.040325 | -0.00107 | 0.02005 | -0.0134 | 0.04157 | 0.01402 | 0.02758 | 0.022883 |
| LmjF.08 | -0.00871 | 0.059289 | 0.030363 | 0.068184 | -0.42076 | -0.15207 | -0.41639 | -0.427 | -0.2098 | -0.18406 | -0.23017 | -0.02602 |
| LmjF.09 | -0.01052 | 0.033882 | 0.021056 | 0.041059 | 0.039567 | 0.015852 | 0.0409 | 0.0212 | 0.06188 | 0.01945 | 0.05096 | 0.030343 |
| LmjF.10 | -0.03251 | 0.016091 | 0.006164 | 0.031552 | 0.02873 | -0.00192 | 0.03452 | 0.04073 | 0.05116 | 0.03852 | 0.04788 | 0.034878 |
| LmjF.11 | 0.010567 | -0.01876 | 0.012914 | -0.00607 | 0.043079 | -0.04064 | 0.00796 | 0.00122 | 0.02594 | 0.03192 | 0.02041 | 0.028754 |
| LmjF.12 | -0.04553 | 0.022185 | 0.017323 | 0.022146 | -0.18253 | -0.10505 | -0.17162 | -0.1486 | -0.2962 | -0.24747 | -0.16261 | -0.4875 |
| LmjF.13 | 0.030227 | -0.01316 | 0.020255 | -0.00195 | 0.029416 | -0.02659 | 0.00393 | -0.0173 | 0.02883 | 0.016 | 0.01529 | 0.027916 |
| LmjF.14 | -0.06731 | 0.029252 | 0.017971 | 0.037567 | -0.05815 | -0.02015 | -0.01117 | 0.00509 | 0.04246 | -0.02646 | 0.01976 | -0.01776 |
| LmjF.15 | 0.015101 | 0.021675 | 0.025104 | 0.022292 | 0.02764 | -0.01105 | 0.01575 | 0.00842 | 0.03612 | 0.01679 | 0.03134 | 0.028292 |
| LmjF.16 | 0.030207 | -0.01311 | 0.013792 | -0.00795 | 0.041973 | -0.04169 | 0.00636 | -0.0207 | 0.00949 | 0.0137 | 0.01071 | 0.02115 |
| LmjF.17 | -0.00867 | 0.008356 | 0.019425 | 0.015243 | 0.030221 | 9.75E-06 | 0.01456 | 0.01116 | 0.02002 | 0.01957 | 0.02552 | 0.028095 |
| LmjF.18 | -0.04196 | -0.00553 | 0.011961 | 0.015332 | 0.021723 | -0.05893 | 0.00563 | -0.0025 | 0.02744 | 0.02811 | 0.02012 | 0.016644 |
| LmjF.19 | -0.02301 | -0.00148 | 0.023029 | 0.006824 | 0.01778 | -0.01384 | 0.00495 | 0.0028 | 0.02223 | 0.02156 | 0.01089 | 0.012197 |
| LmjF.20 | 0.004316 | -0.01389 | 0.012402 | 0.003231 | 0.024998 | -0.0283 | 0.05285 | -0.0043 | 0.01351 | 0.02378 | 0.00657 | 0.027936 |
| LmjF.21 | 0.0033 | 0.012238 | 0.018426 | 0.022388 | 0.030161 | -0.0199 | 0.01572 | -0.0031 | 0.04425 | 0.01345 | 0.0298 | 0.019228 |
| LmjF.22 | -0.00215 | -0.00914 | 0.015615 | 0.011985 | 0.020372 | -0.04671 | 0.00923 | -0.0001 | 0.02771 | 0.02587 | 0.01677 | 0.014798 |
| LmjF.23 | -0.00719 | -0.01595 | 0.018637 | 0.006329 | -0.01688 | -0.06796 | -0.01746 | -0.0128 | -0.059 | -0.04513 | -0.02191 | -0.07464 |
| LmjF.24 | 0.0255 | -0.00764 | 0.018284 | 0.001715 | 0.028535 | -0.00913 | -0.00104 | -0.0148 | 0.01696 | 0.01288 | 0.01423 | 0.018091 |
| LmjF.25 | -0.00865 | 0.001765 | 0.021004 | 0.014495 | 0.03468 | -0.00761 | -0.00496 | 0.01236 | 0.02272 | 0.01541 | 0.0208 | 0.009898 |
| LmjF.26 | 0.017061 | -0.0124 | 0.026255 | -0.00381 | 0.027672 | -0.01852 | -0.01363 | -0.0233 | 0.00884 | 0.00663 | -0.00256 | 0.010106 |
| LmjF.27 | 0.034394 | -0.03456 | 0.012076 | -0.02153 | 0.024148 | -0.06048 | -0.01992 | -0.0615 | -0.0045 | -0.03375 | -0.01864 | -0.03687 |
| LmjF.28 | -0.00923 | -0.02295 | 0.020359 | -0.00552 | 0.020727 | -0.06006 | -0.01519 | -0.0222 | 0.00736 | 0.01795 | -0.00211 | 0.007323 |
| LmjF.29 | -0.03307 | -0.01984 | 0.019069 | -0.0048 | 0.025377 | -0.05111 | -0.00699 | -0.0161 | 0.01483 | 0.00921 | 0.00068 | 0.005874 |
| LmjF.30 | 0.010246 | -0.03611 | 0.021383 | -0.01809 | 0.017803 | -0.06502 | -0.03136 | -0.0188 | -0.0025 | 0.01427 | 0.00501 | 0.004747 |
| LmjF.31 | 0.040252 | -0.03165 | 0.023807 | -0.00662 | -0.05586 | -0.05962 | -0.0592 | -0.0916 | -0.1253 | -0.09284 | -0.12773 | -0.04836 |
| LmjF.32 | -0.01621 | -0.03943 | 0.013023 | -0.02628 | 0.019855 | -0.06499 | -0.03263 | -0.04 | -0.0068 | 0.01787 | -0.02486 | 0.010499 |
| LmjF.33 | -0.01376 | -0.01365 | 0.030568 | -0.00449 | 0.010796 | -0.03067 | -0.02702 | -0.0306 | -0.0051 | 0.01364 | -0.01632 | 0.007194 |
| LmjF.34 | -0.00907 | -0.03397 | 0.021552 | -0.02649 | 0.016536 | -0.04401 | -0.03014 | -0.0378 | -0.0097 | 0.00636 | -0.01913 | -0.00582 |
| LmjF.35 | -0.01733 | -0.03942 | 0.006012 | -0.03008 | 0.018033 | -0.03219 | -0.03084 | -0.0334 | -0.0026 | 0.02162 | -0.02962 | 0.00815 |
| LmjF.36 | 0.007569 | -0.04558 | 0.020414 | -0.02773 | 0.014299 | -0.06217 | -0.04719 | -0.0511 | -0.017 | 0.00091 | -0.03286 | -0.00562 |

# Supplementary Figure 1 – Growth Curve

| Days | CC1 | | Cas9T7 | | $^{myc}$ATR | | $^{myc}$ATR$^{\Delta C\ +/-}$ | | $^{myc}$ATR$^{\Delta C\ -/-}$ (Cl1) | | $^{myc}$ATR$^{\Delta C\ -/-}$ (Cl8) | |
|---|---|---|---|---|---|---|---|---|---|---|---|---|
| 0 | 200000 | 200000 | 200000 | 200000 | 200000 | 200000 | 200000 | 200000 | 200000 | 200000 | 200000 | 200000 |
| 1 | 1100000 | 1100000 | 1400000 | 1500000 | 1200000 | 1100000 | 1300000 | 1500000 | 1000000 | 500000 | 790000 | 760000 |
| 2 | 13000000 | 15000000 | 16000000 | 9300000 | 15000000 | 13000000 | 19000000 | 18000000 | 5000000 | 7300000 | 5800000 | 6000000 |
| 3 | 25000000 | 23000000 | 9300000 | 12000000 | 21000000 | 20000000 | 27000000 | 26000000 | 22000000 | 21000000 | 21000000 | 21000000 |
| 4 | 18000000 | 17000000 | 8000000 | 17000000 | 24000000 | 18000000 | 30000000 | 31000000 | 16000000 | 18000000 | 17000000 | 19000000 |
| 5 | 22000000 | 15000000 | 8600000 | 9300000 | 23000000 | 21000000 | 29000000 | 35000000 | 27000000 | 31000000 | 22000000 | 26000000 |
| 6 | 19000000 | 14000000 | 7800000 | 11000000 | 23000000 | 22000000 | 34000000 | 38000000 | 20000000 | 28000000 | 17000000 | 17000000 |

# Supplementary Figure 3 – EdU intensity of positive cells

| NT | | | | Chronic | | | | | | Acute | | | | | | | |
|---|---|---|---|---|---|---|---|---|---|---|---|---|---|---|---|---|---|
| $^{myc}ATR^{\Delta C -/-}$ (Cl1) | | $^{myc}ATR^{\Delta C -/-}$ (Cl8) | | $^{myc}ATR$ | | $^{myc}ATR^{\Delta C -/-}$ (Cl1) | | $^{myc}ATR^{\Delta C -/-}$ (Cl8) | | $^{myc}ATR$ | | $^{myc}ATR^{\Delta C -/-}$ (Cl1) | | $^{myc}ATR^{\Delta C -/-}$ (Cl8) | | CN | |
| R1 | R2 | R1 | R2 | R1 | R2 | R1 | R2 | R1 | R2 | R1 | R2 | R1 | R2 | R1 | R2 | R1 | R2 |
| 8446.143 | 5399.75 | 5659.125 | 24802.31 | 11794.5 | 11948 | 8358.571 | 13717.84 | 4933 | 9481.125 | 15465.15 | 18543.58 | 5434.333 | 11483.67 | 4933 | 18387.79 | 1421.188 | 1203.542 |
| 6608.75 | 6783.833 | 12868.9 | 11835.91 | 17498.69 | 6113.882 | 5905.147 | 9075.706 | 8782.5 | 19141.17 | 7457.885 | 8349.156 | 5025.167 | 7162.917 | 8782.5 | 19041.86 | 1234.524 | 1209.125 |
| 4471.143 | 9587.905 | 6397.25 | 14315.15 | 12887.42 | 11037.65 | 2261.115 | 17041.72 | 7617.25 | 6377.833 | 10941.1 | 9623.941 | 8490.375 | 14014.75 | 7617.25 | 7475.235 | 1216.192 | 1202 |
| 10980.91 | 5866.938 | 13535.06 | 16233.75 | 13117.9 | 11767.81 | 4692.524 | 4715.029 | 3061.769 | 8180.577 | 7520.4 | 16101.39 | 2297.938 | 5239.045 | 3061.769 | 6470.967 | 1171.406 | 1091.619 |
| 3358.476 | 8308.192 | 9412.812 | 30826.35 | 8301.938 | 17884.69 | 4980.231 | | 6044.459 | 17209.62 | 10428.38 | 31069 | 2759.188 | 13136.5 | 6044.459 | 5702.882 | 1422.714 | 1379.419 |
| 3978 | 11436.71 | 10231.44 | 44681.79 | 7115.4 | 8777.219 | 1972.417 | | 4747.647 | 7663.853 | 11067.35 | 10841.77 | 3388.722 | 10703.13 | 4747.647 | 8561.871 | 1160.231 | 1689.083 |
| 4699.25 | 8792.905 | 11725.73 | 20348.67 | 15740.06 | 14205.36 | 2111.55 | | 4934.231 | 10588.49 | 7056.3 | 6627.147 | 2361.269 | 7222.583 | 4934.231 | 9895.278 | 1208.25 | 1615.417 |
| 3349.312 | 6714.562 | 6675.562 | 13793.48 | 16395.43 | 14213.07 | 6050.167 | | 5900.538 | 7820.095 | 3507.808 | 14902.14 | 7997.81 | 5396.25 | 5900.538 | 5833.933 | 1415 | 1546.812 |
| 5736.125 | 4379.375 | 11243.94 | 16594.8 | 15672.25 | 13762.92 | | | 4341.125 | 22722.63 | 5612.355 | 8705.838 | 2093.387 | 5156.583 | 4341.125 | 15054.06 | 1722.952 | 1097.188 |
| 5188.2 | 7977 | 9461 | 26927.81 | 11653.95 | 5333.176 | | | 3195.028 | | 5115.115 | 24937.39 | 8781.562 | 7884.35 | 3195.028 | 3845.029 | 1314.476 | 1396.25 |
| 10303.38 | 19715.24 | 7790.083 | 50226.95 | 8893.077 | 8315.781 | | | 3963.846 | | 6838.423 | 12794.17 | 17172.71 | 7120.538 | 3963.846 | 2658.889 | 1204.095 | 1354.182 |
| 4600.55 | 11726.31 | 5312.125 | 41191.75 | 12945.27 | 15785.15 | | | 4314.952 | | 7461.5 | 16387.19 | 8409 | 4650.476 | 4314.952 | 14514.35 | 1433.688 | 1497.167 |
| 5134.917 | 13526.94 | 4453.188 | 31241.94 | 18493.42 | 9599.381 | | | 9535.25 | | 5377.857 | 17577.53 | 5429.613 | 6224.346 | 9535.25 | 17982.73 | 1210.154 | 1505.857 |
| 6503.333 | 17572.55 | 15050.67 | 25321.31 | 13232.3 | 5318.192 | | | 7810.769 | | 24384.9 | 22321.64 | 13245.2 | 18326.27 | 7810.769 | 12543.85 | 1378.182 | 1463.692 |
| 6185.438 | 10221.38 | 10830 | 23961.43 | 10237.7 | 5130.719 | | | 11104.38 | | 6646.846 | 10762.54 | 9539.238 | 16929.85 | 11104.38 | 7530.909 | 1331.083 | 1208.846 |
| 8673.75 | 7456.452 | 29148.44 | 49842.58 | 9744.438 | 8206.871 | | | 3671.625 | | 8280.5 | 10766.35 | | 9868.688 | 3671.625 | 8893 | 1745.45 | 1208.846 |
| 6971.125 | 12530.05 | 30524.46 | 35012.29 | 12786.88 | 10006.43 | | | 9109.95 | | 9347.85 | 14769.74 | | 13007 | 9109.95 | 8462.167 | 1395.333 | 1198.538 |
| 4012.923 | 3980.808 | 6491.688 | 7836.81 | 19695.19 | 17399 | | | 4375.75 | | 6681.5 | 16013.03 | | 8750.05 | 4375.75 | 5839.7 | 1398.346 | 963.406 |
| 3684.231 | 10227.94 | 18292.24 | 11215.55 | 8712.308 | 10988.68 | | | 3217.857 | | 6266.419 | 7026.306 | | 5161.871 | 3217.857 | | 1342.1 | 1455.238 |
| 3704.6 | 12741.75 | 19451.23 | 37310.35 | 13996 | 7036.811 | | | | | 19076.75 | 9275.097 | | 5443.971 | | | 1401.5 | 1327.231 |
| 19078.67 | 5973 | 28440.83 | 15226.57 | 8128.524 | 4220.386 | | | | | 13596.4 | 13234.89 | | 4486.308 | | | 1294.952 | 1257.545 |
| 39914.17 | 8995.364 | 25215.33 | 21422.46 | 8590.562 | 6163.235 | | | | | 12221.44 | 13913.94 | | 14924.6 | | | 1510.35 | 1545.375 |
| 6296.312 | 10333.64 | 13795.63 | 9435.731 | 18978.24 | 17524.35 | | | | | 6054.273 | 7339.094 | | 13088.32 | | | 1374.667 | 1283.095 |
| 7977.333 | 18888 | 7898.1 | 21994.34 | 20054.65 | 16530.88 | | | | | 5510.812 | 9197.423 | | 13663.55 | | | 1227.167 | 1273.441 |
| 15187.29 | 5816 | 13587 | 7269.857 | 14822.19 | 15586.84 | | | | | 5807.65 | 4998.065 | | 16900.04 | | | 1303.692 | 1161 |
| 13808.52 | 6353.625 | 8766.062 | 25315.2 | 9002.308 | 11875 | | | | | 12330.19 | 24080.47 | | 5031.462 | | | 1520.062 | 1649.714 |
| 4078.125 | 7440.571 | 6158.938 | 13775.19 | 8077.583 | 3767.714 | | | | | 10986.55 | 9500.059 | | 5037.031 | | | 1575.077 | 1578.083 |
| 7625.115 | 11079.88 | 15259.05 | 27763.56 | 8370.912 | 4674.188 | | | | | 6780.381 | 13287.84 | | 17064.46 | | | 1390.5 | 1779.381 |
| 19852.57 | 19108.25 | 12926.83 | 20261.44 | 24859.06 | 4144.048 | | | | | 10341 | 9252.312 | | 4742.611 | | | 1319.312 | 1008.25 |
| 13313.81 | 11998.62 | 32553.94 | 43060.76 | 11234.56 | 4051.581 | | | | | 6628.375 | 9210.412 | | 5631.269 | | | 1265.125 | 1239.714 |
| 7104.917 | 12126.83 | 9944.5 | 39170.83 | 7034.154 | 3771.423 | | | | | 10631.25 | 9471.5 | | 5011.071 | | | 1291.385 | 1138.286 |
| 9746.5 | 13832.19 | 17251 | 37950.38 | 15502.67 | 10181.08 | | | | | 7454.438 | 5248.265 | | 4621.077 | | | 1492.438 | 1329.188 |
| 20374.19 | 10514 | 12064.25 | 23697.62 | 29922.52 | 11933.92 | | | | | 7468.3 | 4791.615 | | 5266.125 | | | 1506.688 | 1291.346 |
| 8873.6 | 8203.81 | 24239.44 | 37171.15 | 7757.333 | 22844.88 | | | | | 7014.2 | 8061.9 | | 5224.5 | | | 1433.55 | 1184.625 |
| 12203.13 | 7658.417 | 13241.95 | 27115.04 | 11928.81 | 10796.7 | | | | | 23667.58 | 20047.81 | | 5039.5 | | | 1427.731 | 1538.812 |
| 12101.31 | 10104.48 | 30748.88 | 16879.52 | 28684.48 | 19503.95 | | | | | 11635.15 | 8593.577 | | 12130.39 | | | 1255.062 | 1262.077 |
| 11121.76 | 15224.38 | 10360.31 | 15889.05 | 9649.45 | 22517.19 | | | | | 27275.75 | 7103.6 | | 14220.78 | | | 1370.35 | 1142.038 |
| 9488.85 | 9945.7 | 11907.25 | 32875.55 | 19827.73 | 6058.871 | | | | | 12910.14 | 16057.9 | | 9589.077 | | | 1371.714 | 1313.538 |
| 11386.67 | 11405.83 | 15122 | 24234.05 | 16452.69 | 7720.281 | | | | | 12273.61 | 11214.43 | | 11638.16 | | | 1294.762 | 1456.538 |
| 4911 | 13084 | 24528.86 | 34262.24 | 12894.77 | 10433.64 | | | | | 14294.2 | 34740 | | 10593.23 | | | 1407.619 | 1556.429 |
| 27122.81 | 14065.42 | 8423 | 14653.06 | 11902.19 | 8484.286 | | | | | 10247.23 | 23130.54 | | 6089.162 | | | 1206.538 | 898.095 |
| 8235.167 | 12041.5 | 7895.688 | 24342.53 | 8292.308 | 7097.688 | | | | | 5284.571 | 9887.375 | | 4174.614 | | | 1337.429 | 1282.476 |
| 12586.85 | 6901.5 | 8390.938 | 30116.35 | 10656.24 | 7675.833 | | | | | 8673 | 14301.17 | | 7176.5 | | | 1463.25 | 1373.429 |
| 18340.54 | 19740.43 | 6620.188 | 32483.66 | 18453.25 | 6758.062 | | | | | 5419.583 | 7635.333 | | 4163.269 | | | 1200.438 | 1654 |
| 18306.19 | 8436.125 | 6071.438 | 19523.78 | 14779.22 | | | | | | 11011.96 | 31171.35 | | 7377.417 | | | 1480.333 | 1637.857 |
| 4017.654 | 7341.357 | 15703.05 | 34985.77 | 6750.25 | | | | | | 8438.5 | 7198.654 | | 6743.438 | | | 1377.654 | 1351.938 |
| 18065.91 | 7100.273 | 12550.19 | 34451.95 | 11620.69 | | | | | | 19447.8 | 11133 | | 5354.8 | | | 1322.423 | 1392.4 |
| 10583.39 | 10911.25 | 13815.05 | 25218.77 | 11659 | | | | | | 7918.619 | 12442.13 | | 5666.25 | | | 1387.269 | 1770.875 |
| 16671.88 | 15630.88 | 8874.125 | 19063 | | | | | | | 10323.2 | 7164.7 | | 5624.639 | | | 1328.562 | 1075.714 |
| 14451.13 | 9621.154 | 27697.56 | 34427.6 | | | | | | | 5060.432 | 5715.577 | | 7116.417 | | | 1217.577 | 1320.812 |
| 6436.455 | 10936.19 | 10894.58 | 19853.19 | | | | | | | 15354.1 | 6872.231 | | 7113.273 | | | 1213 | 1602 |
| 14303.43 | 4691 | 14488.31 | 21947.3 | | | | | | | 9520.423 | 31907.33 | | 7169 | | | 1356.094 | 1197.5 |
| 6936.909 | 9143.25 | 9613.75 | 36389.44 | | | | | | | 11051.31 | 20409.81 | | 5825 | | | 1563.75 | 1090.062 |
| 6577 | 5971.19 | 7262.857 | 27750.47 | | | | | | | 32369.33 | 8738.2 | | | | | 1397.354 | 1188.25 |
| 9854.55 | 9982.833 | | 16530.24 | | | | | | | 6482.194 | 9677.75 | | | | | 1334.25 | 1438.857 |
| 20854.81 | 6037.833 | | 15764.04 | | | | | | | 10731.8 | 9598.125 | | | | | 1406.125 | 1230.6 |
| 8993 | | | 22033.27 | | | | | | | 18243.25 | 25935.86 | | | | | 1351.438 | 983.818 |
| 13617.85 | | | 20503.65 | | | | | | | 12082.46 | 14940.5 | | | | | 1337.323 | 1084.81 |
| 19621.75 | | | 43096.35 | | | | | | | 10918.94 | 5781 | | | | | 1218.01 | 1509.742 |
| 18464.38 | | | 31494.16 | | | | | | | 11034.88 | 5969.231 | | | | | 1307.143 | 1558.615 |
| 7517.6 | | | 42608.97 | | | | | | | 7269.8 | 8716.375 | | | | | 1269.962 | 1549.125 |
| 6593.286 | | | 35707.32 | | | | | | | 14385.63 | 4918.708 | | | | | 1365.118 | 1119.667 |
| 8040.095 | | | 28052.36 | | | | | | | 30574.05 | 9973.438 | | | | | 1225.385 | 1305.25 |
| 11933.25 | | | 16755.46 | | | | | | | 10495.46 | 14097.5 | | | | | 1343.4 | 1226.75 |
| 15846.88 | | | 18146 | | | | | | | 5245.5 | 15605.08 | | | | | 1441.688 | 1181.654 |
| 14625.56 | | | 26109.87 | | | | | | | | 11036.9 | | | | | 1151.423 | 1320.429 |
| | | | 27016.56 | | | | | | | | 12038.13 | | | | | 1254.143 | 1331.905 |
| | | | 29807.88 | | | | | | | | 7204.05 | | | | | 1313.161 | 1243.688 |
| | | | 37529.19 | | | | | | | | 4852.562 | | | | | 1268.484 | 1495.8 |
| | | | 38972.81 | | | | | | | | 17536.85 | | | | | 1336.9 | 1155.077 |
| | | | 33144.83 | | | | | | | | 10511.65 | | | | | 1278.231 | 1165.667 |
| | | | 25139.73 | | | | | | | | 4142.048 | | | | | 1343.846 | 1024 |
| | | | 9283.031 | | | | | | | | 6967.81 | | | | | 1272.95 | 1049.571 |
| | | | | | | | | | | | 30713.5 | | | | | 1322.962 | 994.25 |
| | | | | | | | | | | | 17009.95 | | | | | 1241.692 | 1133.095 |
| | | | | | | | | | | | 45990.31 | | | | | 1214.95 | 987.5 |
| | | | | | | | | | | | 3788.85 | | | | | 1203.25 | 1209.375 |
| | | | | | | | | | | | 9941.095 | | | | | 1339.562 | 1039.615 |
| | | | | | | | | | | | 14284.71 | | | | | 1194.824 | 1210.75 |
| | | | | | | | | | | | 7049.917 | | | | | 1225.531 | 1323.125 |
| | | | | | | | | | | | 4764.143 | | | | | 1313.762 | 1866.5 |
| | | | | | | | | | | | | | | | | 1202.5 | 1477.188 |
| | | | | | | | | | | | | | | | | 1371.2 | 1291.762 |
| | | | | | | | | | | | | | | | | 1216.294 | 1498.143 |
| | | | | | | | | | | | | | | | | 1565.188 | 1173.273 |
| | | | | | | | | | | | | | | | | 1270.935 | 1726 |
| | | | | | | | | | | | | | | | | 1472.75 | 985.417 |
| | | | | | | | | | | | | | | | | 1525.25 | 1188.188 |
| | | | | | | | | | | | | | | | | 1268.3 | 1180.75 |
| | | | | | | | | | | | | | | | | 1268.154 | 1473.375 |
| | | | | | | | | | | | | | | | | 1206.25 | 1309.562 |
| | | | | | | | | | | | | | | | | 1283.238 | 1666.571 |
| | | | | | | | | | | | | | | | | | 1501.905 |
| | | | | | | | | | | | | | | | | | 1053.2 |
| | | | | | | | | | | | | | | | | | 1222.8 |
| | | | | | | | | | | | | | | | | | 1024.792 |
| | | | | | | | | | | | | | | | | | 1065.125 |
| | | | | | | | | | | | | | | | | | 999.75 |
| | | | | | | | | | | | | | | | | | 1410.192 |
| | | | | | | | | | | | | | | | | | 1422.81 |
| | | | | | | | | | | | | | | | | | 1222.375 |
| | | | | | | | | | | | | | | | | | 1319 |
| | | | | | | | | | | | | | | | | | 1138.812 |
| | | | | | | | | | | | | | | | | | 1279.688 |
| | | | | | | | | | | | | | | | | | 1124.19 |
| | | | | | | | | | | | | | | | | | 1157.962 |
| | | | | | | | | | | | | | | | | | 1434.714 |
| | | | | | | | | | | | | | | | | | 1457.095 |
| | | | | | | | | | | | | | | | | | 1126.654 |
| | | | | | | | | | | | | | | | | | 1363 |
| | | | | | | | | | | | | | | | | | 1428.769 |
| | | | | | | | | | | | | | | | | | 1152.625 |
| | | | | | | | | | | | | | | | | | 1098.381 |
| | | | | | | | | | | | | | | | | | 1226.097 |
| | | | | | | | | | | | | | | | | | 1468.417 |
| | | | | | | | | | | | | | | | | | 1261.269 |
| | | | | | | | | | | | | | | | | | 1357.625 |
| | | | | | | | | | | | | | | | | | 959.524 |
| | | | | | | | | | | | | | | | | | 1192.125 |
| | | | | | | | | | | | | | | | | | 1382 |
| | | | | | | | | | | | | | | | | | 1737.81 |
| | | | | | | | | | | | | | | | | | 1326.381 |
| | | | | | | | | | | | | | | | | | 1808.143 |
| | | | | | | | | | | | | | | | | | 1395.476 |
| | | | | | | | | | | | | | | | | | 1529 |
| | | | | | | | | | | | | | | | | | 1200.692 |

# Supplementary Figure 5 – Replication timing (NT and Chronic)

| | NT | | NT | | NT | | 0h | | 0h | | 0h | | 5h | | 5h | | 5h | |
|---|---|---|---|---|---|---|---|---|---|---|---|---|---|---|---|---|---|---|
| Chromosome Length | $^{myc}$ATR | | $^{myc}ATR^{\Delta C\ -/-}$ (Cl1) | | $^{myc}ATR^{\Delta C\ -/-}$ (Cl8) | | $^{myc}$ATR | | $^{myc}ATR^{\Delta C\ -/-}$ (Cl1) | | $^{myc}ATR^{\Delta C\ -/-}$ (Cl8) | | $^{myc}$ATR | | $^{myc}ATR^{\Delta C\ -/-}$ (Cl1) | | $^{myc}ATR^{\Delta C\ -/-}$ (Cl8) | |
| | R1 | R2 | R1 | R2 | R1 | R2 | R1 | R2 | R1 | R2 | R1 | R2 | R1 | R2 | R1 | R2 | R1 | R2 |
| 0.355712 | 0.045753 | -0.10431 | -0.06849 | -0.05772 | -0.07001 | -0.18404 | 0.411385 | -0.08011 | -0.18792 | -0.13446 | -0.2091 | -0.17116 | -0.25888 | -0.21478 | -0.31226 | -0.29363 | -0.28901 | -0.26668 |
| 0.384502 | 0.197911 | -0.0467 | 0.193601 | 0.209724 | 0.147083 | -0.11868 | 0.496223 | 0.043249 | -0.15195 | -0.06772 | -0.17614 | -0.14082 | -0.22352 | -0.2184 | -0.30001 | -0.28632 | -0.29647 | -0.26627 |
| 0.472852 | 0.179271 | 0.169543 | 0.123747 | 0.110214 | 0.126746 | 0.120933 | 0.366174 | 0.23912 | 0.146746 | 0.20646 | 0.127106 | 0.127103 | 0.05652 | 0.076764 | 0.045317 | 0.052298 | 0.042607 | 0.081284 |
| 0.465823 | 0.030134 | 0.155519 | 0.015057 | -0.03566 | -0.01184 | 0.15597 | 0.366992 | 0.232611 | 0.192135 | 0.25617 | 0.215928 | 0.198457 | 0.037966 | 0.066593 | 0.050423 | 0.07245 | 0.119069 | 0.124242 |
| 0.516869 | 0.227368 | -0.09373 | 0.172388 | 0.154691 | 0.101727 | -0.13668 | 0.47922 | -0.08139 | -0.14435 | -0.06999 | -0.14328 | -0.14577 | -0.22998 | -0.21469 | -0.22422 | -0.21244 | -0.24327 | -0.1656 |
| 0.596352 | 0.238565 | 0.050818 | 0.098053 | 0.095916 | 0.069969 | -0.05216 | 0.433283 | 0.04854 | -0.01727 | 0.0192 | -0.02875 | -0.00798 | -0.05056 | -0.06819 | -0.10409 | -0.09481 | -0.10148 | -0.07536 |
| 0.57496 | -0.1506 | -0.04001 | -0.6771 | -0.6807 | -0.56043 | -0.27023 | 0.092144 | 0.004575 | -0.79744 | -0.88309 | -0.65756 | -0.63675 | -0.02018 | 0.002164 | -0.83319 | -0.93565 | -0.73095 | -0.7502 |
| 0.573434 | 0.21402 | 0.117594 | 0.228491 | 0.229382 | 0.195514 | 0.098922 | 0.374161 | 0.170738 | 0.087199 | 0.149172 | 0.08213 | 0.092283 | 0.032322 | 0.014755 | -0.02663 | -0.0122 | 0.019158 | 0.022753 |
| 0.570865 | 0.295283 | 0.279495 | 0.278755 | 0.247887 | 0.259183 | 0.235908 | 0.273812 | 0.263082 | 0.168911 | 0.223175 | 0.168345 | 0.19327 | 0.138729 | 0.118672 | 0.141954 | 0.155965 | 0.189671 | 0.171275 |
| 0.582573 | 0.01816 | 0.082451 | -0.01703 | 0.021387 | -0.01188 | 0.044893 | 0.112938 | 0.061637 | 0.031092 | 0.059103 | 0.036216 | 0.06636 | -0.0088 | -0.030324 | -0.00389 | 0.020873 | -0.00958 | 0.00583 |
| 0.675346 | 0.334501 | 0.074892 | -0.02782 | -0.0877 | -0.24429 | -0.26537 | 0.115775 | -9.7E-05 | -0.43931 | -0.49763 | -0.34656 | -0.35925 | 0.003385 | -0.02279 | -0.45936 | -0.51793 | -0.40778 | -0.4551 |
| 0.654595 | 0.224373 | 0.02668 | 0.213602 | 0.188676 | 0.19741 | 0.031249 | 0.302073 | 0.07131 | 0.022711 | 0.067451 | 0.015427 | 0.010568 | -0.03773 | -0.03681 | -0.05539 | -0.03768 | -0.04283 | -0.01965 |
| 0.622644 | 0.246672 | 0.251876 | 0.085326 | 0.051824 | 0.152496 | 0.168552 | 0.122011 | 0.275882 | 0.055227 | 0.040816 | 0.071988 | 0.084314 | 0.206615 | 0.214573 | -0.02702 | -0.0451 | 0.035806 | 0.01404 |
| 0.629517 | 0.220515 | -0.00585 | 0.327517 | 0.215969 | 0.258801 | 0.011739 | 0.352317 | 0.077657 | -0.04489 | -0.00194 | -0.05401 | -0.04765 | -0.07689 | -0.10136 | -0.11199 | -0.12123 | -0.08492 | -0.08316 |
| 0.714651 | 0.202859 | -0.02106 | 0.070142 | 0.06372 | 0.072529 | -0.06819 | 0.358763 | -0.00973 | -0.10755 | -0.07889 | -0.10903 | -0.07418 | -0.08403 | -0.10501 | -0.17824 | -0.16684 | -0.12424 | -0.11045 |
| 0.684829 | 0.206149 | 0.076941 | 0.079167 | 0.08317 | 0.100813 | 0.008919 | 0.248226 | -0.00076 | 0.028674 | 0.080833 | 0.030515 | 0.040338 | -0.05015 | -0.05383 | -0.01899 | -0.03448 | -0.03224 | 0.045494 |
| 0.739748 | 0.017792 | 0.23302 | 0.013362 | 0.048189 | 0.023184 | 0.200164 | -0.09944 | 0.165667 | 0.101464 | 0.101045 | 0.070154 | 0.065301 | 0.188314 | 0.158304 | 0.106589 | 0.105314 | 0.026654 | 0.046922 |
| 0.702208 | -0.04998 | 0.104584 | -0.00541 | 0.034872 | 0.038759 | 0.109051 | -0.09999 | 0.154883 | 0.148096 | 0.178048 | 0.132037 | 0.131917 | 0.105 | 0.115975 | 0.134584 | 0.137646 | 0.112298 | 0.117519 |
| 0.742537 | 0.200892 | 0.094504 | 0.239143 | 0.23189 | 0.215377 | 0.069008 | 0.21271 | 0.118211 | 0.106725 | 0.130211 | 0.08763 | 0.092676 | -0.00718 | 0.031114 | 0.058817 | 0.070626 | 0.062891 | 0.089108 |
| 0.772972 | 0.162512 | 0.045439 | 0.143333 | 0.140984 | 0.106016 | 0.039308 | 0.225261 | 0.017812 | 0.022359 | 0.021645 | 0.004434 | 0.00019 | -0.01445 | -0.04799 | -0.03992 | -0.02426 | -0.04958 | -0.023 |
| 0.716602 | 0.039216 | 0.158862 | 0.132223 | 0.168119 | 0.133429 | 0.196844 | 0.079128 | 0.203458 | 0.199789 | 0.221509 | 0.196598 | 0.208035 | 0.145746 | 0.177585 | 0.158324 | 0.187928 | 0.171361 | 0.19617 |
| 0.772565 | 0.203839 | 0.091087 | -0.04846 | -0.06141 | -0.0537 | -0.03311 | 0.135245 | 0.044828 | -0.09907 | -0.13319 | -0.04505 | -0.05155 | 0.049326 | 0.029372 | -0.11141 | -0.1417 | -0.03985 | -0.06912 |
| 0.84095 | 0.197878 | -0.00068 | 0.205273 | 0.180349 | 0.174545 | 0.010083 | 0.290631 | 0.052174 | 0.011755 | 0.061965 | 0.03508 | 0.032525 | -0.00322 | -0.06278 | -0.01775 | 0.025433 | -0.00125 | 0.020356 |
| 0.912845 | 0.117786 | 0.017307 | 0.099548 | 0.092593 | 0.172405 | 0.03264 | 0.145821 | 0.039724 | 0.034615 | 0.043391 | 0.003344 | 0.010992 | 0.01251 | 0.005435 | 0.00162 | 0.011865 | 0.012752 | 0.017198 |
| 1.09154 | 0.106266 | -0.06749 | 0.183211 | 0.158471 | 0.185881 | 0.010354 | 0.119389 | -0.0434 | 0.016972 | 0.05293 | 0.009188 | 0.027901 | -0.01474 | -0.04836 | 0.013945 | 0.033749 | 0.031912 | 0.045537 |
| 1.130424 | -0.15238 | -0.13983 | -0.11086 | -0.09965 | -0.06887 | -0.10296 | -0.12181 | -0.14293 | -0.06314 | -0.08855 | -0.06253 | -0.0719 | -0.13642 | -0.14698 | -0.08919 | -0.07518 | -0.08681 | -0.05186 |
| 1.160104 | 0.049797 | -0.02717 | 0.119168 | 0.129497 | 0.104452 | 0.026079 | -0.04783 | -0.06707 | -0.01755 | -0.01548 | -0.03344 | -0.03253 | -0.02061 | -0.02455 | -0.00317 | 0.006902 | -0.01226 | -0.01355 |
| 1.212663 | -0.04899 | 0.042351 | -0.00727 | 0.006311 | 0.015004 | 0.087457 | -0.21013 | 0.012157 | 0.073669 | 0.048118 | 0.053347 | 0.042513 | 0.043119 | 0.060165 | 0.109085 | 0.099715 | 0.081427 | 0.088133 |
| 1.403434 | 0.016414 | -0.06428 | 0.141002 | 0.122681 | 0.092978 | 0.017307 | -0.06004 | -0.09456 | 0.009733 | -0.01618 | 0.004868 | 0.009437 | -0.01025 | -0.02967 | 0.053705 | 0.07336 | 0.054492 | 0.026225 |
| 1.484328 | -0.4757 | -0.04503 | -0.8505 | -0.85498 | -0.85208 | -0.28788 | -0.2741 | -0.03972 | -0.22714 | -0.30936 | -0.20459 | -0.22451 | 0.045621 | 0.061076 | -0.12214 | -0.21811 | -0.11086 | -0.22641 |
| 1.604637 | -0.15876 | -0.07581 | -0.1045 | -0.09764 | -0.0963 | -0.04269 | -0.29094 | -0.18962 | -0.04132 | -0.05517 | -0.03894 | -0.05135 | -0.04367 | -0.0178 | 0.020415 | 0.041071 | -0.00387 | 0.009208 |
| 1.583653 | -0.04865 | -0.13902 | 0.011253 | 0.014679 | 0.055847 | -0.08136 | -0.23264 | -0.17337 | -0.01174 | -0.02904 | -0.01974 | -0.04414 | -0.07251 | -0.05922 | 0.068925 | 0.049471 | 0.019801 | 0.023639 |
| 1.866748 | -0.09784 | -0.05603 | 0.007131 | 0.025719 | 0.015158 | 0.015588 | -0.29222 | -0.08279 | 0.050479 | 0.02605 | 0.03382 | 0.026066 | 0.0157 | 0.011446 | 0.113276 | 0.130626 | 0.090116 | 0.083908 |
| 2.090474 | -0.10354 | -0.07617 | 0.026762 | 0.050493 | 0.027464 | -0.01271 | -0.23255 | -0.0269 | 0.094949 | 0.060439 | 0.108737 | 0.095933 | 0.010505 | 0.008872 | 0.164672 | 0.138807 | 0.114285 | 0.10264 |
| 2.682151 | -0.18449 | -0.08569 | -0.01371 | 0.00726 | 0.0000134 | 0.04044 | -0.2987 | -0.065 | 0.077104 | 0.059417 | 0.067072 | 0.068364 | 0.027247 | 0.044725 | 0.143726 | 0.156453 | 0.135355 | 0.116394 |

# Script used for the generation of Metaplots

```
import pandas as pd
import numpy as np
import matplotlib.pyplot as plt

def read_bed(file_path):
    return pd.read_csv(file_path, sep='\t', header=None, names=['chrom', 'start', 'end'])

def read_bedgraph(file_path):
    return pd.read_csv(file_path, sep='\t', header=None, names=['chrom', 'start', 'end', 'mean_score', 'std_score'])

def extend_and_scale_regions(bed_df, upstream=150000, downstream=150000, scale_length=20000):
    extended_regions = []
    for _, row in bed_df.iterrows():
        chrom = row['chrom']
        center = (row['start'] + row['end']) // 2
        scaled_start = center - scale_length // 2
        scaled_end = center + scale_length // 2
        extended_start = scaled_start - upstream
        extended_end = scaled_end + downstream
        extended_regions.append((chrom, extended_start, extended_end))
    return pd.DataFrame(extended_regions, columns=['chrom', 'start', 'end'])

def bin_scores(extended_regions, bedgraph_df, bin_size=500):
    region_length = 20000 + 2 * 150000  # Total length
    num_bins = region_length // bin_size
    all_bins = np.full((len(extended_regions), num_bins, 2), np.nan)  # Initialize with NaN

    for i, region in extended_regions.iterrows():
        for j, start in enumerate(range(int(region['start']), int(region['end']), bin_size)):
            end = start + bin_size
            bin_data = bedgraph_df[(bedgraph_df['chrom'] == region['chrom']) &
                                   (bedgraph_df['start'] < end) &
                                   (bedgraph_df['end'] > start)]
            if not bin_data.empty:
                mean_score = bin_data['mean_score'].mean()
                std_score = bin_data['std_score'].mean()
                all_bins[i, j, 0] = mean_score
                all_bins[i, j, 1] = std_score

    avg_scores = np.nanmean(all_bins, axis=0)
    return avg_scores

def plot_metaplot(avg_scores_list, bin_size, labels):
    region_length = 20000 + 2 * 150000
    positions = np.linspace(-150000, 150000 + 20000, len(avg_scores_list[0]))

    plt.figure(figsize=(9, 8))

    # Plot each signal
    colors = ['black', 'royalblue', 'lightcoral', 'firebrick']
    for idx, avg_scores in enumerate(avg_scores_list):
        mean_scores = avg_scores[:, 0]
        std_scores = avg_scores[:, 1]
        plt.plot(positions, mean_scores, label=labels[idx], color=colors[idx], linewidth=2)
        plt.fill_between(positions, mean_scores - std_scores, mean_scores + std_scores, color=colors[idx], alpha=0.2)

    plt.axvline(x=0, color='white', linestyle='--', linewidth=0.5, label='Region Center')
    plt.axvline(x=20000, color='white', linestyle='--', linewidth=0.5, label='Region End')
    plt.axhline(y=0, color='gray', linestyle='--', linewidth=1.5, label='y=0')
    plt.title(Title')
    plt.xlabel('Position (bp)')
    plt.ylabel('Score')

    # Adjust y-axis limits (update min and max as needed)
    plt.ylim(-0.7, 1.2)  # Example: set y-axis limits from -2 to 2

    plt.legend(loc='best')  # Adjust legend location
    plt.grid(False)  # Disable grid
    plt.tight_layout()  # Adjust layout to prevent clipping
    plt.savefig('metaplot.png', dpi=200)
    plt.show()

# Paths to input files
bed_file_path = *.bed file'
bedgraph_files = [
*.bedgraph]

# Read the BED file
bed_df = read_bed(bed_file_path)

# Extend and scale the regions
extended_regions = extend_and_scale_regions(bed_df, upstream=150000, downstream=150000, scale_length=20000)

# Process each BEDGRAPH file and compute scores
avg_scores_list = []
for bedgraph_file_path in bedgraph_files:
    bedgraph_df = read_bedgraph(bedgraph_file_path)
    avg_scores = bin_scores(extended_regions, bedgraph_df, bin_size=500)
    avg_scores_list.append(avg_scores)

# Plot the metaplot
plot_metaplot(avg_scores_list, 500, labels=['Signal 1', 'Signal 2', 'Signal 3', 'Signal 4'])
```
