# Supplementary material for: ATR, a DNA damage kinase, modulates DNA replication timing in Leishmania major
Source: PLoS Genet. 2025 Nov 24;21(11):e1011899. doi: 10.1371/journal.pgen.1011899 (PMC12677790; doi:10.1371/journal.pgen.1011899)

Supporting information:
Supplemental Dataset – Da Silva et al.

Western blots and FACS data from this study.
(labels are shown in corresponding figures in the manuscript

Figure 1C
R2

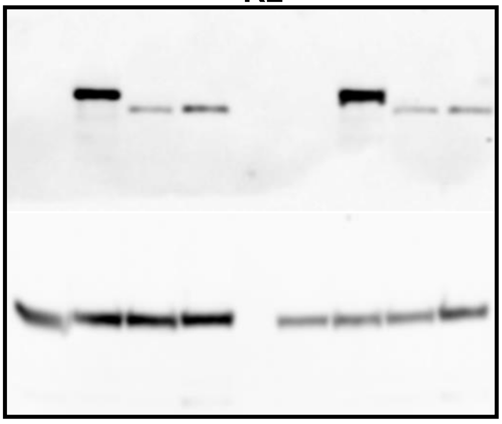

Figure 3C –Cell cycle profile

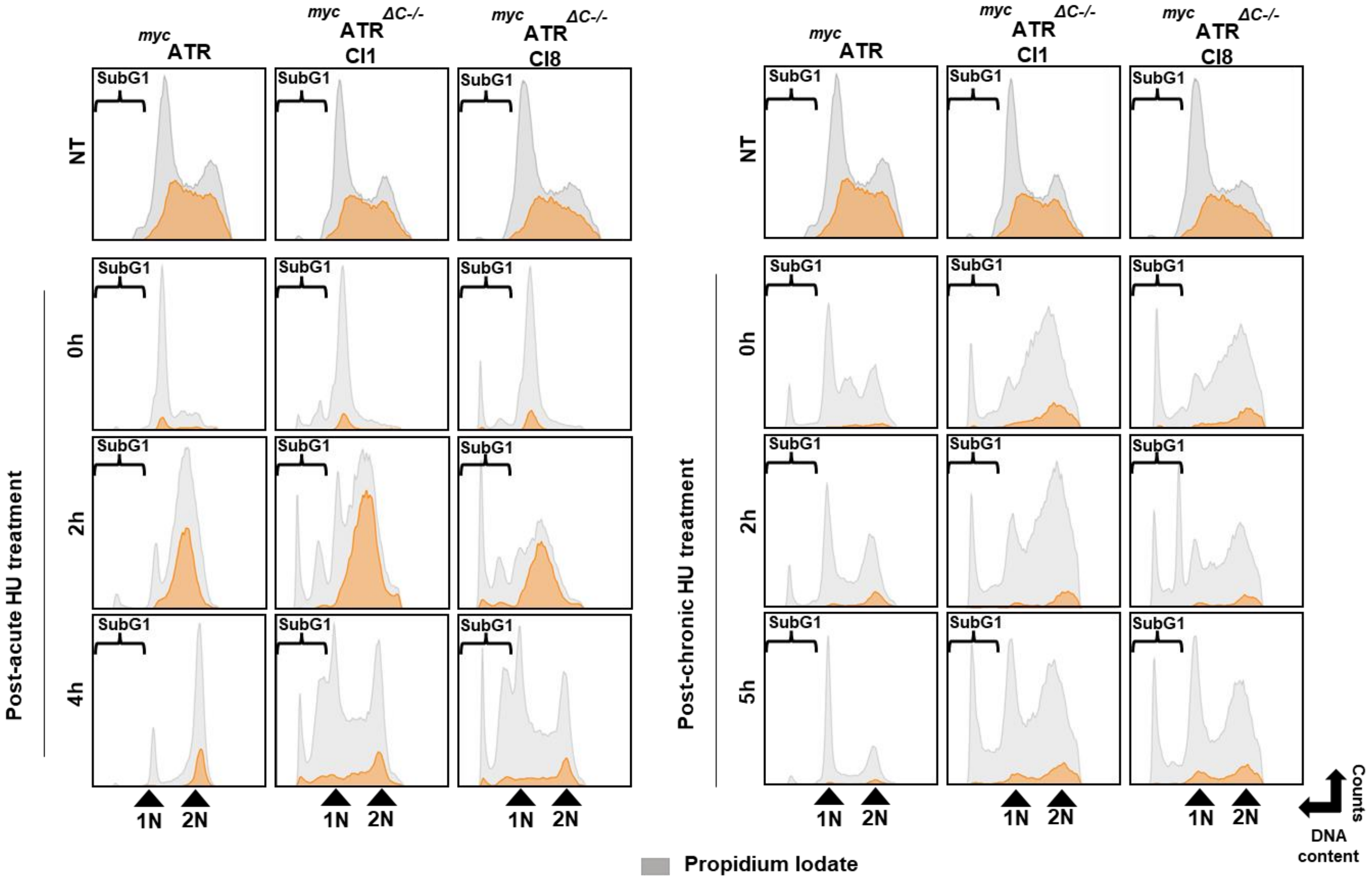

Figure 4A
R2

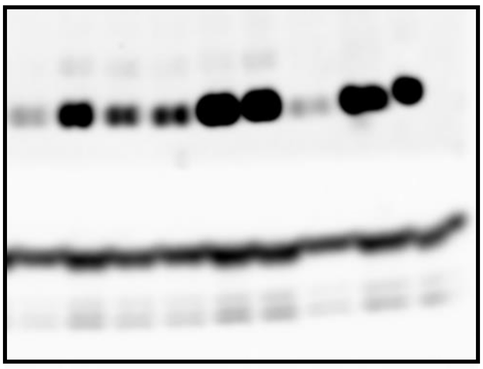

Figure 4B
R2

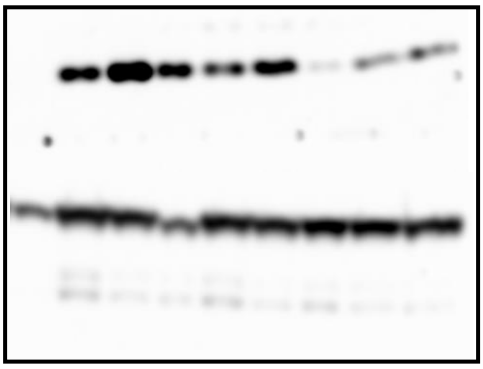

Figure 4A
R3

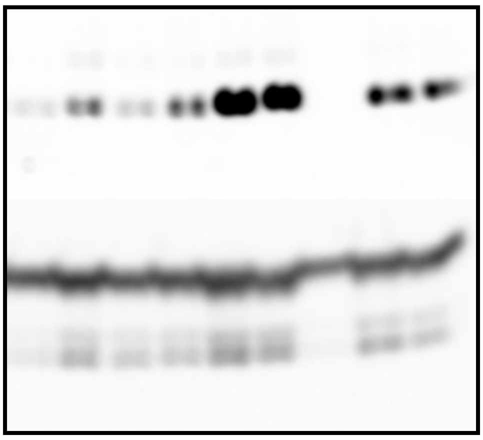

Figure 4B
R3

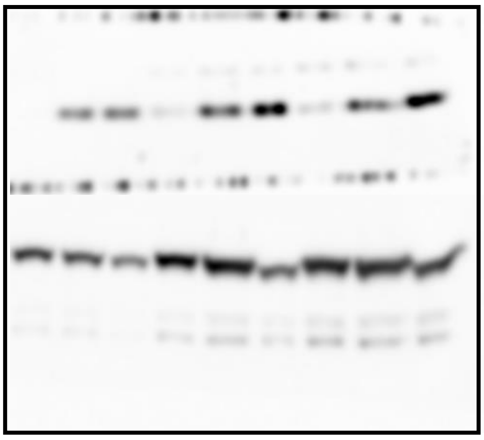

Supplement: S2 File — (PDF) [file pgen.1011899.s015.pdf]
