# Supplementary material for: ATR, a DNA damage kinase, modulates DNA replication timing in Leishmania major
Source: PLoS Genet. 2025 Nov 24;21(11):e1011899. doi: 10.1371/journal.pgen.1011899 (PMC12677790; doi:10.1371/journal.pgen.1011899)
Supplement: Attachment — Submitted filename: Response to reviewers.pdf [file pgen.1011899.s019.pdf]

**ATR, a DNA damage kinase, modulates DNA replication timing in *Leishmania major*.**

Corresponding Authors: Professor Richard McCulloch and Professor Luiz R. O. Tosi

**We thank all reviewers for their helpful comments and very useful suggestions. Detailed responses to each reviewer's comments are provided in red text, and below is a summary of the new data and analysis we have added in response to the comments:**

**Fig. 1. Added a schematic of the domains that were deleted from the C-terminus (A). Added statistical analysis (C): unpaired t-test. Added a legend for the small panel in (D).**
**Fig. 2. Analysed (B) with unpaired t-test.**
**Fig. 3. Analysed (B) and (E) with unpaired t-test.**
**Fig. 4. Analysed (C-D) with unpaired t-test. In (E), modified figure size and the image colours for better visualization. In (F), modified the colours for better visualization.**
**Fig. 5. (B) Changed the colors of the heatmap (one for each sample), displaying more chromosomes and showing the chromosome scale at the top. (D – E) Analysed with unpaired t-test.**
**Fig. 6. (A, B and E) Added shuffled controls for each metaplot. (C) Changed the colors of the heatmap (one for each sample).**
**Fig. S3. Analysed with unpaired t-test.**
**Fig. S4. Added the MFAseq signal of each replicate and the merged output on the heatmap; colours changed for clarity.**
**Fig. S6. This figure was split (A-B) and the rest of the data turned into Fig. S9, for better comprehension of the manuscript.**
**Fig. S7. Changed the colours of the heatmap (one for each sample).**
**Fig. S8. This figure is new and was created to show the individual MFA-seq signal of each replicate, of each sample in the two time points after chronic HU treatment.**
**Table S1. This table is new and was added to show the location and length of ERR, SSR/SIDER1/SIDER2 and SIDER1/SIDER2 elements.**

**REVIEWER COMMENTS**

Reviewer #1 (Remarks to the Author):

Comments for the Author:

This manuscript characterises the ATR checkpoint kinase homologue in the kinetoplastid parasite Leishmania. DNA replication, DNA damage checkpoints and DNA repair in this species is arguably a topic of particular interest because Leishmania has a highly plastic genome, and may have an unusual programme of genome replication timing.

The checkpoint kinases have been examined before in Leishmania using chemical inhibitors (e.g. PMID: 35059327, PMID: 30265735) and a monoallelic rather than diallelic ATR deletion (ref 47 preprint); plus the replication timing programme has been examined before via MFAseq by these authors. Overall, this somewhat limits the novelty of this manuscript: a large number of papers from the same authors, piecing together slightly different approaches to ATR, could arguably have been published in a more streamlined manner. Nevertheless, some advances are made here. The innovations are essentially a) to make a partial knockout of the ATR gene (deleting the kinase domain of both alleles) rather than chemically inhibiting it or knocking out only 1 allele, and b) combining this knockout with replication experiments via MFAseq.

The data are thoroughly analysed (modulo comments below) and the conclusions are largely justified by the data. They show that in this early diverging parasite, ATR appears to act broadly as it does in other eukaryotes, protecting stressed replication forks and promoting their recovery, despite the lack of obvious homologues for some ATR partners. The weakness of the paper is primarily the inability to define exactly how ATR acts – it is promotion of inefficient origin firing, or promotion of replication restart, or both? This is due to the low resolution of MFAseq, which allows only broad, indirect conclusions about DNA replication. Since higher-resolution techniques are available, e.g. via DNAscent technology (already used by this group in Leishmania) and DNA combing (used by other groups in the same parasite), it is not clear why MFAseq with its acknowledged weaknesses was chosen. A more succinct and direct manuscript could perhaps have been written if the data were more directly informative. An origin map via ORC ChIP could also greatly enhance the clarity about what is going on in Leishmania DNA replication. These are probably not, however, practical experiments to do or re-do at the revision stage.

When these experiments were being conducted, we had not established DNAscent, sadly, and so it was not feasible to use it. We do intend to use the ATR mutant to ask if the DNAscent signals we see beyond the single MFAseq peak in each chromosome differ from wild type, but this is an extensive experiment bioinformatically, given the huge numbers of stochastic signals we see. We agree that ORC ChIP would help, but for reasons that yet remain unclear, epitope tagging ORC in *Leishmania* is detrimental. For both questions, further work is needed, as the reviewer indicates.

Specific comments:

At L222 'Quantification of the signal intensity confirmed a significant increase in the HU-treated mycATR.C-/- cells (Figure 3B), indicating that loss of ATR leads to the accumulation of ssDNA in L. major'. Strictly, this appears to be overinterpreted: there are other

explanations for extra BrdU staining that aren't addressed. For example, the mutant parasites could have replicated more slowly, from different oris, or just overall more, during the IdU pulse – leading to more baseline IdU, although the same proportion of ssDNA.

We respectfully disagree. The results from Figure S1B show that in normal conditions there is no difference in the proportion of cells that incorporate IdU when comparing mycATRΔC-/- and mycATR cells, suggesting that the growth effect after ATR mutation is not due to slower DNA replication. The data showing increased ssDNA is via detection with anti-BrdU antiserum (Fig.3B), and all we claim is that the mutants display greater signal in these experiments, which differs from the absence of baseline IdU incorporation (Fig.S1B). This signal could, of course, be ssDNA at normally silent origins activated by loss of ATR, but we have no evidence for such a connection.

L330 'Second, this broader distribution of MFA-seq signal was not entirely consistent between the two mycATR.C-/- clones, suggesting stochastic variation in the changed DNA replication program.' Looking at Fig S4, the two clones are actually radically different, with cl8 resembling parental on many chromosomes, whereas cl1 does not. In general, the marked differences between the two clones – which are seen in several figures, not just here, e.g. in differing baseline ssDNA levels in Fig 3B – are neglected in the discussion. Why are 2 supposedly identical clones so different in phenotype?

We agree that this is perplexing but sadly we do not have an explanation. One possibility is unexplored adaptation to the loss of ATR; indeed, in this regard, we also recovered a mutant clone that displayed a large change in ploidy, but did not follow this up, as it was not seen in clones cl8 or cl1 (see FACS plots below).

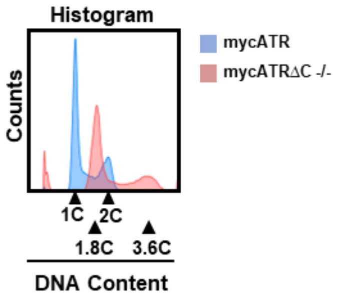


Nonetheless, it is important to note that both clones provide evidence for the importance of ATR during replication stress: both are sensitive to HU, accumulate ssDNA and yH2A after HU treatment, show cell cycle defects, and alter the DNA replication programme. In terms of the differences in MFA-seq profiles between the clones, this may reflect stochasticity in the location of the signals we see after HU treatment, and we have added this text:

'One explanation for these new MFA-seq loci is that they represent normally suppressed origins, which are activated in the absence of ATR with and without excess replication stress caused by HU, *and randomness in the loci at which they are activated could explain the differences in MFA-seq profiles observed between the mutant clones*.'

L365: 'the chromosome-size-dependent replication timing profile observed in untreated cells was maintained: smaller chromosomes had higher average MFA-seq signals compared with larger chromosomes…' In general, I think the authors might acknowledge the fact that the somewhat mysterious 'later replication timing' of larger chromosomes could simply be due to them having a greater number of stochastically firing origins, as presumably required to get larger chromosomes replicated within a single S-phase. Pre-printed data from DNAscent

(same authors) does, I believe, suggest this, and provides a much more conventional explanation for how the small and large chromosomes replicate in these parasites. After HU, larger chromosomes having higher MFAseq could also be consistent with stress simply leading to more stochastic ori firing, in an attempt to rescue replication? Overall, in the discussion (L484) this is presented as 'our data also reveals a novel role for ATR in controlling the unusual DNA replication program of L. major' – but I'm not sure this isn't really just a side-effect of a 'normal' role for ATR.

We accept that this is possible, and have discussed that the data may be consistent with 'normal' roles documented in other eukaryotes: e.g. 'our data also reveals a crucial role for ATR in controlling the unusual DNA replication program of *L. major*'; 'Such a view would align with known functions of ATR in other eukaryotes, where licensed but not activated origins are controlled by ATR signalling (85), which acts as a negative regulator of origin firing and prevents inappropriate activation of origin firing in unperturbed cells (86)'.

However, we also discuss other possibilities, given the unusual nature of DNA replication timing in *Leishmania*. It is important to note that there is no evidence that the greater number of 'origins' detected by DNAscent in larger chromosomes are conventional 'dormant' origins: these are clearly seen in the absence of replication stress, and we have no evidence that they reflect locations of ORC binding (in fact, this seems unlikely, as they are found throughout the multigene transcription units, where ORC would impact on RNA Polymerase movement).

In the final section re. genome mutations in ATR mutants, it seems contradictory that ATR mutants get fewer SNPs, indels etc. but are supposed to fire more oris, since it also stated 'loci where DNA replication initiates are prone to accumulate SNPs'??

We agree, this is surprising, but we improved our analysis (Fig. S10), which confirms that mycATR cells accumulate more SNPs than mycATRΔC-/- mutants, and that InDels arise more frequently in the latter. Why this is so, we can only speculate, and have limited such speculation in the absence of any explanatory data: in the discussion, we merely state: '..but it has been demonstrated that loci where DNA replication initiates are prone to accumulate SNPs (93,94) and, indeed, the *L. major* ATR mutant struggles to progress/re-start replication after replicative stress'. Nonetheless, one potential explanation is that in the presence of functional ATR replication restart is relatively efficient, whereas in the absence of ATR more mutagenic restart, such as by engaging translesion DNA Pols, is needed (PMID: 28753428, PMID: 23945592, PMID: 18594563).

L524 The discussion states: 'So far, there has been little evidence for 'dormant' origins in trypanosomatids'… this appears to neglect both the DNAscent and DNA combing data, which showed plenty of stochastically firing origins?

Thank you for your comment, but please see our response above: we have no evidence that the widespread stochastic 'origins' detected by DNAscent represent dormant origins, since they appear to be part of the normal programming of DNA replication in *L. major*. As regards DNA combing in *Leishmania*, we're afraid we cannot find any evidence that this has been applied after HU treatment and, as conducted to date, cannot be mapped to the genome, so provides scant evidence for dormant origins

In Fig 1, it would be useful to show how the kinase domain was defined.

The figure and the legend were modified to include what has been deleted. For how the kinase domain was defined, please see the responses to reviewer #3: “- The catalytic domain of LmjF.32.1460 is located at 2863-3135 aa according to the NCBI database. The authors deleted 2060-3135 aa and should comment on this.”

Fig 4E: the microscopy pictures are very small and resolution appears poor – can the nuclei and kinetoplastids really be confidently defined?

We agree, and Figure 4E has been improved: the area where the cell snapshots were taken was zoomed out to get a better resolution, and the colours were changed to have a better contrast between DNA content and cytoskeleton. N-K proportions were analysed considering individual cells in which the nuclei and kDNA staining could be clearly distinguished in each condition.

Fig 5: did the authors really wait 10 cycles after HU stress before doing the MFAseq? If so, why? Surely the next cycle would be the place to see acute effects upon replication timing?? Or did they only wait 10 cycles before sequencing for mutations? This is not clear.

Thank you for your comment. The purpose of 10 cycles was to observe the effect of ATR C-terminal deletion after prolonged growth of cells in low levels of replication stress. We have used a similar experimental set-up to observe genomic changes previously (PMCID: PMC7360064, PMCID: PMC11807225).

In Fig 5 there is a huge unexplained grey region with no apparent data in chr 8??

Thank you for your comment. The grey regions are areas with poor mapping quality, as described in PMID: 27477279. This information is now added to the figure legend. Also, we have now provided further examples of this analysis in Figure 5B.

Fig 5D and 5E appear essentially redundant?

We respectfully disagree. Both come from the same set of data, but both analyses are important to emphasize the increase in average of MFA-seq signal in the large chromosomes in the ATR mutant parasites. We modified figure 5D to show more data points.

Fig S4 legend refers to ?KKT1 but doesn’t define it, and SIDERs are mentioned but not apparently labelled

Corrected, thank you.

Fig S7 : Chr 1 and 36 are absent, and there is a (D) in the legend after (A) but no fig (D) is seen?

Thank you for your comment. Chromosome 1 was excluded because an amplification in copy number was found in our stationary control sample; the legend has been modified to state this. Chromosome 36 was added and is also displayed in Figure 6C.

Fig S8: is the data on CNVs at all quantifiable? Between the CNV changes both up and down, it does not look statistically significant.

Thank you for the suggestion. We used an Unpaired t-test to statistically evaluate the differences in CNV for each chromosome between the two mycATRΔC-/- clones and mycATR cells. The text and the figure were corrected accordingly.

Formatting comments:

L39 add comma after family

Corrected, thank you

L74 add comma after ultimately

Corrected, thank you

L80 accumulation?

It has been demonstrated that accumulation activates ATR (PMCID: PMC1847708).

L385 chromosomes (plural)

Corrected, thank you

At L407, Fig S7 appears before he latter half of Fig S6, which is confusing

Thank you, we divided Figure S6 (Figure S9) to accommodate Figures S7 and S8.

L409 correct 'MFAs-seq'

Corrected, thank you

L421: 'SIDER' appers but the abbreviation is not apparently defined?

'..interspersed degenerate retrotransposons (SIDERs), which are involved in posttranscriptional (SIDER1) and translational (SIDER2)..'

L430: kb not Kb

Corrected, thank you

L542: Fig 5A appears to be misreferenced (doesn't show this)

Corrected, thank you, the reference is Figure 6A and 6B

L529: suppresses

Corrected, thank you

Throughout the methods, check for failed italicisation of scientific names, use of u instead of mu for micro, lack of superscripts and subscripts, erratic capital letters, etc.

Corrected, thank you

L1040 survival

Corrected, thank you

L1076 '…I n each condition was calculated by the number of stained cells by the number of present in each sample' – sentence makes no sense?

Corrected, thank you

L1105: 'exponentially untreated growing cells' – makes no sense?

Corrected, thank you

L1116: delete in

Corrected, thank you

L1117: ‘Chromosomes were sub-classified according with their length’ – what were the cutoffs?

Thank you for your comment. 36 chromosomes: 3 classifications with 12 chromosomes in each, ranked by the length; figure legend improved for clarity.

L1125: ‘rp1 and rp2 represent the two replicas’ – these are nowhere on Fig 5

Thank you for your comment. The Figure 5F represent one replicate, both replicates are displayed at Figure S5.

Reviewer #2 (Remarks to the Author):

Comments for the Author:

This paper studies the role of ATR in the trypanosomatid Leishmania major, which harbors an unusual chromosomal organization with early-replicating small chromosomes and late-replicating large chromosomes. The main contribution is the generation and characterization of a CRISPR deletion of the ATR kinase domain. The authors show that loss of the kinase domain leads to slow growth, HU sensitivity, ssDNA accumulation and aberrant cell cycle progression and DNA damage accumulation under replication stress. The most novel result is that loss of ATR activity leads to advancement of replication of most large chromosomes, and even more profoundly, under replication stress the large chromosomes replicate earlier than the small ones. This advanced replication appears to be driven by new initiation sites. Aside from several concerns detailed below, I found this to be an interesting and well described manuscript.

Specific comments:

- Figure 3C: the sub-G1 population is unusual as it shows a distinct peak and in some cases, more than one peak. This potentially suggests the loss of specific parts of the genome. Can the authors further characterize these patterns?

Thank you for your comment. Indeed, the different HU treatments generate different sub-G1 populations in mycATRΔC-/- cells, and similar results were observed previously after conditional deletion of HUS1, which is likely to be part of the *L. major* ATR pathway (PMCID: PMC6294564). With the set of data we have, we cannot further characterize these populations. Whole-genome sequence cannot distinguish populations, and so the appropriate experiment would be FACS-sorting these populations and sequencing the genome individually with a spike-in control, which we suggest is beyond the scope of this study.

- Difference in MFA signal between the two clones- it would be useful to determine if this is a clone-specific difference or stochastic variation unrelated to the clones, in other words, if the same clone was profiled twice, would it also show the same variation? The legend of figure 5 suggests that two repetitions were done for each clone- can the consistency between them be quantified? Some of the differences between the clones seem concerning, for

instance on the right side of chromosome 08, the two clones almost appear to replicate in opposite ways. The presentation using a heatmap is also somewhat confusing as it is difficult to evaluate differences along the early-late continuum and also difficult to compare to the Friedlin data that is shown as a graph rather than a heatmap. What is the correlation of the MFA data between experimental repetitions, between clones, and to the Friedlin data? Overall, the centrality of these results warrant a clearer presentation and a more rigorous analysis of data quality.

Thank you for this suggestion. We have added the MFA-seq for each replicate to Figures S4 and S8, which shows there is experimental variation. We respectfully disagree in relation to the heatmap display, since we believe it is the clearest way to demonstrate the defects caused by mutation in ATR and after replicative stress. Nonetheless, we changed the colours of the heatmap for better visualisation of these areas, including for the WT strain.

Minor: Figure 5C (and others) indicate a positive R, however the regression slope is negative. Does R need to be negative? Or is this R squared?

Corrected, it is R-squared and was added to the legends, thank you.

- The CNV and SNP analysis appears less rigorous, less well described, and less well controlled and validated than other analyses. Overall, I find this section not very convincing. What was the allele frequency used to call SNPs (as an aside, the term SNPs is incorrect and should be replaced with SNV or mutations)? Is 10 generations sufficient to accumulate mutations in the absence of selection? Have the cells been cloned in any way? The called SNPs (and CNVs) could well be subclonal or even sequencing mistakes. Have any of them been validated (e.g., Sanger sequencing)? Overall, this section should be more rigorous, or removed.

We appreciate the reviewer's comments regarding the rigor and interpretation of our variant calling approach but respectfully disagree for two reasons. First, the approach we take should exclude random sequencing errors. We used FreeBayes (Garrison et al. 2012) for variant calling, which inherently considers allele frequency through a Bayesian inference model. Specifically, FreeBayes evaluates the probability of a variant existing at a site based on read-level support, using alternate allele counts (AO) and reference allele counts (RO). Although we did not manually set an explicit allele frequency threshold, FreeBayes's default behaviour requires sufficient alternate allele support to distinguish real variants from sequencing noise. For example, by default, FreeBayes expects at least 20% alternate allele fraction (internally used in probabilistic modelling). We set a minimum coverage threshold of 20 reads, meaning that, under default behaviour, a site would need approximately 4 alternate-supporting reads to be considered. This means that very low-frequency variants, including possible sequencing errors, are expected to pass this threshold and are therefore excluded prior to downstream filtering. Second, we have used a similar experimental set-up previously and observe changes in the same locations, showing that these are not random errors (PMCID: PMC7360064, PMCID: PMC11807225); the same approach guided our choice of 10 passages as being enough to observe variability between mutants and WT cells.

In terms of nomenclature, we changed SNPs to SNVs.

Reviewer #3 (Remarks to the Author):

Comments for the Author:

The authors made a study of the ATR protein of L. major. They failed to make a complete knockout of the ATR gene, but they tagged this gene at the N-terminus. However, the authors generated ATR mutants that lacked the predicted kinase domain at the C-terminus, and they analyzed these mutants and challenged them with hydroxyurea treatments. They found that the deletion of the C-terminus reduced the presence of the muted ATR protein in the nuclei. Mutants with deleted C-terminus showed aberrant cell cycle progression, accumulation of ssDNA in the nuclei and increased levels of yH2A, indicating increased levels of DNA damage. MFA-seq analysis was performed and the results showed that after ATR C-terminal deletion there are changes in the timing of DNA replication initiation. The authors also showed that deletion of the ATR C-terminus leads to a reduced accumulation of mutations, suggesting that ATR promotes chromosomal instability. The text is generally well written and understandable, but there are some statements that could be improved.

This study is useful for understanding the complex biology of this unicellular eukaryote of medical and veterinary interest. This work is also useful for understanding the processes of DNA replication stress and DNA damage response in this parasite. However, there are several deficiencies in the study that must be addressed to make it suitable for publication.

Major

- The statement that 'only a single locus per chromosome is consistently detected as the primary site of DNA replication initiation as detected by MFA-seq and DNAscent' is often repeated in the text. However, the MFA-seq methodology used in this study does not have the resolution of origin mapping to make this claim. It may be that there are many sites of DNA replication initiation within the large region that the authors refer to as the primary site of DNA replication initiation. It would be beneficial to consider moderating the interpretation of MFA-seq results to ensure a balanced and nuanced understanding of the findings.

Even more, it would be nice to discuss how and why the origins detected by MFA-seq in tagged mycATR differ from those detected in the WT strain that were previously published, saying that there is only one origin per chromosome in Leishmania (PMCID: PMC4612428). It seems that MFA-seq now detects more than one origin per chromosome even in the L. major WT strain (Figure S4).

We appreciate this comment, and agree that MFAseq may fail to detect lower efficiency origins: we have always made that clear (PMID: 26481451; PMID: 32897188), and in a recent preprint describe very abundant replication initiation signals beyond the single centromeric MF-seq peak (but even here, there is clear concentration of early S-phase replication initiation activity at this locus: (https://www.biorxiv.org/content/10.1101/2024.11.14.623610v1). Nonetheless, here we are reporting on DNA replication using MFAseq and feel it necessary to make clear what is normally seen in WT cells. To try and clarify this, we have amended the text to use the term "Early Replication Region" when referring to pre-eminent locus detected by MFAseq in each chromosome and SSR-ORI to refer to the switch strand region associated with it.

The MFAseq signal is WT cells reported here is unchanged from previous reports (Fig.S4), but we agree that the signal in mycATR cells appears less focused around the SSR-Ori in some chromosomes; though this modification of ATR does not alter growth of WT *L. major*,

a subtle change in replication detected by MFAseq may suggest some loss of ATR function. Nonetheless, it is important to note that mycATR cells do not show a change in the overall replication program (Fig.5C), and changes in MFAseq profiles are much different in mycATRΔC-/- mutants, which do show a change in replication program.

- It would be very useful to include a supplementary table with the exact genomic locations of the detected MFA peaks in WT, mycATR and mycATRΔC-/- mutants. The chromosome sizes and lengths of the MFA peaks should also be included in this table. Any statements in the main text about relationships between broader or narrower MFA peaks and chromosome lengths should also be correlated with this supplementary table. The exact positions of the 36 ERRs should also be provided. Otherwise, this section of the text may prove challenging to comprehend.

Thank you for this suggestion. A table (Table S1) was added with the location and length of ERR, SSR/SIDER1/SIDER2 and SIDER1/SIDER2 loci and referred at the text accordingly.

- The authors should further discuss the choice of analyzing DNA replication in chronic HU. According to Figure 3C-E, DNA replication is perturbed but recovered after acute conditions, whereas DNA replication seems to be blocked after acute HU treatment (Figure 3C) and the observed minority of cells labelled with EdU could only be cells where DNA repair occurs (less than 5% of cells labelled with EdU in chronic HU condition according to Figure 3E). Furthermore, as shown in Figure 1E, 0.5 mM HU is lethal even for the mycATR strain after 4 days. This concentration is highly toxic and 5 mM used in acute condition is even more toxic. It would be useful if the authors could comment on this.

Thank you for your suggestion: there were a mistake in the text, and this has been corrected in this new version (L333-338). The selection of HU chronic treatment was based on the results describe in Figure 3, where the effects on mycATRΔC-/- cells relative to mycATR were more clearly seen than the acute treatment. Here is a breakdown of the findings:

First, in Figure 3C, chronic treatment (0h): the profile of mycATRΔC-/- cells is very different than mycATR cells, showing an accumulation of cells in middle/late S-phase and remaining almost unchanged at the later time points, while mycATR cells have a similar profile when compared to untreated. In acute treatment (0h), all cells (mycATR cells and mycATRΔC-/- cells) are synchronized at G1/S boundary, indicating that the mutation does not affect the response to this level of HU, although in later time points mycATRΔC-/- cells showed an increase of aberrant cells.

Second, in Figure 3E, as the reviewer mentions and supporting these above findings, the restart assay revealed DNA replication to be compromised in mycATRΔC-/- cells, when only a small proportion of cells (5%) incorporated EdU at latest time point after chronic treatment, while 10-12% were detected after acute treatment.

Finally, in Figure 1E, the cells are cultivated in the presence of increasing concentrations of HU, showing that the minimal concentration of 0.1 mM already reduced growth of the mycATRΔC-/- cells by 50%.

In summary, we concluded that low HU concentrations (chronic treatment) allowed a clearer discrimination of the effects of ATR, presumably because the cells are able to maintain DNA replication at this lower level of replication stress.

- The catalytic domain of LmjF.32.1460 is located at 2863-3135 aa according to the NCBI database. The authors deleted 2060-3135 aa and should comment on this.

Thank you for this comment. The selection of the area deleted in our mutants was defined by two conditions for the analysis:

The first was to delete an area that includes not only the catalytic domain (2863 – 3135 aa), but also a FAT domain (2419 – 2666 aa), FATC domain (3176 – 3206 aa) and NLS (2922 – 2932 aa) (PMCID: PMC8377203). We made sure to mention that the catalytic domain was part of the deleted region in the text: Therefore, in our mycATR cells we opted to delete a 3,444 bp region from the C-terminus of ATR (from 6218 – 9624 bp), containing the predicted kinase domain, generating the cell line mycATRΔC-/- (Figure 1A).

The second condition was to find a sequence for the guide RNA that efficiently produces the necessary break for the deletion. The alignment and score criteria are described in PMCID: PMC4513079, which was the determinant of the sequences “f and g” in Table S2 to delete the region (2060 – 3207 aa). An explanation was provided at L671.

- The authors state in the text (line 320-322): ‘Our work above revealed that exposing mycATRΔC-/- cells to chronic replication stress conditions permits DNA replication to progress, while mycATRΔC-/- cells displayed a pronounced impairment in DNA replication after acute HU treatment (Figure 3C)’. According to Figure 3C (and Figure 3E), the marked impairment of DNA replication (S phase block) is more pronounced in the chronic condition. It would be good to change the text accordingly.

Corrected, thank you.

- Authors should include shuffled controls (random genomic regions of equal size) for all metaplots (Fig 6A, B, E and S6).

These have been added, thank you for the suggestion.

- If it is feasible, authors should provide a statistical test for copy number variations (Figure S8) to support the claim that there is a modest increase in CNV levels on several chromosomes in both conditions (line 454 -455).

Also added, thank you.

- Supplementary figure legend appears to contain some errors. It would be beneficial to consider re-checking and correcting these.

Corrected, thank you.

Minor

- One-way ANOWA statistically tests three or more groups of measurements. It would be useful if the authors could indicate which test was used to compare two measurements, as in Figure 3E, Figure 4C-D etc.

Thank you for your suggestion. The data was re-analysed using Unpaired t-tests. A significant difference was identified in Figure S3, which showed the EdU positive mycATRΔC-/- cells have a significant decrease in EdU intensity; we modified the text (L275-279) accordingly. The only data that we used 2way ANOVA for was in Figure 5E; this is a small sample (total number of chromosomes in each replicate), and contains two factors: Time (early and late) and Size (smaller, medium, larger); with the dependent variable being continuous (number of chromosomes), the Turkey's HSD test was used to check the significance difference between Time and Size for specific groups.

- The statistical test between untreated and HU-treated mycATRΔC-/- mutants should also be shown in Figure 3B.

Added, thank you.

- The authors should explain what exactly is shown on the y-axis (replication time) in Figure 5C. Is it the ratio of the average MFA-seq signal for each chromosome to its length? If so, they should include this explanation in the legend of Figure 5C. They should do the same for the average MFA-seq signal and how they quantify MFA signals.

Clarified, thank you. How MFA-seq signal is quantified is explained in L751-770.

- In the text the authors state (line 220-222) 'Microscopy images showed a clear increase of nuclear IdU signal in HU-treated mycATRΔC-/- cells compared with untreated control, and with both treated and untreated mycATR cells (Figure 3A)'. However, mycATR cells are not shown in Figure 3A.

Corrected, thank you.

- Line 351-353 - chromosomes 8 and 31 are not shown in all plots in Figure S5. Chromosome 12 is shown in Figure S5 but not explained in the text.

Corrected, thank you. The figure and the text (357-360) were modified.

- Line 358-359 - MFA seq experiment cannot be compared with the experiment shown in Figure S1B, as two-colour FACS was not performed under chronic HU treatment.

We respectfully disagree. At this point we are comparing the replication timing program in untreated cells and pointing out that alterations in the mutant parasites were not clearly reflected in cell cycle changes or percentage of cells replicating.

- Fig1C - to put the statistical analysis on top of the quantification graph.

Added, thank you.

- Fig 1E, 5D and E – There is no legend at the figure to explain what is being shown.

- Fig. 5B and S4 - to show the chromosome length grid to each plot.

- Line 595 - The amount of DNA should be expressed in µg.

Corrected, thank you.

- Line 1072 -1073 - to determine exactly what is shown in Figure 3D. Are the cells shown from chronic (5 hours) or acute (4 hours) treatment?

Corrected, thank you.

- To specify the dilution of the antibody used in the Methods section. Some data are missing, e.g. antibody dilution in the paragraph: "S phase detection and cell cycle profile using flow cytometry".

Corrected, thank you.

- Sometimes the tagged strain is labelled as mycATR (S2B) and sometimes as 3MATR (S2C) and sometimes as 3xMycATR on the figures. It would be nice if the labelling of the tagged strains (and other strains) were consistent.

Thank you for your comment, which we have clarified: the cells in Fig.S2B and S2C are different (see Table 1)

- In the text explaining the two-colour FACS analysis (lines 167-169) it is written that it is the mycATRΔC+/- strain shown in S1B, but in the S1B figure it is labelled as mycATRΔC-/-.

Corrected, thank you.

- Line 216 - to say how long the cells were grown in the presence of IdU.

Corrected, thank you.

- In the text it is stated that the authors used anti-T. brucei γH2A antiserum for Fig. 4A and B, and in these figures the upper panels of the Western blots are labelled as signals detected with anti-myc?

Corrected, thank you.

- Figure 4E has very low-resolution quality. In panel 1N0K an example of 2N1K is shown below.

Thank you. A new example was taken for 1N0K and the Figure colours were changed to improve resolution and contrast.

- Fig. 4D - The color code of two grey colors (1N1K and 1N2K) needs to be changed to make these colors distinct. The lines of the bars are too thick and hide the color information.

Thank you, the Figure was changed

- Line 372 Are these untreated cells in Figure 5F or 'post chronic HU treatment' cells?

They are 'post chronic HU treatment' cells.

- line 1150 Figure 7A has no figure legend

- Line 1102-1103 - This sentence is probably in the wrong place. In Figure 5A, 10 passages are not shown?

Corrected, thank you.

- There are occasional typos or missing or inconsistent information such as

Line 71 DNA lesions(1,2).

Corrected, thank you.

Line 185 Leishtag.org).

Corrected, thank you.

Line 201 mNG signal. (Figure).

Corrected, thank you.

Line 289 ATR results results

Corrected, thank you.

Line 488 'ATR plays a central role in the cellular response to DNA damage, utilizing its kinase activity is used to regulate numerous repair pathways'

Corrected, thank you.

Line 1025 tio

Corrected, thank you.

Line 1026 At Figure 1 is written (~6220 – 9624 pb) while in the text 'the C-terminus of ATR (from 6180 to 9624 bp)'.

Corrected, thank you.

Line 1067 Acute treatment is sometimes described as 5 mM hydroxyurea (HU) for 6 hours and sometimes as 5 mM hydroxyurea (HU) for 5 hours.

Corrected, thank you.

Line 1076-1078 'Percentage of EdU positive mycATR or mycATRΔC-/- cells in each condition was calculated by the number of stained cells by the number of cells present in each sample'

Corrected, thank you.

Line 1082 'the blort'

Corrected, thank you.

Line 1124-1125 rp1 and rp2 are not indicated at Fig 5F

Corrected, thank you.

1148 6D bottom or left?

Corrected, thank you.

Line 1115 'at the right-top' instead of at the right-bottom

Corrected, thank you.
