# Supplementary material for: ATR, a DNA damage kinase, modulates DNA replication timing in Leishmania major
Source: PLoS Genet. 2025 Nov 24;21(11):e1011899. doi: 10.1371/journal.pgen.1011899 (PMC12677790; doi:10.1371/journal.pgen.1011899)
Supplement: Attachment — Submitted filename: My Review.docx [file pgen.1011899.s017.docx]

The authors made a study of the ATR protein of *L. major*. They failed to make a complete knockout of the ATR gene, but they tagged this gene at the N-terminus. However, the authors generated ATR mutants that lacked the predicted kinase domain at the C-terminus, and they analysed these mutants and challenged them with hydroxyurea treatments. They found that the deletion of the C-terminus reduced the presence of the muted ATR protein in the nuclei. Mutants with deleted C-terminus showed aberrant cell cycle progression, accumulation of ssDNA in the nuclei and increased levels of yH2A indicating increased levels of DNA damage. MFA-seq analysis was performed and the results showed that after ATR C-terminal deletion there are changes in the timing of DNA replication initiation. The authors also showed that deletion of the ATR C-terminus leads to a reduced accumulation of mutations, suggesting that ATR promotes chromosomal instability. The text is generally well written and understandable, but there are some statements that could be improved.

This study is useful for understanding the complex biology of this unicellular eukaryote of medical and veterinary interest. This work is also useful for understanding the processes of DNA replication stress and DNA damage response in this parasite. However, there are several deficiencies in the study that must be addressed to make it suitable for publication.

Major

-	The statement that 'only a single locus per chromosome is consistently detected as the primary site of DNA replication initiation as detected by MFA-seq and DNAscent' is often repeated in the text. However, the MFA-seq methodology used in this study does not have the resolution of origin mapping to make this claim. It may be that there are many sites of DNA replication initiation within the large region that the authors refer to as the primary site of DNA replication initiation. It would be beneficial to consider moderating the interpretation of MFA-seq results to ensure a balanced and nuanced understanding of the findings.

Even more, it would be nice to discuss how and why the origins detected by MFA-seq in tagged mycATR differ from those detected in the WT strain that were previously published, saying that there is only one origin per chromosome in Leishmania (PMCID: PMC4612428). It seems that MFA-seq now detects more than one origin per chromosome even in the *L. major* WT strain (Figure S4).

-	It would be very useful to include a supplementary table with the exact genomic locations of the detected MFA peaks in WT, mycATR and mycATRΔC-/- mutants. The chromosome sizes and lengths of the MFA peaks should also be included in this table. Any statements in the main text about relationships between broader or narrower MFA peaks and chromosome lengths should also be correlated with this supplementary table. The exact positions of the 36 ERRs should also be provided. Otherwise, this section of the text may prove challenging to comprehend.

-	The authors should further discuss the choice of analyzing DNA replication in chronic HU. According to Figure 3C-E, DNA replication is perturbed but recovered after acute conditions, whereas DNA replication seems to be blocked after acute HU treatment (Figure 3C) and the observed minority of cells labelled with EdU could only be cells where DNA repair occurs (less than 5% of cells labelled with EdU in chronic HU condition according to Figure 3E). Furthermore, as shown in Figure 1E, 0.5 mM HU is lethal even for the mycATR strain after 4 days. This concentration is highly toxic and 5 mM used in acute condition is even more toxic. It would be useful if the authors could comment on this.

-	The catalytic domain of LmjF.32.1460 is located at 2863-3135 aa according to the NCBI database. The authors deleted 2060-3135 aa and should comment on this.

-	The authors state in the text (line 320-322): ‘Our work above revealed that exposing mycATRΔC-/- cells to chronic replication stress conditions permits DNA replication to progress, while mycATRΔC-/- cells displayed a pronounced impairment in DNA replication after acute HU treatment (Figure 3C)’. According to Figure 3C (and Figure 3E), the marked impairment of DNA replication (S phase block) is more pronounced in the chronic condition. It would be good to change the text accordingly.

-	Authors should include shuffled controls (random genomic regions of equal size) for all metaplots (Fig 6A, B, E and S6).

-	If it is feasible, authors should provide a statistical test for copy number variations (Figure S8) to support the claim that there is a modest increase in CNV levels on several chromosomes in both conditions (line 454 -455).

-	Supplementary figure legend appears to contain some errors. It would be beneficial to consider re-checking and correcting these.

Minor

-	One-way ANOWA statistically tests three or more groups of measurements. It would be useful if the authors could indicate which test was used to compare two measurements, as in Figure 3E, Figure 4C-D etc.

-	The statistical test between untreated and HU-treated mycATRΔC-/- mutants should also be shown in Figure 3B.

-	The authors should explain what exactly is shown on the y-axis (replication time) in Figure 5C. Is it the ratio of the average MFA-seq signal for each chromosome to its length? If so, they should include this explanation in the legend of Figure 5C. They should do the same for the average MFA-seq signal and how they quantify MFA signals.

-	In the text the authors state (line 220-222) ‘Microscopy images showed a clear increase of nuclear IdU signal in HU-treated mycATRΔC-/- cells compared with untreated control, and with both treated and untreated mycATR cells (Figure 3A)’. However, mycATR cells are not shown in Figure 3A.

-	Line 351-353 - chromosomes 8 and 31 are not shown in all plots in Figure S5. Chromosome 12 is shown in Figure S5 but not explained in the text.

-	Line 358-359 - MFA seq experiment cannot be compared with the experiment shown in Figure S1B, as two-colour FACS was not performed under chronic HU treatment.

-	Fig1C - to put the statistical analysis on top of the quantification graph.

-	Fig 1E, 5D and E – There is no legend at the figure to explain what is being shown.

-	Fig. 5B and S4 - to show the chromosome length grid to each plot.

-	Line 595 - The amount of DNA should be expressed in μg.

-	Line 1072 -1073 - to determine exactly what is shown in Figure 3D. Are the cells shown from chronic (5 hours) or acute (4 hours) treatment?

-	To specify the dilution of the antibody used in the Methods section. Some data are missing, e.g. antibody dilution in the paragraph: "S phase detection and cell cycle profile using flow cytometry".

-	Sometimes the tagged strain is labelled as mycATR (S2B) and sometimes as 3MATR (S2C) and sometimes as 3xMycATR on the figures. It would be nice if the labelling of the tagged strains (and other strains) were consistent.

-	In the text explaining the two-colour FACS analysis (lines 167-169) it is written that it is the mycATRΔC+/- strain shown in S1B, but in the S1B figure it is labelled as mycATRΔC-/-.

-	Line 216 - to say how long the cells were grown in the presence of IdU.

-	In the text it is stated that the authors used anti-*T. brucei* γH2A antiserum for Fig. 4A and B, and in these figures the upper panels of the Western blots are labelled as signals detected with anti-myc?

-	Figure 4E has very low-resolution quality. In panel 1N0K an example of 2N1K is shown below.

-	Fig. 4D - The color code of two grey colors (1N1K and 1N2K) needs to be changed to make these colors distinct. The lines of the bars are too thick and hide the color information.

-	Line 372	Are these untreated cells in Figure 5F or 'post chronic HU treatment' cells?

-	line 1150	Figure 7A has no figure legend

-	Line 1102-1103	- This sentence is probably in the wrong place. In Figure 5A, 10 passages are not shown?

-	There are occasional typos or missing or inconsistent information such as

Line 71	DNA lesions(1,2).

Line 185	Leishtag.org).

Line 201	mNG signal. (Figure).

Line 289	ATR results results

Line 488	‘ATR plays a central role in the cellular response to DNA damage, utilizing its kinase activity is used to regulate numerous repair pathways’

Line 1025	tio

Line 1026	At Figure 1 is written (~6220 – 9624 pb) while in the text ‘the C-terminus of *ATR* (from 6180 to 9624 bp)’.

Line 1067	Acute treatment is sometimes described as 5 mM hydroxyurea (HU) for 6 hours and sometimes as 5 mM hydroxyurea (HU) for 5 hours.

Line 1076-1078	‘Percentage of EdU positive mycATR or mycATRΔC-/- cells in each condition was calculated by the number of stained cells by the number of cells present in each sample’

Line 1082	‘the blort’

Line 1124-1125	rp1 and rp2 are not indicated at Fig 5F

1148	6D bottom or left?

Line 1115	‘at the right-top’ instead of at the right-bottom
