# Supplementary material for: ATR, a DNA damage kinase, modulates DNA replication timing in Leishmania major
Source: PLoS Genet. 2025 Nov 24;21(11):e1011899. doi: 10.1371/journal.pgen.1011899 (PMC12677790; doi:10.1371/journal.pgen.1011899)
Supplement: Attachment — Submitted filename: Response to the Reviewrs_2.docx [file pgen.1011899.s020.docx]

**ATR, a DNA damage kinase, modulates DNA replication timing in *Leishmania major*.**

Corresponding Authors: Professor Richard McCulloch and Professor Luiz R. O. Tosi

**We again thank all reviewers for their comments and suggestions. Detailed responses to each reviewer’s comments are provided in red text, and below is a summary of the further changes we have made:**

- **Figure 3A and 3D.** The pictures were amplified and displayed in two ways: a population view, and a zoom of a single cell to show the effect described in the text. Labels were also moved outside the figures to improve visualisation.
- **Figure 4E.** As explained above, the pictures were amplified and zoomed at a cell level to better visualise the nucleus and kinetoplast. In addition, the figure was simplified by showing only two channels (DAPI and DAPI/β-tubulin).
- **Table S1:** The three categories were divided by colour to allow them to be better distinguished, and a new legend is provided: ‘Table detailing all 36 ERRs described in (64 and 65) showing the start, end and length of the region (Green); regions described in Figure 6D where we observed the overlap between SSR and the two types of repetitive sequences, SIDER1 or 2, used to generate the metaplots in Figures 6E and Figure S9C, and showing the start, end and length (Orange) as well highlighted regions where the signal is stronger; regions described in Figure 6D where we observed the overlap between the two types of repetitive sequences, SIDER1 and 2, used to generate the metaplots in Figures 6E and Figure S9C, showing the start, end and length (Red).
- **Figures 5A and 7A:** legends were amended to mention BioRender**.**

Journal Requirements:

1) Please make sure to update the legends for Figures 5A and 7A to specify that they were created using BioRender, rather than including this information in the acknowledgments.

Corrected, see summary

Reviewers' comments:

Reviewer's Responses to Questions

Comments to the Authors:

Please note here if the review is uploaded as an attachment.

Reviewer #1: This manuscript was originally reviewed with the recommendation to revise. Upon re-review of the revised manuscript, I find that the minor comments (figure mis-labelling, text corrections, elucidation of the deleted region of ATR in Fig 1, etc.) have mostly been addressed. Note that this has not always been done accurately: as examples:

a) the CNV data (originally Fig S8, now moved to main Fig 7) is labelled D in the Fig 7, but there is no D in the legend. I think it should be C?

Corrected, Thank you

b) Low resolution pictures in Fig 4 were raised by several reviewers: these have been replaced, but with pictures that reduce the cell-staining resolution and only slightly improve the DNA resolution.

New versions of the figures have been provided, please see the summary above.

Most of the more substantive comments in my original review have not been addressed, with the authors respectfully disagreeing, or side-stepping the issue (such as on counterintuitive SNP accumulation, or on the semantics of ‘dormant’ or ‘stochastic’ origins – which, however termed, are poorly detected by MFAseq). Most importantly, some issues have been addressed with added data that only raise further concerns.

Accordingly, the editor should decide whether a somewhat tidied-up but fundamentally largely unchanged manuscript can be published.

To expand on the main outstanding points:

The major suggestion that MFAseq has insufficient resolution (also noted by Reviewer 3) has not been addressed by additional work with DNAscent or combing. This was expected, but as it stands, the text still fails to highlight the very marked shortcomings of the MFAseq technique used alone in this context.

Thank you for your comment. We note that any additional work (DNAscent or combing) was stated as being non-mandatory by the reviewer: “These are probably not, however, practical experiments to do or re-do at the revision stage”. However, we agree that we have not reflected on the differences between MFAseq and DNAscent, and the limitations of the former, and so we have added to the discussion:

L630 and onwards “It should be noted that MFA-seq is a population-level analysis and has relatively low resolution, since potential sites of initiation are inferred as the summit of much wider peaks of sequence enrichment in replicating cells relative to non-replicating. For this reason, what we describe here may be only the most prominent new sites of replication initiation in ATR mutants or after HU treatment, and MFA-seq may overlook wider initiation loci, such as has been recently detected in wild type *L. major* by single-molecule DNAscent analysis (Damasceno et al BioRxiv 10.1101/2024.11.14.623610). Irrespective, one explanation….’’.

L655-656 “However, no work to date has described the localisation of ORC in any *Leishmania* genome.”

The other major point – that the two ATR-mutant clones c1 and c8 appear to differ greatly in multiple experiments – is only minimally addressed (by an added comment in the text: ‘and randomness in the loci at which they are activated could explain the differences in MFA-seq profiles observed between the mutant clones.’) The concern remains, therefore, that the reported phenotypes are substantially affected by secondary adaptations in different clones. Even more concerningly, where data from 2 technical replicates on the same clones are now added (e.g. Fig S4, this in response to a related concern on this same point, raised by Reviewer 2), it becomes clear that even technical replicates vary hugely, particularly in cl8. The paper hinges on phenotypes of these 2 clones, so their inconsistency (both inter-clone, and even more so, inter-repeat) is a real concern.

Thank you for your comment. The differences between the MFA-seq profile in each chromosome (including the replicas) of the mutant clones in untreated conditions (Fig. S4) is, indeed, a concern and the only explanations that we can provide at this moment are, as stated, secondary adaptations, or randomness of the loci at which DNA replication arises in the mutants. However, we would like to highlight that the overall effect on the DNA replication program is highly consistent in both replicates of each mutant clone (Figure 5C and Fig. S5), demonstrating that, regardless of variation in the change of MFA-seq signal between experiments and clones, the outcome in terms of the timing of genome duplication is the same. In addition, it is important to note that the MFA-seq profile *per* chromosome in each replicate of each mutant clone is very consistent after the addition of replicative stress (0 and 5 hours), as showed in Figure S8, clearly indicating that ATR is important for DNA replication programming under such conditions.

To encapsulate these considerations, we have further modified the text (L643 and onwards):

“Irrespective, one explanation for these new MFA-seq signals is that they represent normally suppressed origins, which are activated in the absence of ATR with and without excess replication stress caused by HU, and randomness in the loci at which they are activated could explain the differences in MFA-seq profiles observed in each chromosome in untreated conditions between the mutant clones (Figure S4). Such a view would align with known functions of ATR in other eukaryotes, where licensed but not activated origins are controlled by ATR signalling (88), which acts as a negative regulator of origin firing and prevents inappropriate activation of origin firing in unperturbed cells (89), functions that are important to guarantee a sufficient supply of DNA precursors and replication factors for optimal fork progression (89). Interestingly, such control seems to be particularly important during and after replicative stress in *Leishmania*, since the MFA-signal around all loci (overlap between SSR/SIDER1/SIDER2 and ERR) and the MFA-seq profiles in each chromosome was consistent among the mutant clones (Figure 6E and Figure S8). So far, there has been little evidence for activation of ‘dormant’ origins in trypanosomatids in response to replication stress; in *T. brucei*, only one single putative site in one chromosome was found (90). However, although all SSRs appear to bind one subunit of ORC, only ~25% are activated during unperturbed growth (91), and so SSRs may be a likely location for dormant origins that would be used in response to replication stress. However, no work to date has described the localisation of ORC in any *Leishmania* genome.’’

Reviewer #2: The authors have responded to most of my concerns. The last part, about SNVs and indels, remains weaker than the rest of the manuscript. While improved from the initial submission, the authors could include the benchmark data they cited in their response as part of the QC for their current analysis. The different patterns of SNVs vs indels are still sensitive to the experimental design (specifically, just 10 generations), and their suggestion that SNVs are less harmful than indels and that a DDR defect may modulate mutation patterns with respect to these different levels of effect, is contested. The authors should present their results more carefully, avoid unsupported interpretations, and note the possible caveats of this experiment.

We appreciate of the reviewer’s comments, but we continue to defend our approach:

First, and respectfully, we do not understand what the reviewer means by “benchmark data”; as explained in our previous response, and as detailed at *Methods*, the *FreeBayers*, VCF filter and VCF-VCF filter are part of Deeptools package, which was run using the Galaxy platform: L815-816 “The Galaxy web platform (usegalaxy.org)(100) was used for most of the downstream data processing”. Here we set up the QC for each sample, for example: number of supporting reads and minimal coverage.

Second, as we explained in our previous response, the 10 passage experimental set up (which represents 30 generations, L511) was decided based on published literature (PMID: 32609721, used 6 passages; PMID: 39922816, used 7 passages; PMID: 30380080, after treatment; PMID: 30401775, used 5 passages) and in all of these studies, the detection of SNPs/SNVs and InDels were performed as we have done here.

Finally, while we recognise that further experiments are needed to understand the mechanistic basis of differences we observe in SNPs/SNVs and InDels, we show that the presence or absence of functional ATR alters the balance of such changes. In a paper that is focused on how ATR acts in *Leishmania*, we feel that such information is important to share with the community.

To more clearly articulate the limitations of the analysis, we have changed the discussion as follows (L531 and onwards):

“With these data we can correlate the accumulation of SNVs (which are potentially less harmful and more tolerated in eukaryotes (74,75, including *Leishmania* (76–78)) with the presence of an efficient DDR response (mycATR cells), while an increase of InDels (potentially more disruptive because they may cause frameshift mutations) (73,79,80) results from a DDR defect caused by the absence of a functional ATR, which might involve activation of alternative DNA repair pathways. However, further characterisation is needed to understand the mechanisms of DNA repair that generate such genome variations in *Leishmania*.’’

Reviewer #3: The authors made substantial modifications to the manuscript in order to address the reviewers' comments and clarify certain results. However, they did not complete Supplementary Table S1 as requested. This table was supposed to list all the MFA-seq peaks detected in the wild-type (WT), mycATR and mycATRΔC-/- mutants. In this table, the authors now provide the locations and lengths of the ERR, SSR/SIDER1/SIDER2 and SIDER1/SIDER2. They do not specify which ERRs are shown. I assume these are from the WT strain, since the ERRs from the mycATRΔC⁻/⁻ mutants are not always in these positions (see Figure S4). The MFA-seq results from two replicates often differ significantly (Figure S4). The authors did not specify whether the two replicates presented in Figure S4 were normalised. If so, how can we explain the loss of some of the most intense signals present in one replicate but absent in the other after merging the two replicates?

Thank you for your comment.

For the differences in signal on ERRs from the mycATRΔC⁻/⁻ mutants, please check the response to Reviewer #1. In terms of normalisation, all samples were normalised in the same way, as explained below in response to the question about Chromosome 01 and FACS sorting.

The table was modified as detailed in the summary.

To address the reviewer’s questions: the 36 ERR are the early replication regions described in PMID: 32897188 and PMID: 26481451; such regions were added to the table in response to the reviewer’s request (“The exact positions of the 36 ERRs should also be provided”). These regions are those displayed in Figure 5B and Figure 6C and were used to generate the metaplots in Figure 6A and B (and in related supplementary figures). These results, and especially the metaplots that show the global signal for all samples (wild-type (WT), mycATR and mycATRΔC-/- mutants), demonstrate that the MFA-seq signal around the ERR is stronger than any other signal in the genome, indicating that these regions are early activated, consistent with the literature cited above. In addition, the standard deviation in each metaplot shows that, beside some differences in the signal around these ERRs in some chromosomes, ATR mutation or HU treatment did not abrogate the primacy of MFA-seq signal from these loci, reinforcing that they are earliest replicating genome features (Figure 6A).

However, we agree that it is important to provide the location of the new regions that we demonstrated to have MFA-seq signal outside ERRs, specially after replicative stress which are: Overlap Regions of SSR and repetitive sequences (SIDER1 and 2) and Overlap Regions of SIDER1 and 2. Those are listed in the table and are now described at the legend. We have also now highlighted the regions where we see more MFA-seq signal in Overlap Regions of SSR and repetitive sequences (SIDER1 and 2).

The authors demonstrate the location of SSR-Oris at EERs. I assume they presume these are the origins of DNA replication. However, they do not explain how they identified SSR-Oris’ locations. Did they use MFA-seq or DNAscent data? Of the DNAscent molecules, how many showed replication cantered on the SSRs, and how many did not? These details must be specified to prevent misinterpretation of the positions of origins.

Thank you for your comment. That is correct: the SSR-ORI at EERs are predicted origins of replication that were first described by MFA-seq (PMID: 32897188, PMID: 26481451), and have also now been validated as the primary sites of replication initiation by single-molecule DNAscent (BioRxiv **doi:** <https://doi.org/10.1101/2024.11.14.623610>). The MFA-seq signal around each of the SSR-ORIs is shown in Figure 6A and Figure S6; the heatmaps in each row represent one chromosome, and the signal was plotted around the centre the SSR-ORI, as described at L444-447 “we generated metaplots of MFA-seq signal in mycATR and mycATRΔC-/- cells. In the absence of HU, we detected no quantifiable differences in MFA-seq signal between mycATR and mycATRΔC-/- cells at the 36 ERRs, whether analyzed collectively or separated by chromosome size (Figure S6A and S6B)”. We also modified the legend to clarify that they represent the 36 SSR-Oris (one per chromosome).

The authors state that ‘Chromosome 1 was excluded because an amplification in copy number was found in our stationary control sample’. This is interesting, given that the same group previously demonstrated an MFA-seq signal for chromosome 1 (PMID: 26481451). The authors should discuss how the amplification of chromosome 1 can occur in the stationary control sample and explain what this amplification means.

Thank you for the comment. The stationary control used in (PMID: 26481451) was not the one used on this work. For each MFA-seq experiment, we prepared a matched stationary control (here, the Cas9T7 cell line that was used to generated the ATR modifications). These details are explained in the legend of Figure 5A.

To answer the last question, unfortunately chromosome amplification happens frequently in *Leishmania*, since it is an organism with a very plastic genome that causes stochastic fluctuation in copy number of chromosomes in the population. In terms of how that influences the MFA-seq signal, we rely on the ratio of reads mapping to chromosomes in replicating versus non-replicating (stationary phase) cells; if the stationary sample has an increase in the ploidy of a chromosome that is not present in the replicating cells, the result is a negative MFA-seq signal across the whole chromosome. Below is an image of the MFA-seq for chromosome 1:

If the authors performed FACS cell sorting of Leishmania for MFA-seq, they should specify this in the Methods section, along with how they did it.

We did not perform FACS cell sorting. The approach, detailed above, was based on the latest MFA-seq protocol (PMID: 32897188) and is explained at the *Methods* L821-827 “BamCompare (DeepTools) was used to determine read abundance from exponentially growing cells or after HU treatment relative to the reads from stationary culture. Ratios (MFA-seq signal) were first calculated in 500 bp consecutive windows using the reads counts method for normalisation; 169 areas with poor mapping where excluded (67). Raw ratios were further transformed into Z scores relative to the whole genome ratio average as calculated in 100 kb sliding windows”.

The authors did not provide any proof of DNA replication restart and misinterpreted the data on EdU incorporation (Figure 3E). They demonstrated that EdU was still being incorporated following the acute or chronic HU treatment of mycATR and two mycATRΔC-/- mutants. This could be due to recombination, DNA repair, or replication. The restart of DNA replication can be demonstrated using DNA combing or DNA fiber stretching experiments. Therefore, the authors should remove the following sentence:

Line 277-278 – ‘Together, these data demonstrate that ATR is crucial for efficient DNA replication restart in L. major.’

Thank you for your comment and we agree: we cannot distinguish where the signal comes from without proper markers for each pathway. We have therefore modified the text to accommodate all possibilities for the DNA synthesis predicted by EdU (L315-317):

“In addition, the nuclear signal of EdU positive cells also showed an overall difference between mycATR and mycATRΔC-/- cells, suggesting that mechanisms involving DNA synthesis, such as DNA replication, DNA repair and DNA recombination, are affected after replication stress in cells with mutated ATR (Figure S3).’’

We also modified the title of the section and the legend of the figures:

“Replication stress leads to ssDNA accumulation, which affects the mechanisms influencing cell cycle progression in mycATRΔC-/- cells.”

Minor:

Even in high-resolution, the quality of Figures 3D and 4E is poor.

New Figures were provided, please see summary above.

The line weights in Figure 4F should be reduced to make the borders between the different categories more distinguishable.

Corrected, thank you.

It seems that there is an error in the S3 legend. Is it the ssDNA or the EdU signal intensity?

Corrected, thank you.

Table S1. - The labelling of the columns is confusing (e.g. column SSR, SIDER1 SIDER2 vs column SIDER1 SIDER2?). A more detailed explanation is required.

Figure was modified, along with its legend, for a clearer explanation.

The title of Table S2 is 'Table 1'.

Corrected, thank you.
