# Supplementary material for: ATR, a DNA damage kinase, modulates DNA replication timing in Leishmania major
Source: PLoS Genet. 2025 Nov 24;21(11):e1011899. doi: 10.1371/journal.pgen.1011899 (PMC12677790; doi:10.1371/journal.pgen.1011899)
Supplement: Attachment — Submitted filename: Response to the Reviewers_3.docx [file pgen.1011899.s021.docx]

**ATR, a DNA damage kinase, modulates DNA replication timing in *Leishmania major*.**

Corresponding authors: Professor Richard McCulloch and Professor Luiz R. O. Tosi

**We again thank all reviewers for their comments and suggestions.**

**Editorial comment:** have all data underlying the figures and results presented in the manuscript been provided?
Large-scale datasets should be made available via a public repository as described in the *PLOS Genetics* [data availability policy](https://eur03.safelinks.protection.outlook.com/?url=http%3A%2F%2Ftrack.editorialmanager.com%2FCL0%2Fhttp%3A%252F%252Fjournals.plos.org%252Fplosgenetics%252Fs%252Fdata-availability%2F2%2F010f01985b8ad949-9de94446-05cf-405a-a7ff-4579f2c96e16-000000%2F59nCKGBwHG0fUzrjypWmNYEPL6oNF2eIoG2hZ-rWmBw%3D222&data=05%7C02%7CGabriel.AlmediaDaSilva%40glasgow.ac.uk%7C47953a9eec3646d6681a08ddcf6dd8b3%7C6e725c29763a4f5081f22e254f0133c8%7C1%7C0%7C638894792889144608%7CUnknown%7CTWFpbGZsb3d8eyJFbXB0eU1hcGkiOnRydWUsIlYiOiIwLjAuMDAwMCIsIlAiOiJXaW4zMiIsIkFOIjoiTWFpbCIsIldUIjoyfQ%3D%3D%7C0%7C%7C%7C&sdata=%2BNnUvMJiyFEFwIg661NgNnBbgRXDhY86contf6M4kO8%3D&reserved=0), and numerical data that underlies graphs or summary statistics should be provided in spreadsheet form as supporting information.

**Response:**

**We provide new supporting data, accumulated in Supplementary file 1.** Here, we provide all data underlying the main and supplementary figures:

**In addition, we note again that the raw data for the MFA-seq analysis is available:** The raw data (*.fasta.gz files) from the MFA-seq and the *.bedgraph files used to generate the Metaplots for this study are available at European Nucleotide Archive (ENA) at EMBL-EBI under accession number PRJEB83661.
