# Supplementary material for: ATR, a DNA damage kinase, modulates DNA replication timing in Leishmania major
Source: PLoS Genet. 2025 Nov 24;21(11):e1011899. doi: 10.1371/journal.pgen.1011899 (PMC12677790; doi:10.1371/journal.pgen.1011899)
Supplement: Attachment — Submitted filename: Response to the Reviewers_4.docx [file pgen.1011899.s022.docx]

**ATR, a DNA damage kinase, modulates DNA replication timing in *Leishmania major*.**

Corresponding authors: Professor Richard McCulloch and Professor Luiz R. O. Tosi

**There are no reviewer’s comments, and so below we respond only to an editorial request.**

**Editor's comments:**

You current supplementary section included into version that we inspected is just a set of images combined into a single pdf file. *Please, have all numeric data supporting conclusions from your analyses and graphs and diagrams in your main and supplementary display items presented in spreadsheet format, i.e., excel or tab-delimited text. You current supplementary pdf also includes the image of a computer script, which makes it impossible for verification by others without unnecessary effort of retyping it.* Computer script should be provided as tab-delimited text file. If you decide to re-submit PLOS Office staff and then Editors will examine if reformatted supplementary can be used by members of scientific community to verify your conclusions, compare with data from studies ,and to explore independent hypotheses.

Please, note that PLOS explicitly states in submission guidelines:

For smaller data sets and certain data types, authors may provide their data within supporting information files accompanying the manuscript. Authors should take care to maximize the accessibility and reusability of the data by selecting a file format from which data can be efficiently extracted (for example, spreadsheets or flat files should be provided rather than PDFs when providing tabulated data).

**Response:**

**We now provide 3 supplementary files:**

**Supplementary File 1.** Numeric data underlying main and supplementary figures, which are identified by tabs.

**Supplementary File 2.** Western blot and flow cytometry data underlying Figures 1, 3 and 4.

**Supplementary File 3.** Text file of script for sequence analysis
